# Supplementary material for: The mitochondrial pyruvate carrier regulates memory T cell differentiation and antitumor function
Source: Cell Metab. 2022 May 3;34(5):731–746.e9. doi: 10.1016/j.cmet.2022.03.013 (PMC9116152; doi:10.1016/j.cmet.2022.03.013)

Cell Metabolism, Volume *34*

Supplemental Information

The mitochondrial pyruvate carrier regulates memory T cell differentiation and antitumor function

Mathias Wenes, Alison Jaccard, Tania Wyss, Noelia Maldonado-Pérez, Shao Thing Teoh, Anouk Lepez, Fabrice Renaud, Fabien Franco, Patrice Waridel, Céline Yacoub Maroun, Benjamin Tschumi, Nina Dumauthioz, Lianjun Zhang, Alena Donda, Francisco Martín, Denis Migliorini, Sophia Y. Lunt, Ping-Chih Ho, and Pedro Romero

Supplemental Information

Supplemental figure legends

Figure S1. MPC inhibition in CD8$^+$ T cells induces alternative metabolic fluxes that results in an epigenetic crosstalk and memory imprinting

(A-B) Schematic presentation of the culture conditions for metabolomics analysis (A). Naïve CD8$^+$ T cells were activated with anti-CD3/CD28 antibodies in the presence of DMSO or UK5099 (MPCi). After 72 hours, the cells were harvested for untargeted metabolomics analysis by mass spectrometry. All detected metabolites are shown and scaled to the average of the DMSO samples (B). "(NG)" before a metabolite means, no confident reliable detection was possible ("Not Good"). (n = 4 biological replicates)

(C-H) Fresh naïve CD8$^+$ T cells were activated with anti-CD3/CD28 antibodies in the presence of DMSO or MPCi. After 66 hours, T cells were washed and incubated with medium containing either 11mM U-^{13}C-glucose, 4mM U-^{13}C-glutamine, or 120 μM U-^{13}C-Palmitate. 6 hours later, ^{13}C incorporation was measured by mass spectrometry. Shown here are the isotopic ratios (C-E) and labelled abundances (G-H) of citrate. ('m+n' equals the molecular mass plus the number of incorporated heavy carbons). (n = 4 biological replicates/group)

Data is represented as mean ± standard error of mean. Statistics are based on two-way ANOVA using the original false discovery rate method of Benjamini and Hochberg, with *p < 0.05, **p < 0.01, ***p < 0.001, ****p < 0.0001 and ns (p > 0.05).

Figure S2. MPC inhibition in CD8$^+$ T cells induces alternative metabolic fluxes that modulate chromatin epigenetic modifications and memory imprinting

(A) OT1 splenocytes were activated with SIINFEKL and IL2 in the presence of DMSO or UK5099 (MPCi). After 72h, protein was collected for western blot analysis of acetylated lysine residues on whole cell lysate, and beta-actin as loading control. Shown here is the same blot with either short or long exposure time allowing for a proper discrimination of all bands. The estimated histone region is indicated (containing histones H2A/B, H3 and H4).

(B-C) Representative western blot (B) and quantification (C) of modified lysine residues on histone 3, normalized for total histone 3 (H3) protein and shown as fold change compared to DMSO. (n = 2 biological replicates, pooled data from 2 independent experiments)

(D-E) Isotopic distribution of acetylated peptide KSAPATGGVKKPHR (containing H3K27) at *m/z* 529.975 with ^{12}C (D) or ^{13}C (E) glucose as substrate. Non-acetylated lysines were propionylated. Monoisotopic peak (containing only ^{12}C) and 3rd isotopic peak (2*^{13}C) were used for measurement of ^{13}C incorporation into the peptide due to the acetylation. The increase of other isotopic peaks at higher m/z indicates that ^{13}C incorporation can occur not only because of the acetylated group, but also in the peptide backbone, although at a lower level.

(F) Volcano plot from ATAC sequencing data showing the genes associated to more closed (left) or more open (right) chromatin regions upon MPCi treatment. Genes appearing in the KEGG pathways fatty acid oxidation (FAO), glutamine oxidation (GO), pentose phosphate pathway (PPP), glycolysis and oxidative phosphorylation (oxphos) are highlighted in color.

Data is represented as mean ± standard error of mean. Statistics are based on two-way ANOVA using the original false discovery rate method of Benjamini and Hochberg, with ns ($p > 0.05$).

Figure S3. Runx1 orchestrates CD8$^+$ T cell memory differentiation upon mitochondrial pyruvate carrier inhibition

(A) CAS9-expressing CD8$^+$ OT1 T cells were activated and transduced with either scrambled small guiding RNA (gRNA SCR) constructs or a pool of 3 different gRNA's targeting *Runx1*. 48 hours after transduction, T cells were further expanded in either DMSO or 20μM UK5099 (MPCi). 7 days post-transduction, western blot was performed on the whole cell lysate. Total histone H3 was used as loading control. (representative western blot of 2 independent experiments with 1 biological replicate each)

Figure S4. Mitochondrial pyruvate carrier deletion in CD8$^+$ T cells blunts their anti-tumor potential

(A-D) Tumor and spleen single cell suspensions were restimulated *ex vivo* with SIINFEKL for 4 hours. Transferred WT or MPC1 KO CD8$^+$ T cells were assessed for expression of cytokines or CD107a by flow cytometry. Data is expressed as percentage out of transferred cells (A-D) or absolute number of cells per mg of tumor (E-J) (n = 16 mice/group, pooled data from 3 independent experiments (A-I) and n = 10-12 mice/group, pooled data from 2 independent experiments (J))

Data is represented as mean ± standard error of mean. Statistics are based on two-way ANOVA using the original false discovery rate method of Benjamini and Hochberg (A-D), or on unpaired, two-tailed Students t-test (E-J) with *$p < 0.05$, **$p < 0.01$, ***$p < 0.001$, ****$p < 0.0001$ and ns ($p > 0.05$).

Figure S5. Tumor-infiltrating CD8$^+$ T cells oxidize lactate in their mitochondria to maintain effector function

(A) Schematic presentation of the *in vitro* experimental modelling of a tumor nutrient-deprived microenvironment. WT or MPC1 KO OT1 splenocytes were activated with SIINFEKL and IL2. Cells were washed and split after 3 days, and then cultured for another 3 days. The T cells were then collected, counted and placed in nutrient-rich or nutrient-deprived medium with varying concentrations of sodium lactate, or sodium chloride as osmotic control for 18 hours and then restimulated with SIINFEKL for 4 hours.

(B) Viability of the 18h starved WT and MPC1 KO CD8$^+$ T cells was analyzed by flow cytometry before and 4 hours after restimulation with SIINFEKL. Shown here is the percentage of live cells, identified as AnnexinV-negative and negative for the dead cell staining dye. (n = 4 biological replicates/genotype, pooled data from 2 independent experiments)

(C-E) Resting antigen-experienced WT or MPC1 KO OT1 CD8$^+$ T cells were placed in nutrient-rich or nutrient-deprived medium with varying concentrations of lactate for 18 hours and then restimulated with SIINFEKL for 4 hours. T cells expressing IFNγ (C), TNF (D) or IL2 (E) were analyzed by flow cytometry. (n = 5 biological replicates/genotype, pooled data from 2 independent experiments)

(F) Naïve CD8$^+$ T cells were isolated from spleens of WT or MPC1 KO OT1 mice and were activated with anti-CD3/CD28-coated beads for 3 days and then rested for an additional 3 days. The T cells were then placed in nutrient-rich or nutrient-deprived medium with varying concentrations of lactate for 18 hours and then restimulated with SIINFEKL for 4 hours. T cells expressing IFNγ, TNF and IL2 were analyzed by flow

cytometry. (n = 2 biological replicates/genotype, pooled data from 2 independent experiments)

(G-J) Resting antigen-experienced WT $CD8^+$ T cells were placed in nutrient-deprived medium with or without lactate for 18 hours in the presence of 25μM LDHA/B inhibitor, GSK 2837808A (LDHi), or DMSO control and then restimulated with SIINFEKL for 4 hours. T cells expressing IFNγ (G), TNF (H), IL2 (I) or co-express both IFNγ and TNF (J) were analyzed by flow cytometry. (n = 2 biological replicates/group, pooled data from 2 independent experiments)

(K) Resting antigen-experienced WT or MPC1 KO $CD8^+$ T cells were placed in nutrient-rich or nutrient-deprived medium with or without lactate and varying concentrations of the mTOR inhibitor Torin2 for 18 hours and then restimulated with SIINFEKL for 4 hours. Western blot analysis shows mTOR pathway activity. Different phosphorylation status of 4E-BP1 results a separation in 3 obvious bands with the α-form being unphosphorylated and the γ-form most phosphorylated. (Representative blot from 3 independent experiments with 1 biological replicate/experiment)

(L-O) Resting antigen-experienced WT or MPC1 KO $CD8^+$ T cells were placed in nutrient-rich or nutrient-deprived medium with or without lactate and varying concentrations of the mTOR inhibitor Torin2 for 18 hours and then restimulated with SIINFEKL for 4 hours. T cells expressing IFNγ (L), TNF (M), IL2 (N) or co-express both IFNγ and TNF (O) were analyzed by flow cytometry. (n = 2 biological replicates/genotype, pooled data from 2 independent experiments)

Data is represented as mean ± standard error of mean. Statistics are based on two-way ANOVA using the original false discovery rate method of Benjamini and Hochberg, with *p < 0.05, **p < 0.01, ***p < 0.001, ****p < 0.0001 and ns (p > 0.05). For clarity, only relevant statistical comparisons are shown on graphs.

Figure S6. Mitochondrial pyruvate carrier inhibition during CAR T cell *in vitro* activation and expansion induces superior anti-tumor activity upon adoptive cell transfer in a mouse melanoma model

(A) CD45.1$^+$ OT1 CD8$^+$ T cells were activated and expanded for 7 days with DMSO or 20μM UK5099 (MPCi) (called here "conditioning", in preparation for ACT), after which the small molecule was washed out and the cells were then transferred into B16-SIINFEKL tumor-bearing CD45.2$^+$ mice. At the point of adoptive cell transfer, mice received a subcutaneous administration of a vaccine consisting out of SIINFEKL and synthetic oligodeoxynucleotides containing CpG motifs. Shown here is the tumor weight measured upon dissection at day 26.

(B) Total number of transferred OT1 CD8$^+$ T cells in the tumor-draining lymph node, measured by flow cytometry.

(C-E) Percentage of tumor infiltrating transferred OT1 CD8$^+$ T cells expressing PD1 (C), LAG3 (D) or TIM3 (E), measured by flow cytometry.

(F-K) Spleen single cell suspensions were restimulated with SIINFEKL *in vitro* for 4 hours in the presence of Golgi inhibitors. The percentage of transferred cells expressing cytokines and granzyme B (F, H, J) or their absolute numbers (G, I, K) were measured by flow cytometry.

(L-N) Tumor single cell suspensions were restimulated with SIINFEKL *in vitro* for 4 hours in the presence of Golgi inhibitors. The percentage of transferred cells expressing cytokines and granzyme B (L, M) or their absolute numbers (N) were measured by flow cytometry. ((A-N) n = 11 mice (DMSO) and 14 mice (MPCi), pooled data from 2 independent experiments.)

(O) Schematic representation of the experiment. Polyclonal CD8$^+$ T cells were activated in the presence of DMSO or 20μM UK5099 (MPCi) and transduced with either a control blue fluorescent protein-expressing vector (BFP) or an anti-HER2 CAR. The T cells were expanded for 7 days in the presence of DMSO or MPCi and then transferred into mice bearing HER2-expressing B16 tumors.

(P) Transduction efficiency measured by flow cytometry of polyclonal CD8$^+$ T cells with an anti-HER2 CAR in the presence of DMSO or MPCi.

(Q) At the time of adoptive cell transfer, histones from DMSO- or MPCi-conditioned HER2 CAR T cells were analyzed by Western blot.

(R-S) Number of DMSO- or MPCi-treated HER2-CAR-positive cells (R) and their percentage of short-lived effector cells (S) in the blood 12 days after adoptive cell transfer, measured by flow cytometry. (n = 12-13 mice (DMSO- and MPCi-treated HER2-CAR T cell transfer), pooled data from 2 independent experiments.)

Data is represented as mean ± standard error of mean. Statistics are based on unpaired, two-tailed Students t-test, with *$p < 0.05$, **$p < 0.01$, and ns ($p > 0.05$).

Figure S7. Mitochondrial pyruvate carrier inhibition induces memory differentiation in human T cells and dramatically improves CD19-CAR T cell therapy against acute lymphoblastic leukemia

(A-C) Human peripheral blood mononuclear cells (PBMC) were activated with anti-CD3/CD28 beads for 5 days and then cultured another 4 days before phenotypic analyses. The cells were exposed to DMSO or UK5099 (MPCi) during the entire time of culture. Percentage CD62L-positive CD4$^+$ T cells (A), mean fluorescent intensity of CD62L in the CD62L-positive population (B), the percentage of stem cell-like memory

$CD4^+$ T cells (C) was measured by flow cytometry. (n = 7 human donors/group, pooled data from 2 independent experiments)

(D) Transduction efficiency of human T cells activated and cultured for 7 days in the presence of DMSO or MPCi, assessed by Protein-L measurement by flow cytometry.

(E) The body weight of NOD scid γ (NSG) mice bearing advanced systemic NALM6 leukemia xenografts left either untreated, or treated with non-transduced (NTD) or anti-CD19 CAR (CAR) T cells conditioned with DMSO or UK5099 (MPCi). (n = 4 mice for untreated, n=5 mice for NTD DMSO and NTD MPCi, n = 7 mice for CAR DMSO and n = 8 mice for CAR MPCi, pooled data from 2 independent experiments)

(F-H) Number of $CD4^+$ T cells (F) or $CD8^+$ T cells (G) per μl of blood in NSG mice treated with MPCi-conditioned anti-CD19 CAR T cells. Flow cytometry analysis was performed on blood samples collected at the indicated number of days following adoptive cell transfer (ACT). Shown in (H) is a representative dot blot graph showing CD45RO and CD62L expression in $CD8^+$ T cells 27 days post-ACT, allowing the detection of effector memory (EM), central memory (CM) and stem cell-like memory (SCM) T cells. (n = 8 mice, pooled data from 2 independent experiments)

Data is represented as mean $\pm$ standard error of mean. Statistics are based on paired, two-tailed Students t-test (A-C), with $^*p < 0.05$, $^{**}p < 0.01$, $^{***}p < 0.001$.

Figure S1, related to Figure 2

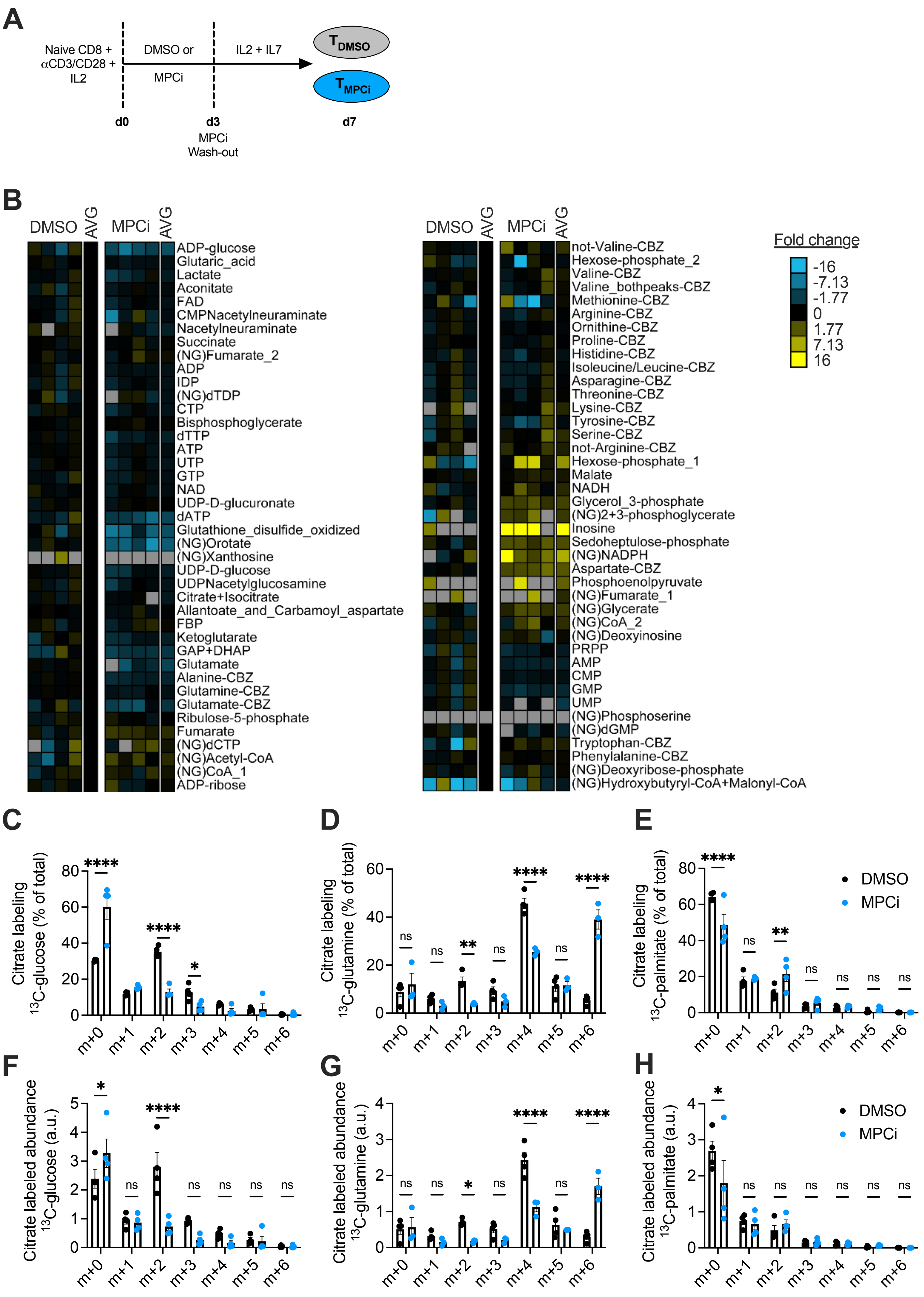

Figure S2, related to Figure 2

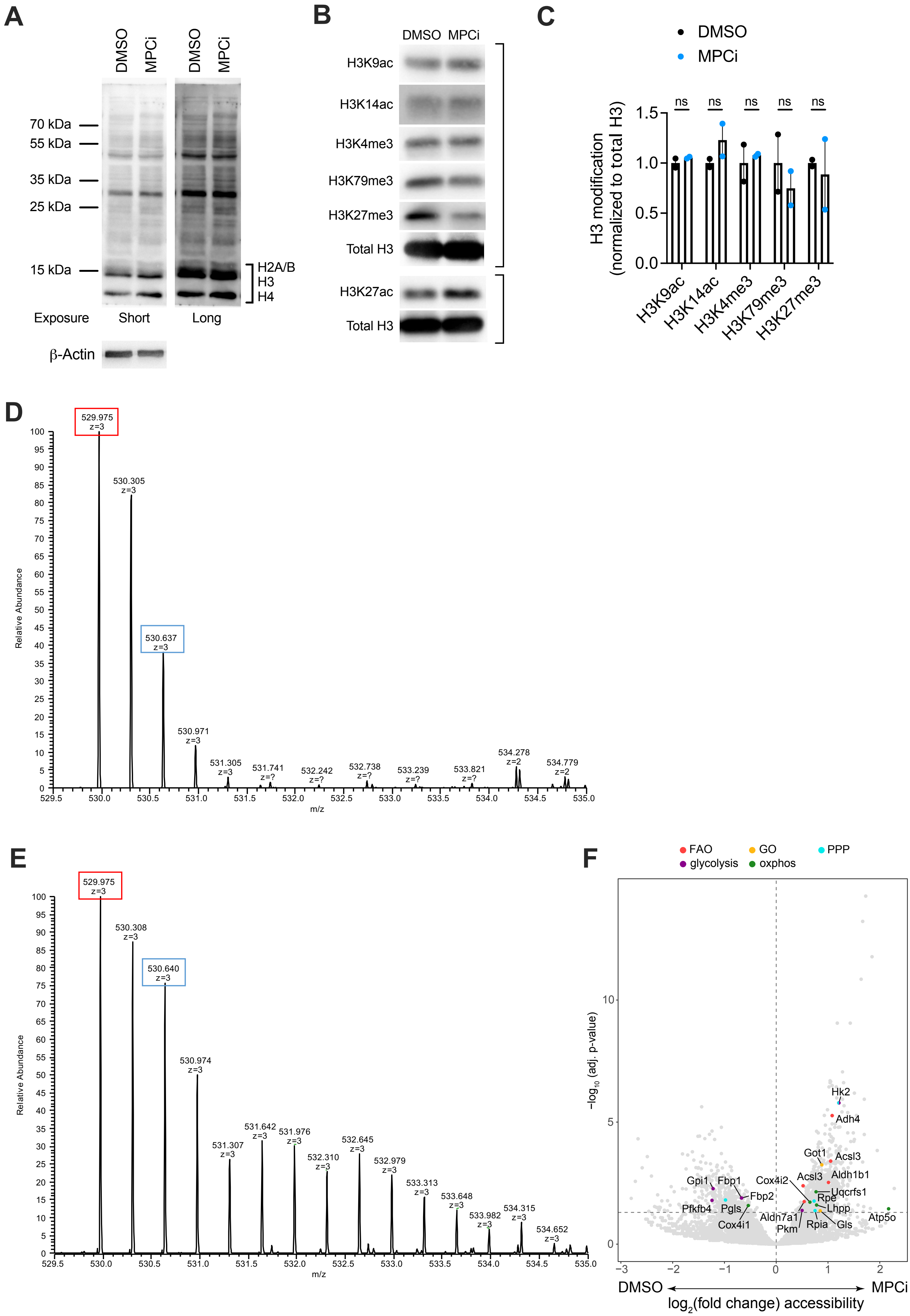

Figure S3, related to Figure 3

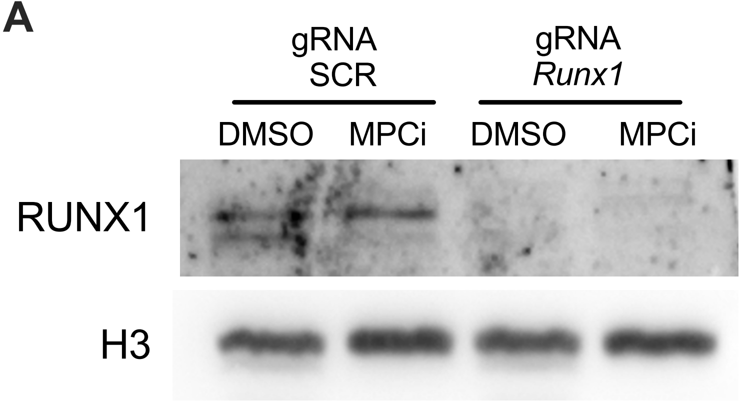

Figure S4, related to Figure 4

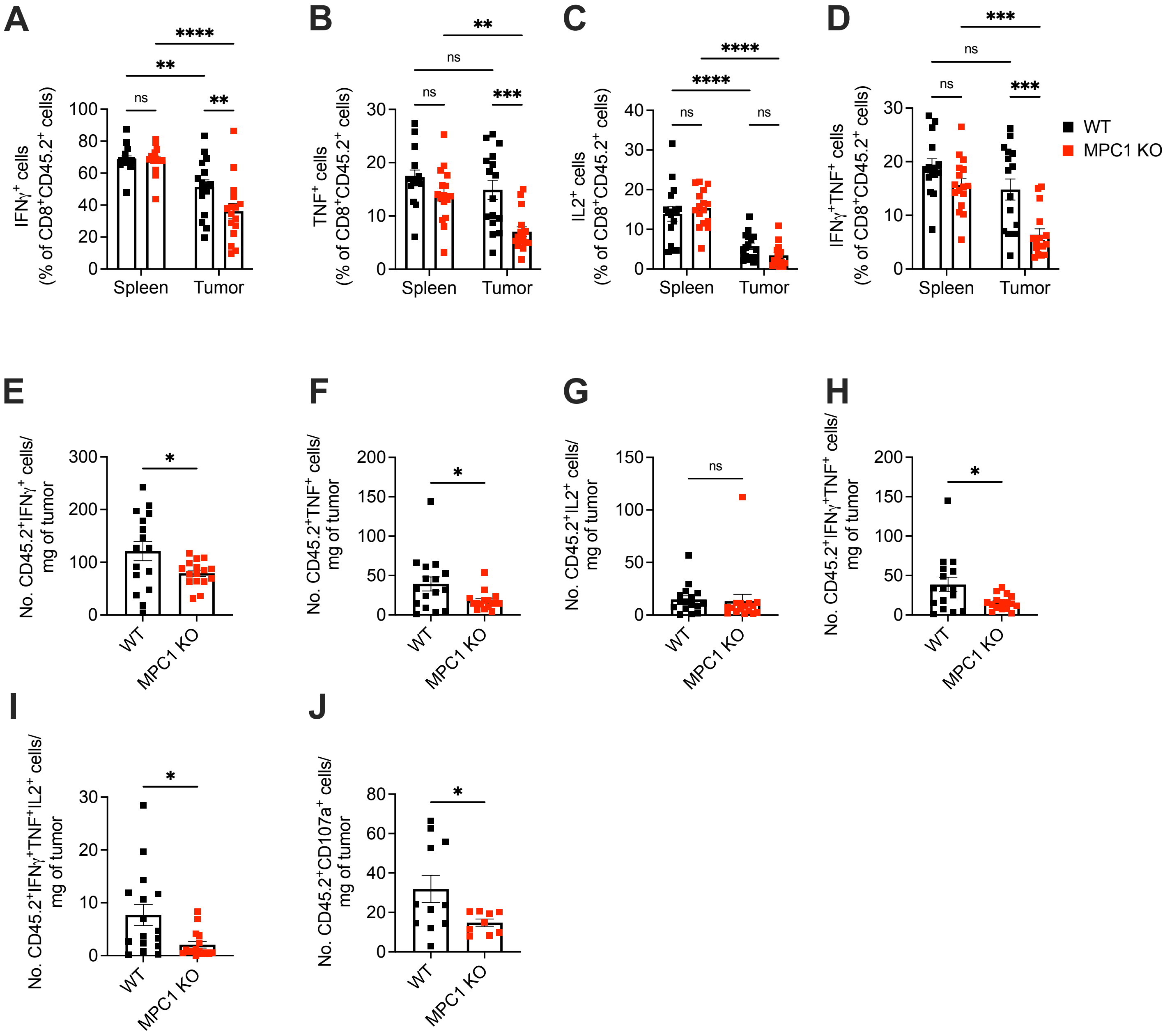

Figure S5, related to Figure 5

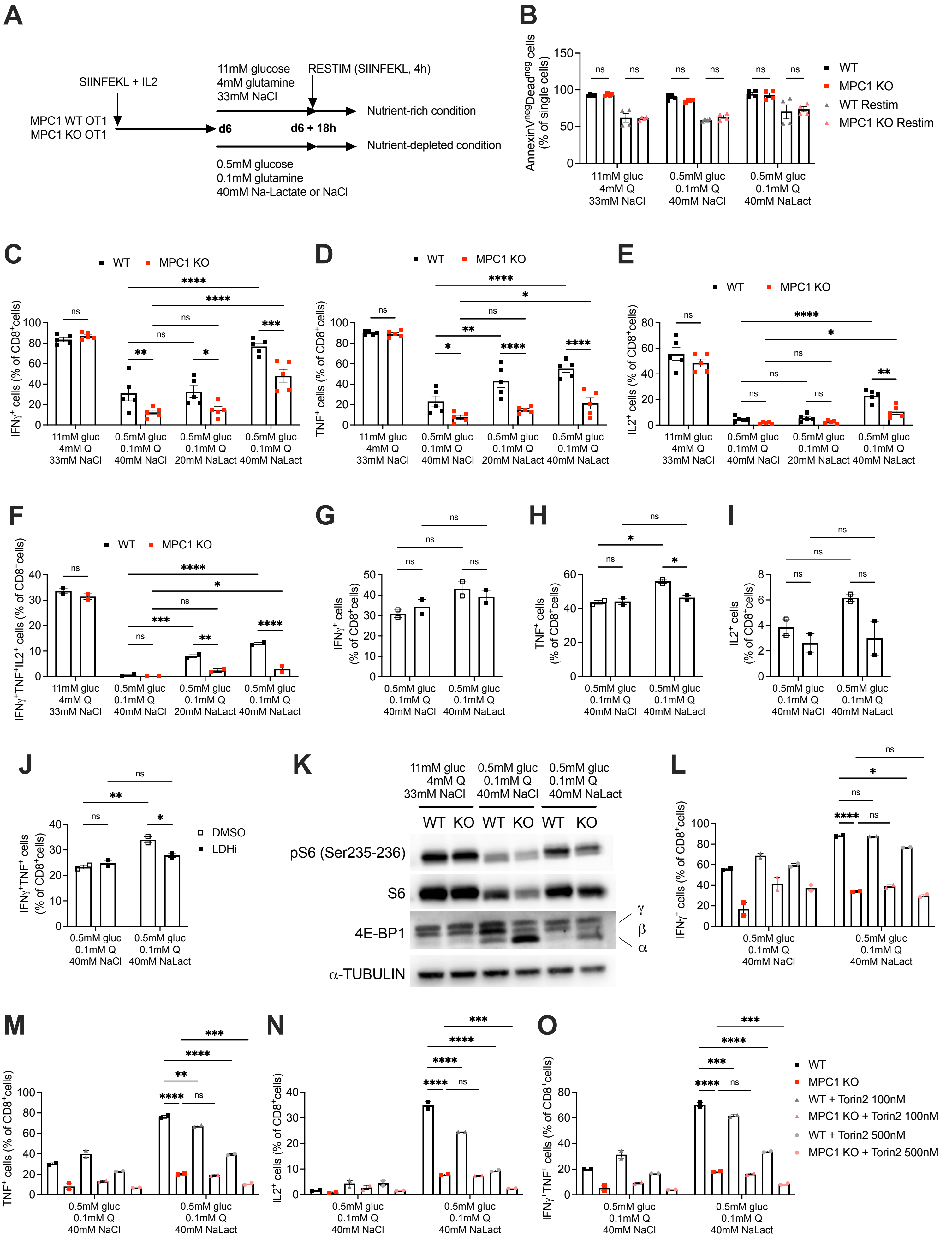

Figure S6, related to Figure 6

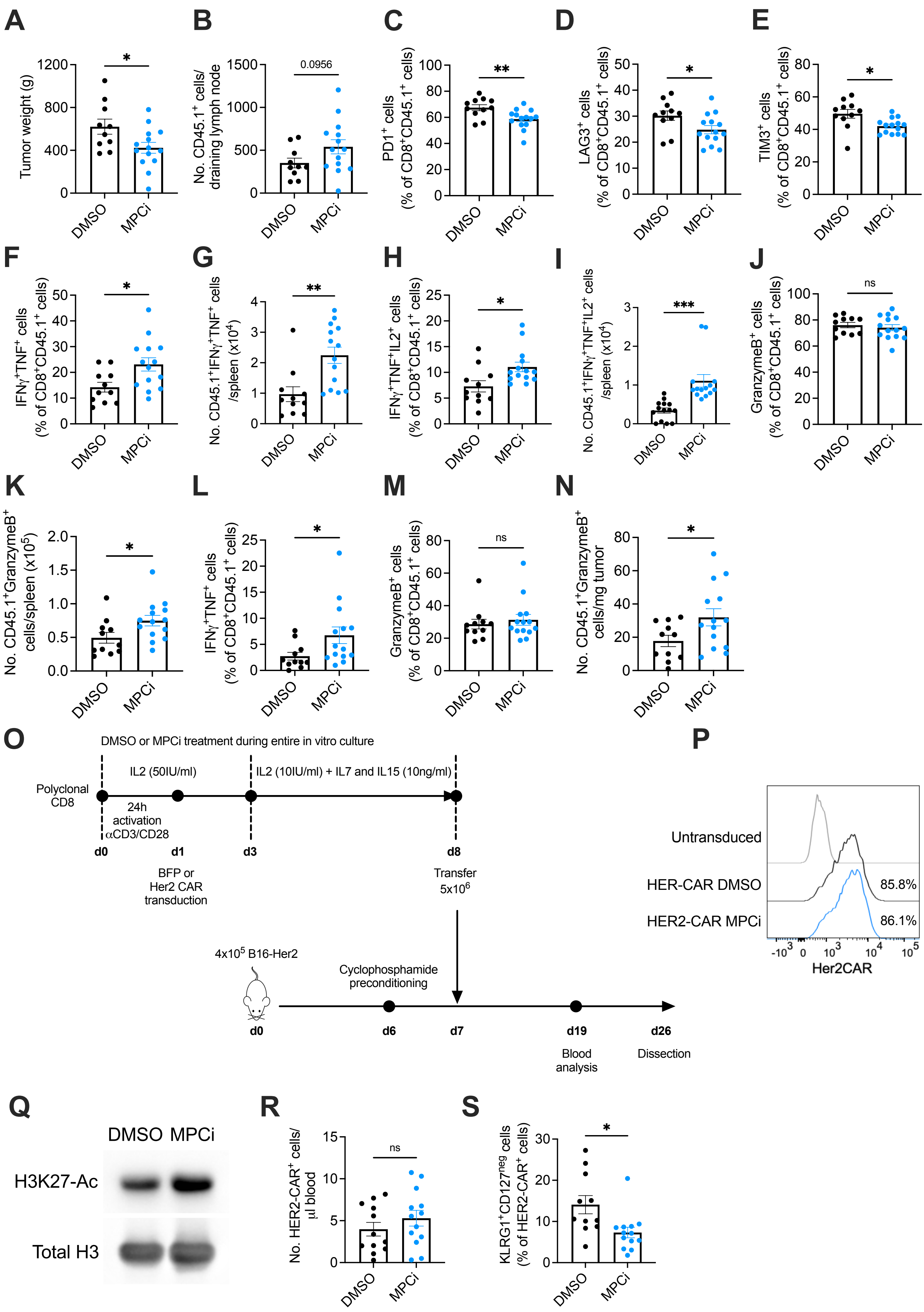

Figure S7, related to Figure 7

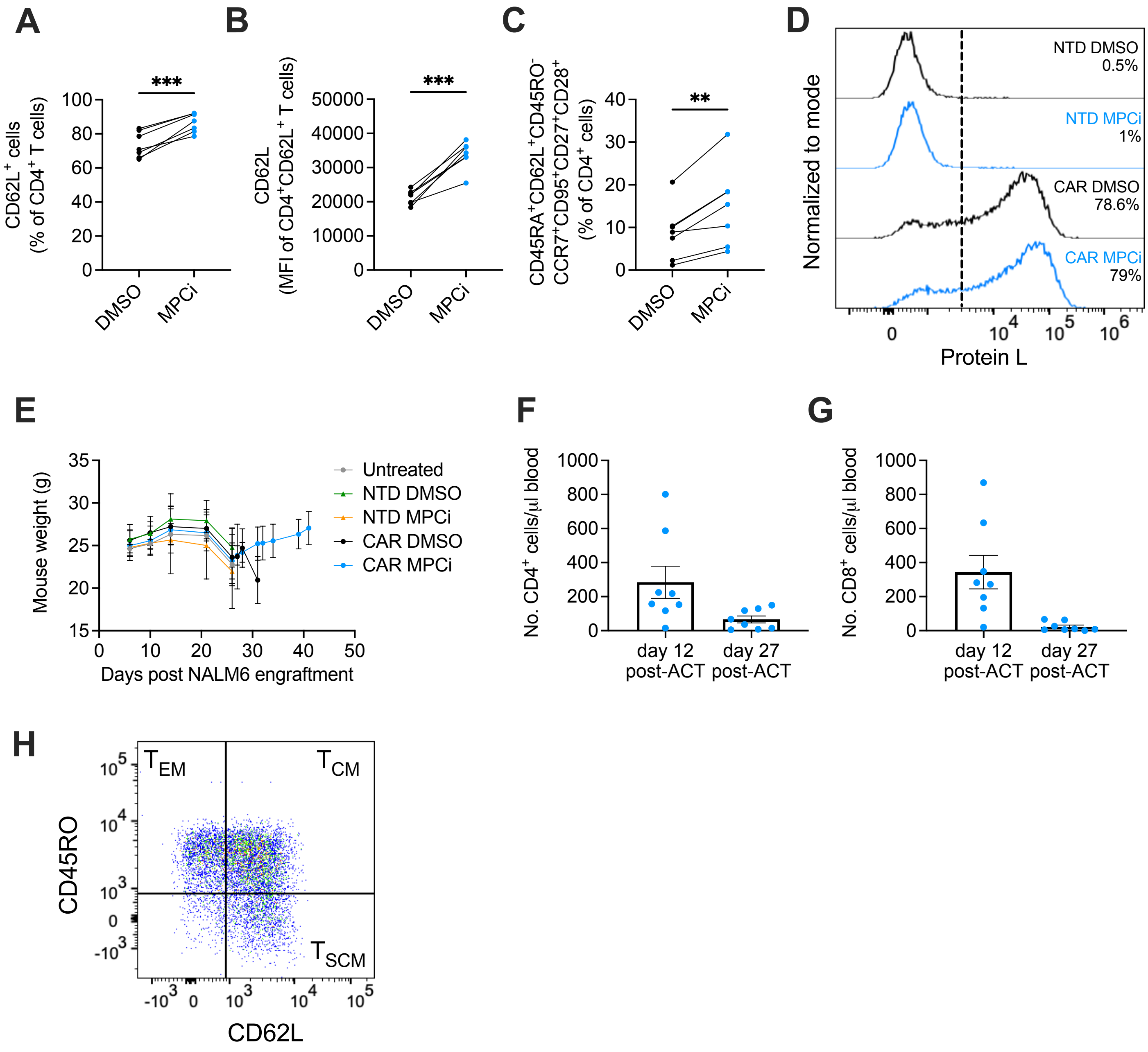

Supplement: Document S1. Figures S1–S7 [file mmc1.pdf]
