# Supplementary material for: The mitochondrial pyruvate carrier regulates memory T cell differentiation and antitumor function
Source: Cell Metab. 2022 May 3;34(5):731–746.e9. doi: 10.1016/j.cmet.2022.03.013 (PMC9116152; doi:10.1016/j.cmet.2022.03.013)

Figure 2G

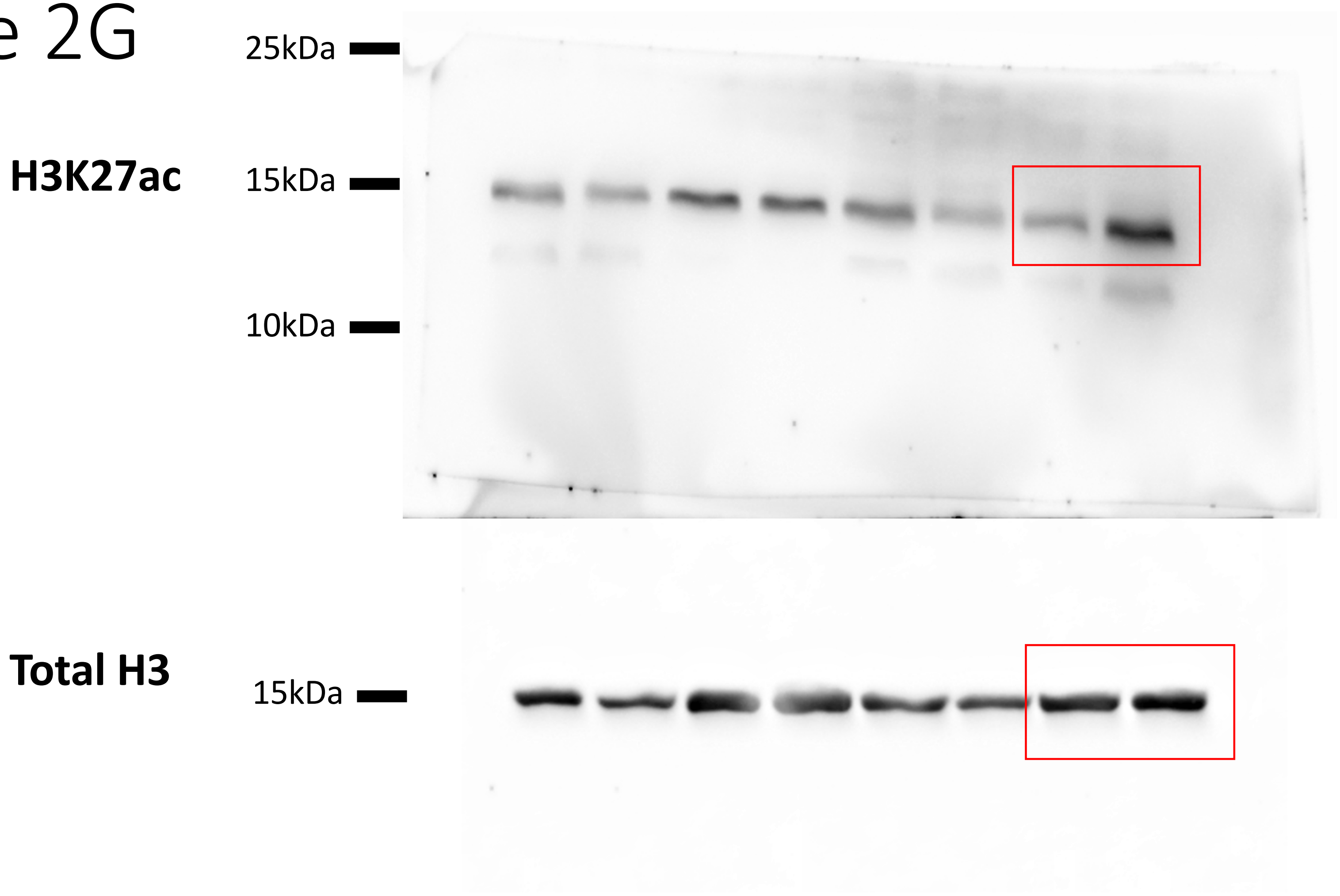

Figure 5K

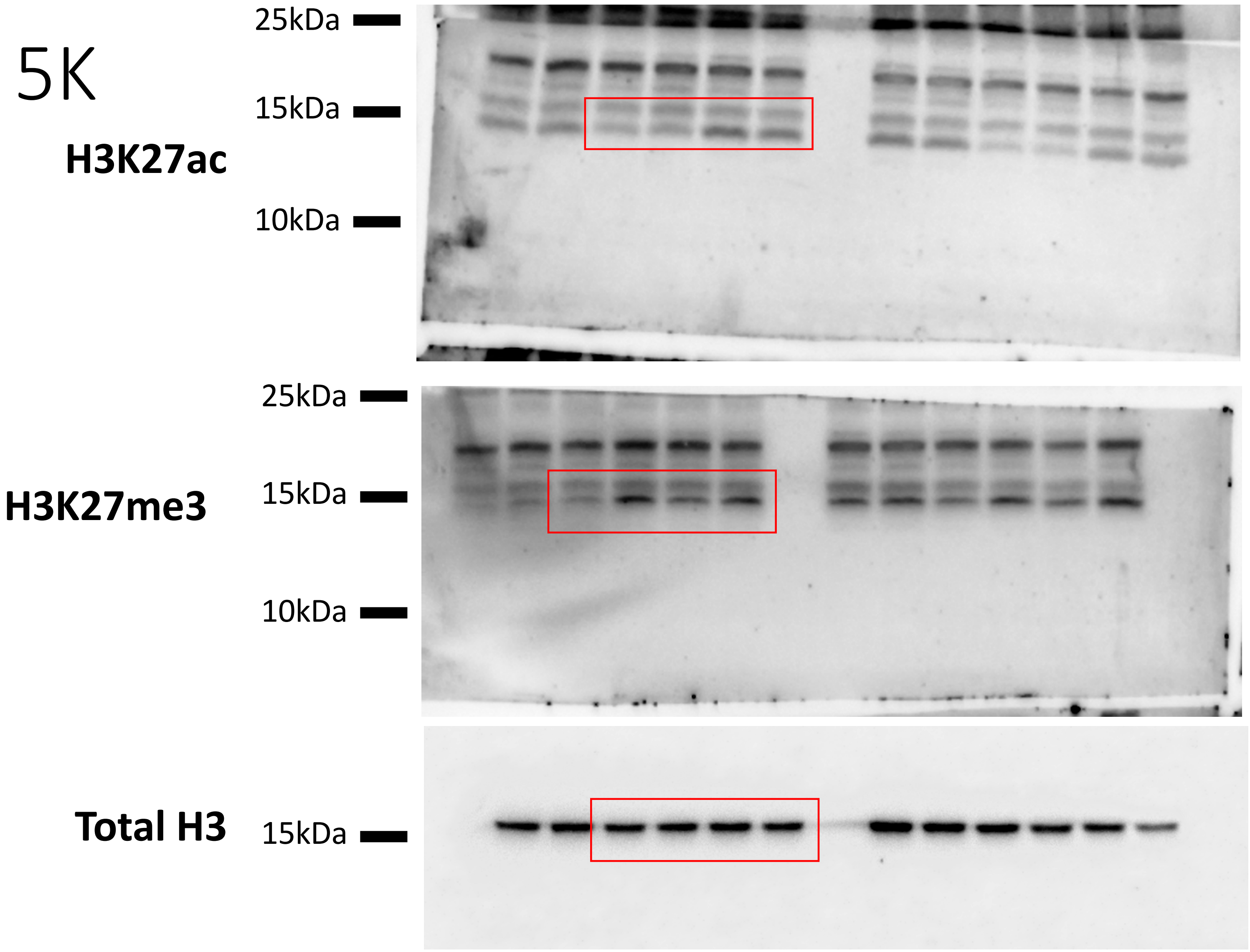

H3K27ac
25kDa
15kDa
10kDa
H3K27me3
25kDa
15kDa
10kDa
Total H3
15kDa

Figure S2A

Acetylated lysine
Short exposure

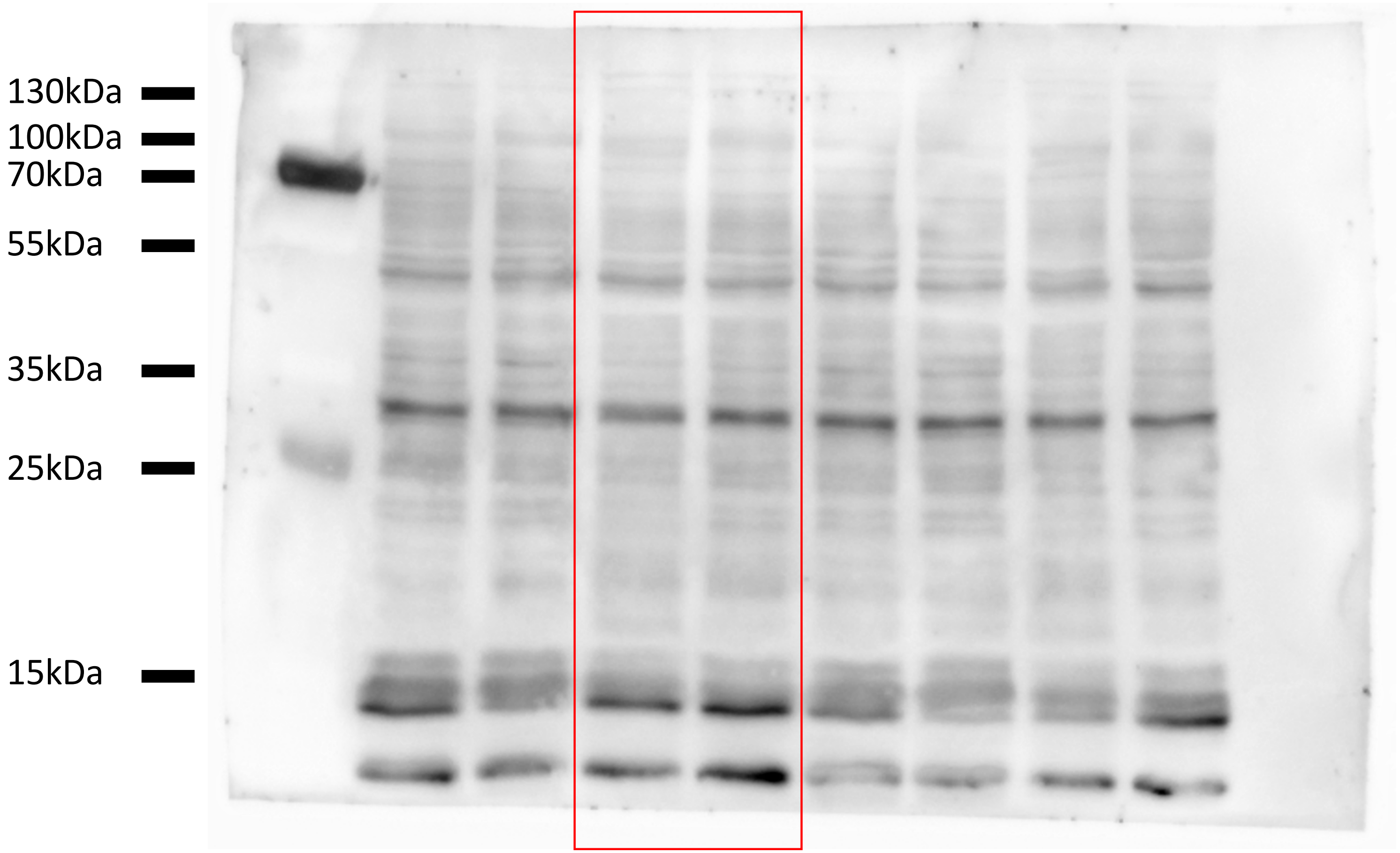

Figure S2A

Acetylated lysine
long exposure

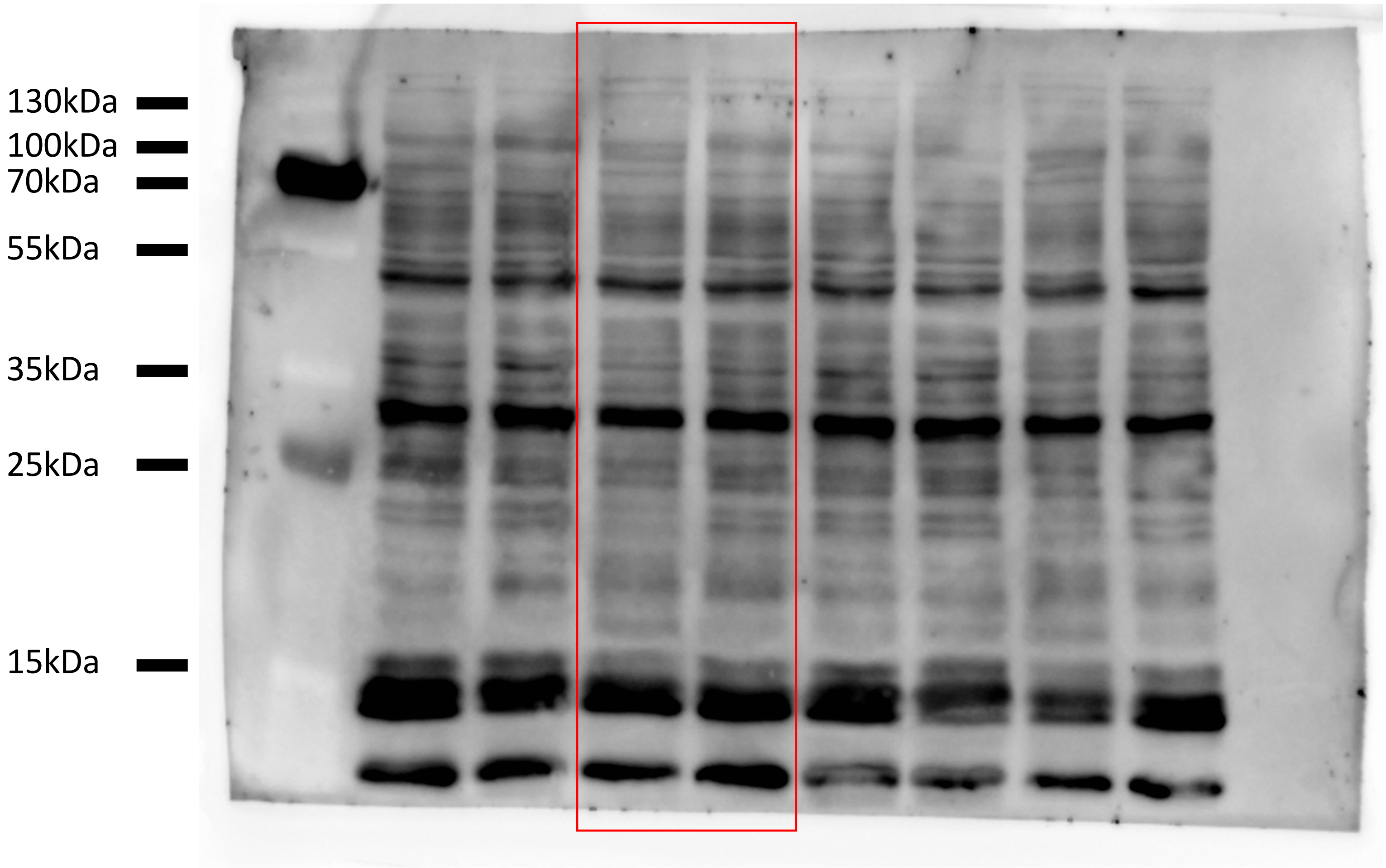

Figure S2A

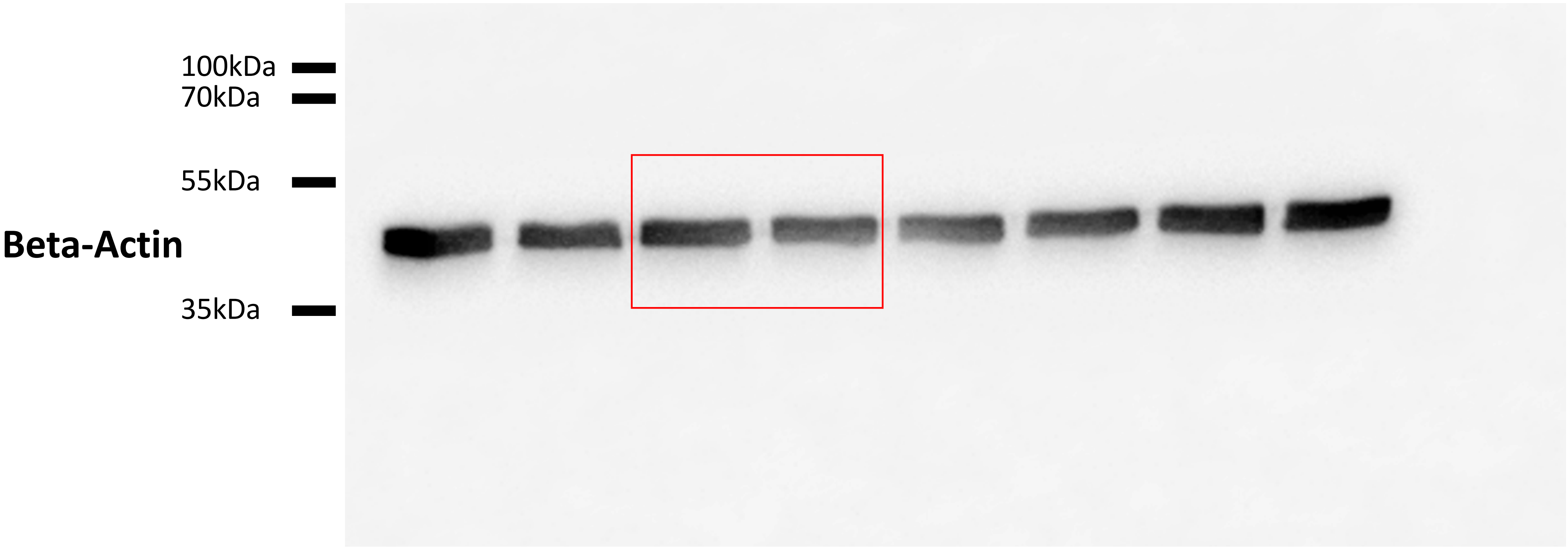

Figure S2B

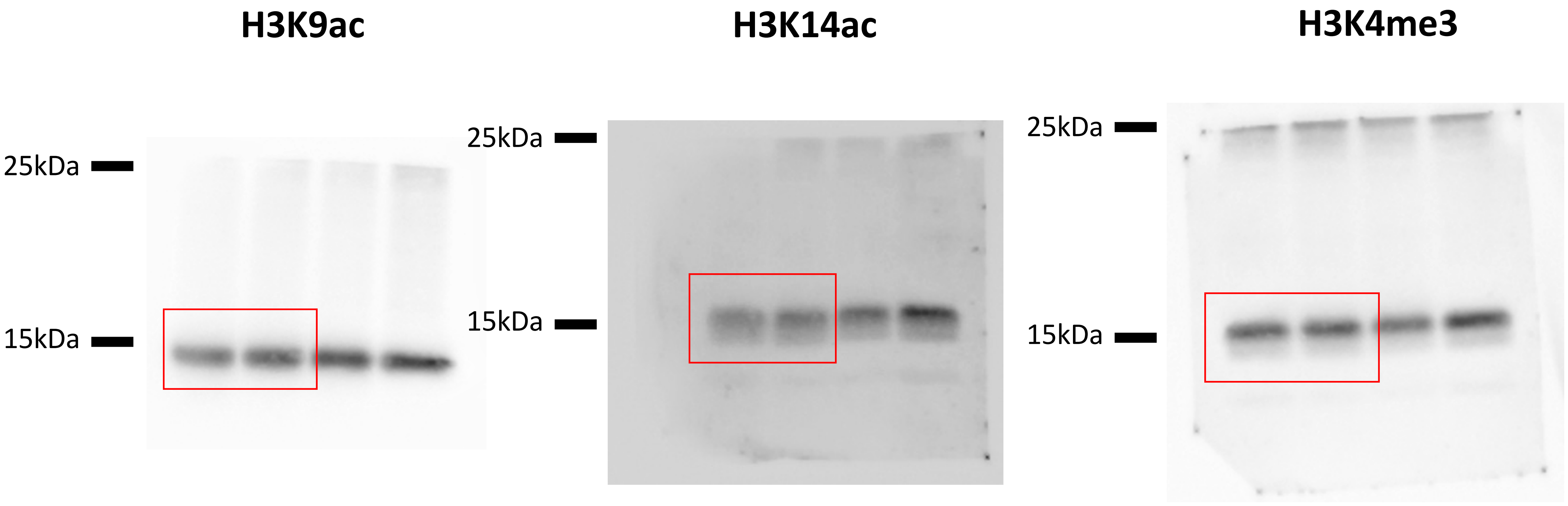
H3K9ac
H3K14ac
H3K4me3
25kDa
15kDa
25kDa
15kDa
25kDa
15kDa

Figure S2B

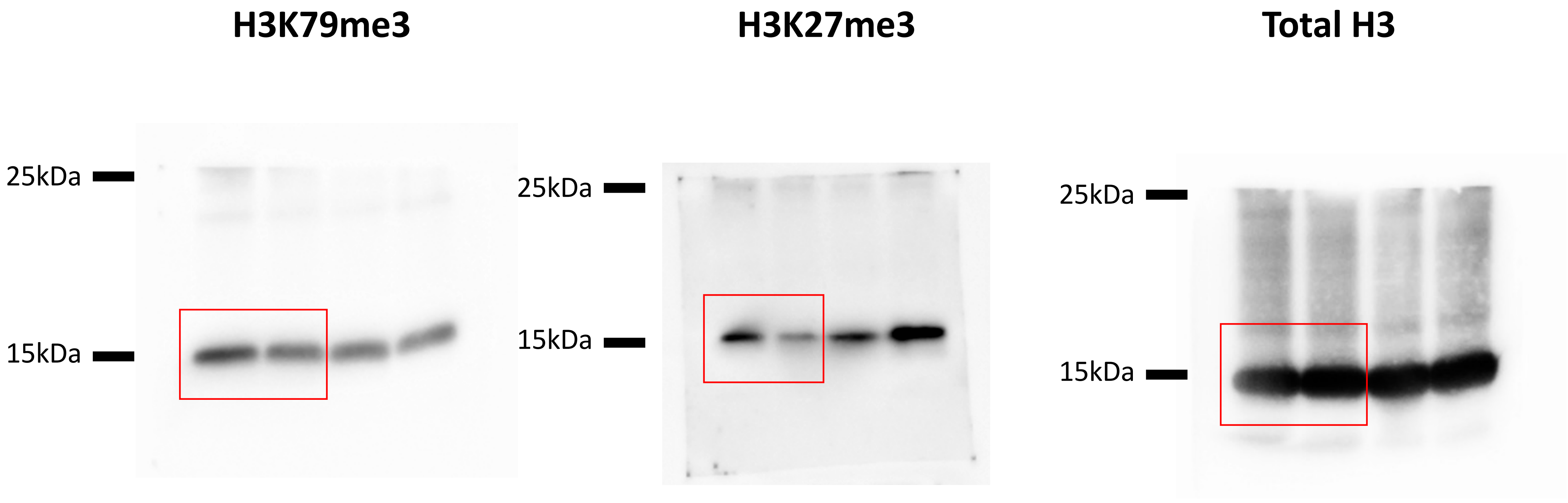
H3K79me3
H3K27me3
Total H3
25kDa
15kDa
25kDa
15kDa
25kDa
15kDa

Figure S2B

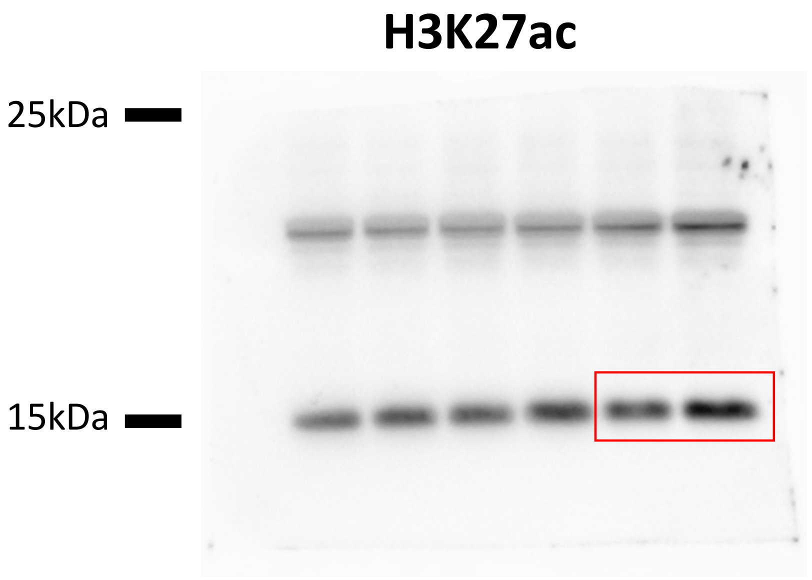

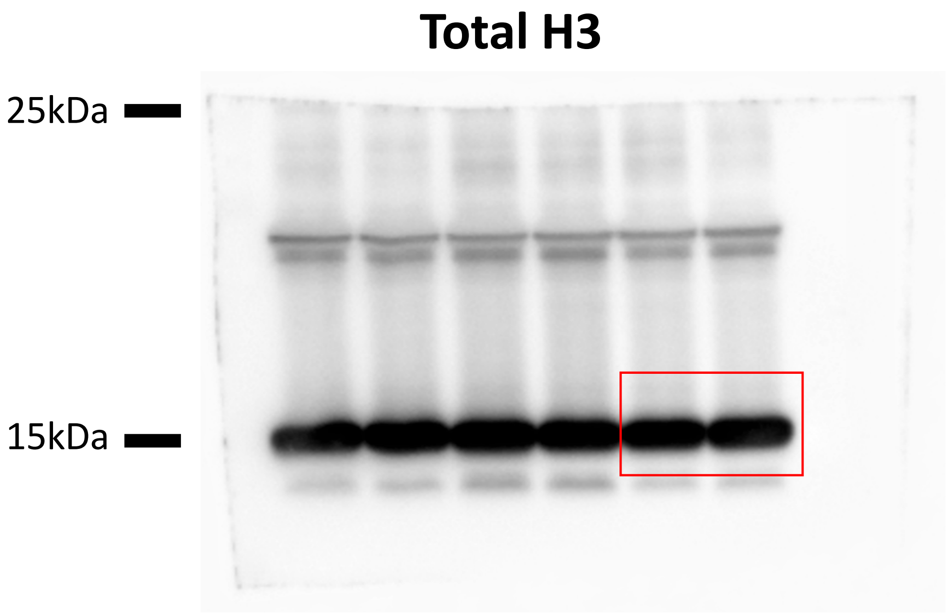

Figure S3A

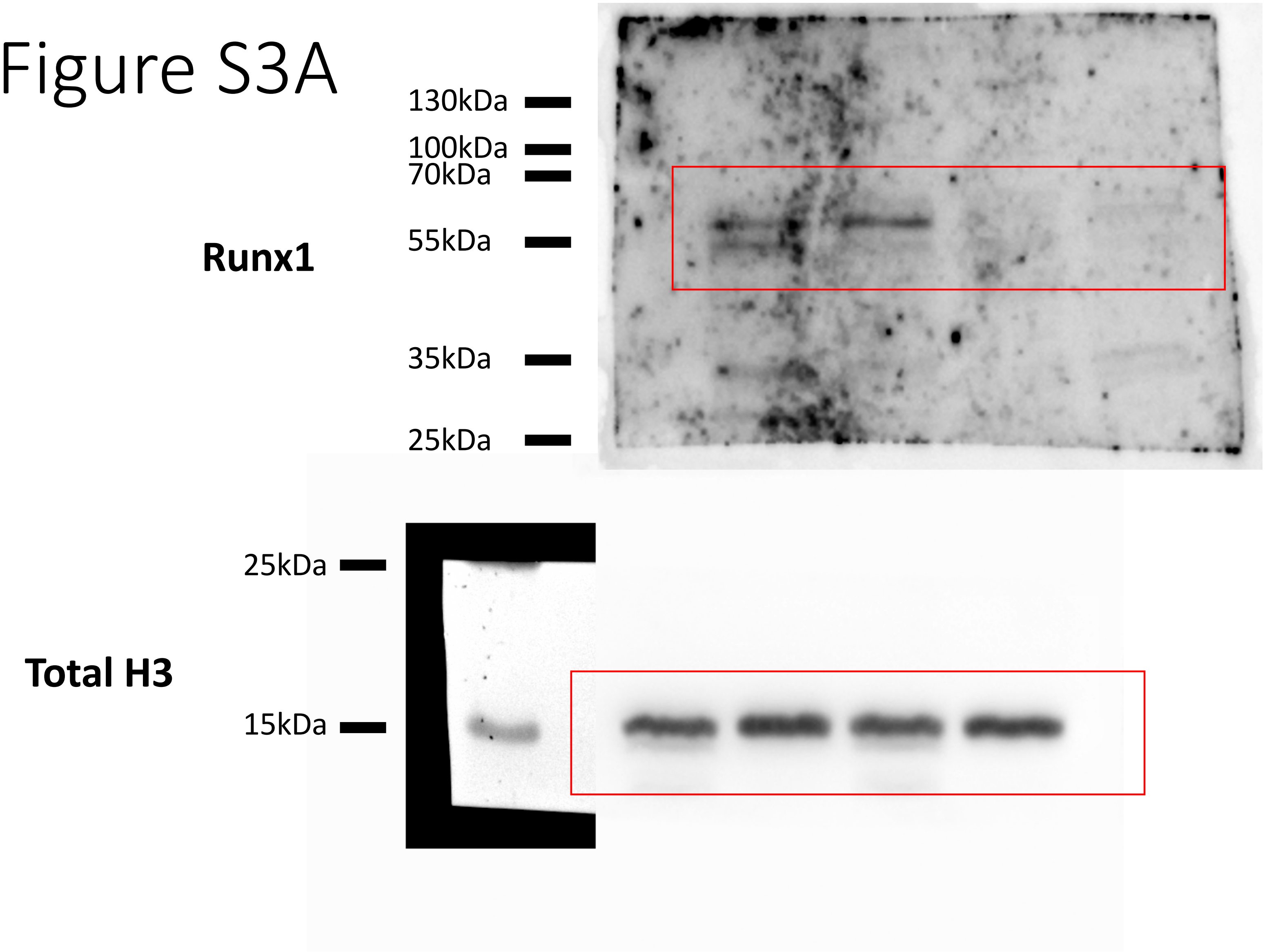
Runx1
130kDa
100kDa
70kDa
55kDa
35kDa
25kDa
Total H3
25kDa
15kDa

Figure S5K

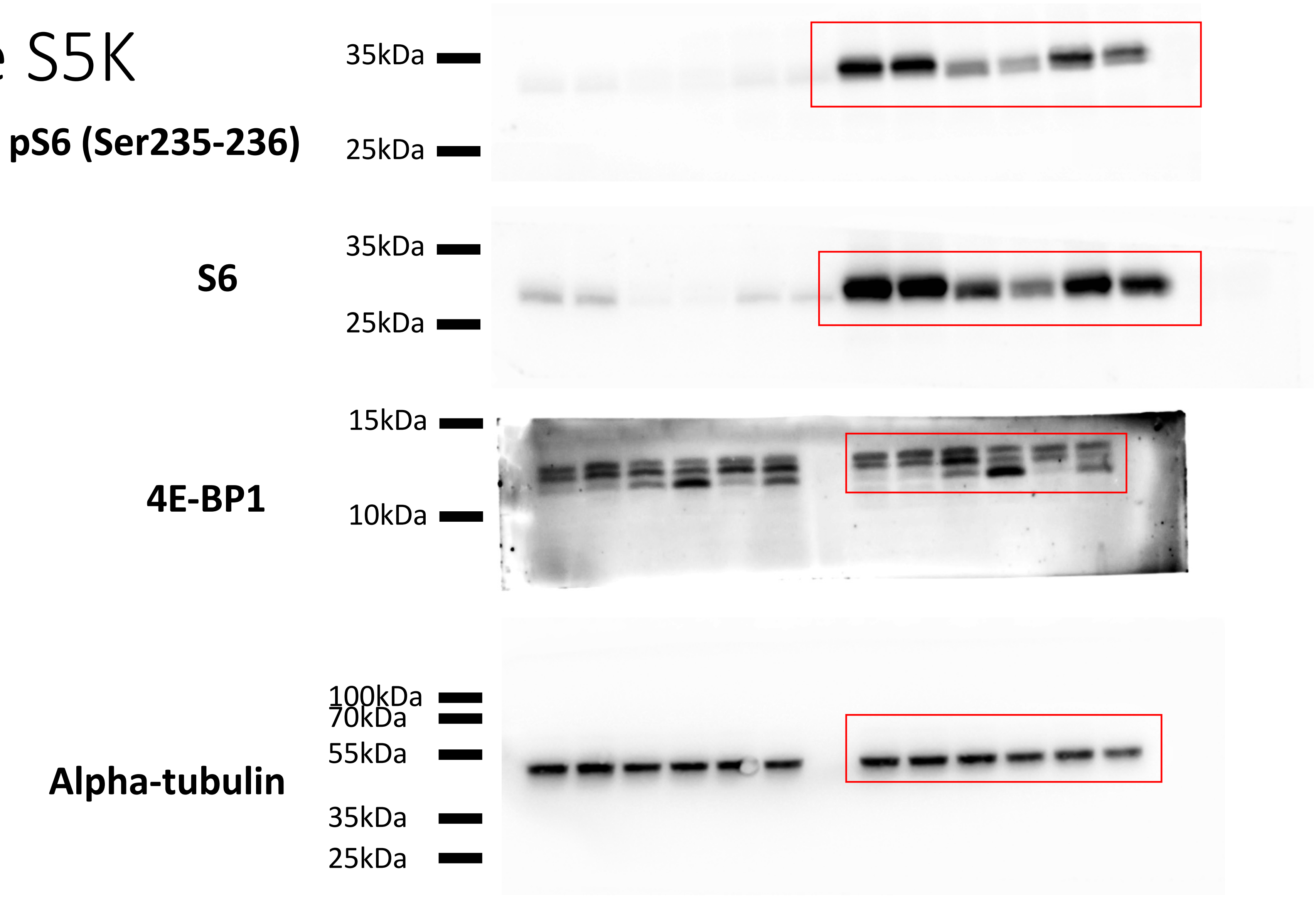

pS6 (Ser235-236)
35kDa
25kDa
S6
35kDa
25kDa
4E-BP1
15kDa
10kDa
Alpha-tubulin
100kDa
70kDa
55kDa
35kDa
25kDa

Figure S6Q

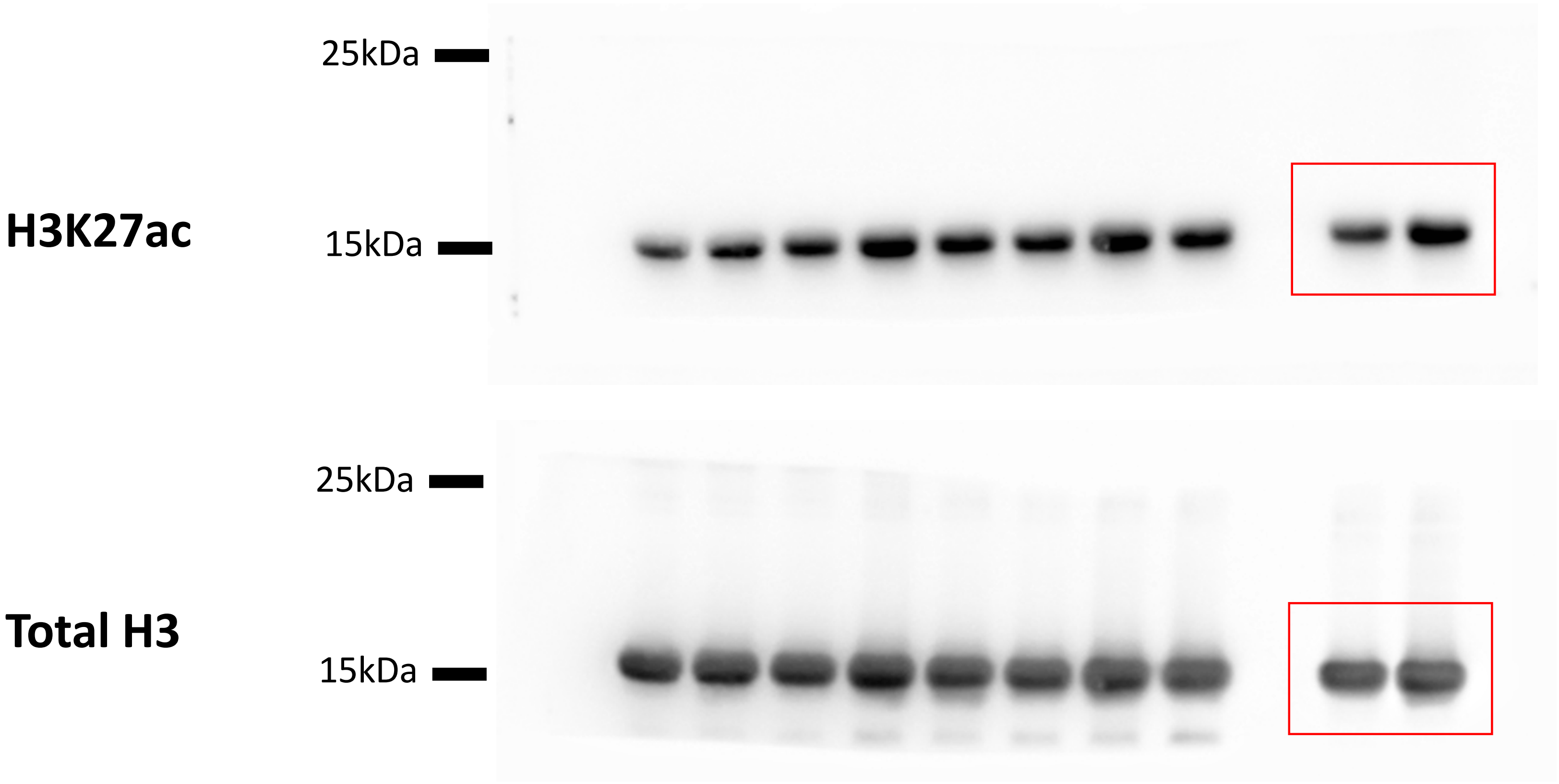

Supplement: Data S1. Uncropped western blot images and unprocessed data — underlying the display items in the manuscript, related to Figures 1–7 and S1–S7 [file mmc3.zip › Data S1 uncropped blot.pdf]
