# Supplementary material for: The mitochondrial pyruvate carrier regulates memory T cell differentiation and antitumor function
Source: Cell Metab. 2022 May 3;34(5):731–746.e9. doi: 10.1016/j.cmet.2022.03.013 (PMC9116152; doi:10.1016/j.cmet.2022.03.013)

Cell Metabolism

Article

The mitochondrial pyruvate carrier regulates memory T cell differentiation and antitumor function

Graphical abstract

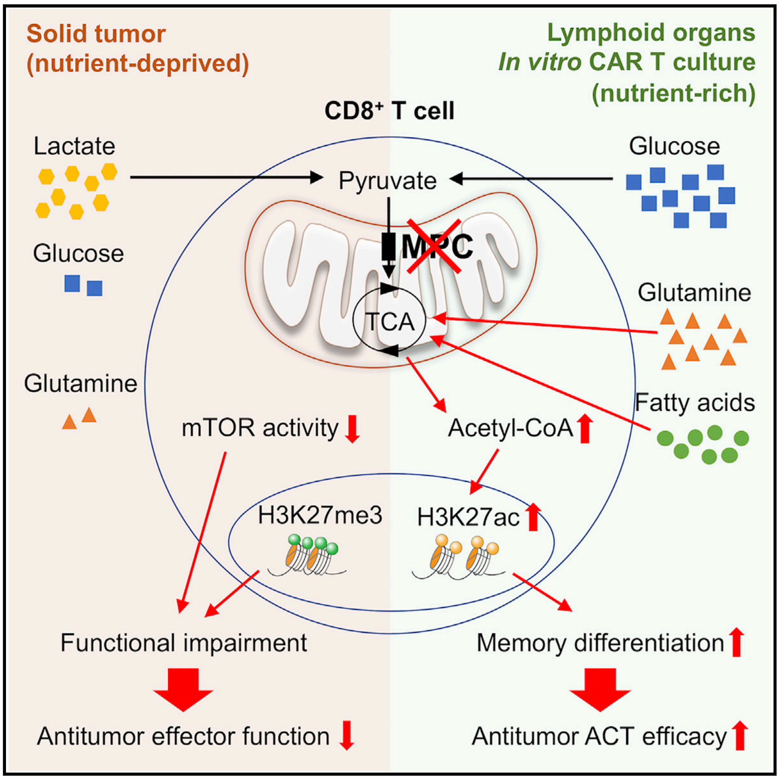

Highlights

- Genetic and pharmacological MPC inhibition promotes memory T cell differentiation
- A metabolic-epigenetic axis enables memory T cell formation upon MPC inhibition
- Transient MPC blockade during CAR T cell manufacturing enhances antitumor efficacy
- The MPC allows lactate oxidation to sustain antitumor function of cytotoxic T cells

Authors

Mathias Wenes, Alison Jaccard, Tania Wyss, ..., Sophia Y. Lunt, Ping-Chih Ho, Pedro Romero

Correspondence

mathias.wenes@unige.ch (M.W.),
pedro.romero@unil.ch (P.R.)

In brief

Wenes et al. show that lactate oxidation via pyruvate conversion and mitochondrial import may sustain antitumor function of cytotoxic T cells in the tumor microenvironment. In contrast, short-term inhibition of the mitochondrial pyruvate carrier in recently activated T cells promotes memory differentiation, which enhances antitumor activity upon adoptive transfer therapy.

Wenes et al., 2022, Cell Metabolism *34*, 731–746
May 3, 2022 © 2022 The Author(s). Published by Elsevier Inc.
https://doi.org/10.1016/j.cmet.2022.03.013

CellPress

Cell Metabolism

CellPress
OPEN ACCESS

Article

The mitochondrial pyruvate carrier regulates memory T cell differentiation and antitumor function

Mathias Wenes,[1,13,*] Alison Jaccard,[1,2] Tania Wyss,[1] Noelia Maldonado-Pérez,[3] Shao Thing Teoh,[4] Anouk Lepez,[5,6,7] Fabrice Renaud,[1] Fabien Franco,[1,2] Patrice Waridel,[8] Céline Yacoub Maroun,[1] Benjamin Tschumi,[1] Nina Dumauthioz,[1] Lianjun Zhang,[9,10] Alena Donda,[1] Francisco Martín,[3] Denis Migliorini,[5,6,7,11] Sophia Y. Lunt,[4,12] Ping-Chih Ho,[1,2] and Pedro Romero[1,*]

[1]Department of Oncology, University of Lausanne, Épalinges, Switzerland
[2]Ludwig Institute for Cancer Research, University of Lausanne, Épalinges, Switzerland
[3]Gene and Cell Therapy Unit, Genomic Medicine Department, Pfizer-University of Granada-Junta de Andalucía, Centre for Genomics and Oncological Research (GENYO), Granada, Spain
[4]Department of Biochemistry and Molecular Biology, Michigan State University, East Lansing, MI, USA
[5]Brain Tumor and Immune Cell Engineering Group, Faculty of Medicine, University of Geneva, Geneva, Switzerland
[6]Center for Translational Research in Onco-Hematology, University of Geneva, Geneva, Switzerland
[7]Swiss Cancer Center Léman, Geneva and Lausanne, Switzerland
[8]Protein Analysis Facility, University of Lausanne, Lausanne, Switzerland
[9]Institute of Systems Medicine, Chinese Academy of Medical Sciences & Peking Union Medical College, Beijing 100005, China
[10]Suzhou Institute of Systems Medicine, Suzhou 215123, China
[11]Department of Oncology, Geneva University Hospitals (HUG), Geneva, Switzerland
[12]Department of Chemical Engineering and Materials Science, Michigan State University, East Lansing, MI, USA
[13]Lead contact
*Correspondence: mathias.wenes@unige.ch (M.W.), pedro.romero@unil.ch (P.R.)
https://doi.org/10.1016/j.cmet.2022.03.013

SUMMARY

Glycolysis, including both lactate fermentation and pyruvate oxidation, orchestrates CD8+ T cell differentiation. However, how mitochondrial pyruvate metabolism and uptake controlled by the mitochondrial pyruvate carrier (MPC) impact T cell function and fate remains elusive. We found that genetic deletion of MPC drives CD8+ T cell differentiation toward a memory phenotype. Metabolic flexibility induced by MPC inhibition facilitated acetyl-coenzyme-A production by glutamine and fatty acid oxidation that results in enhanced histone acetylation and chromatin accessibility on pro-memory genes. However, in the tumor microenvironment, MPC is essential for sustaining lactate oxidation to support CD8+ T cell antitumor function. We further revealed that chimeric antigen receptor (CAR) T cell manufacturing with an MPC inhibitor imprinted a memory phenotype and demonstrated that infusing MPC inhibitor-conditioned CAR T cells resulted in superior and long-lasting antitumor activity. Altogether, we uncover that mitochondrial pyruvate uptake instructs metabolic flexibility for guiding T cell differentiation and antitumor responses.

INTRODUCTION

CD8+ T cells are crucial mediators of adaptive immune responses against cancer cells and pathogens. Upon antigen stimulation, naive CD8+ T cells undergo extensive clonal expansion and differentiation into effector cells. A major proportion of these cells are short-lived effector cells (SLECs) that are mostly terminally differentiated and characterized by a potent cytotoxic potential. The remaining cells are memory precursor effector cells (MPECs) that further differentiate into long-lived, self-renewing memory CD8+ T cells (Joshi et al., 2007). Self-renewal and pluripotency are capacities that characterize the ideal immune cell, which is fit for adoptive cell transfer (ACT) immunotherapy against cancer and able to overcome some of the current issues with ACT (Gattinoni et al., 2012). Indeed, despite showing promising results in a fraction of patients, T lymphocytes prepared for ACT are generally terminally differentiated, resulting in inefficient engraftment and cancer recurrence. It has been shown that the infusion of T cells with a self-renewing, memory phenotype confers a stronger and more sustained antitumor response (Chen et al., 2021a; Fraietta et al., 2018; Klebanoff et al., 2011).

A multitude of molecular pathways, transcription factors, and epigenetic imprinting have been shown to drive effector versus memory CD8+ T cell differentiation (Gray et al., 2017; Pais Ferreira et al., 2020; Yu et al., 2017). It has become clear that those processes are closely intertwined with the cellular metabolic state. The energetic and biosynthetic requirements of clonally expanding effector cells versus resting, long-lived memory

This is an open access article under the CC BY license (http://creativecommons.org/licenses/by/4.0/).

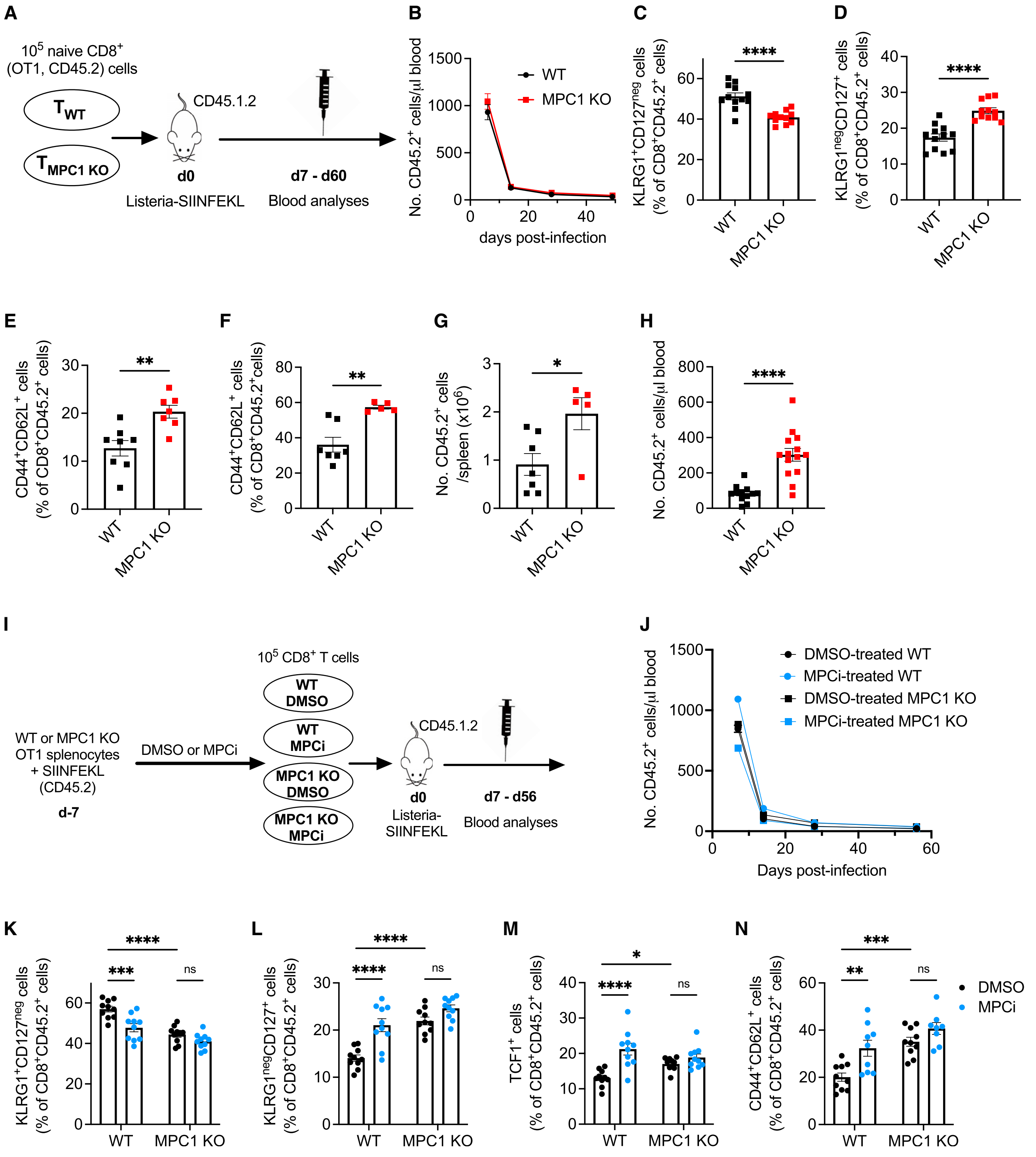

Figure 1. Inhibiting mitochondrial pyruvate import during $CD8^+$ T cell activation favors memory differentiation

(A) *Listeria* experiment.

(B) Number of transferred cells in the blood.

(C–E) Percentage of SLECs (C) and MPECs (D) at 1 week and T_{CM} cells (E) at 4 weeks post-infection, out of transferred cells in the blood. n = 11–12 mice/group in (B)–(D) or 7–8 mice/group in (E); pooled data from 2 independent experiments.

(F and G) Percentage of T_{CM} cells (F) and number of transferred cells (G) in the spleen 60 days post-infection (F) (n = 7 mice [WT] versus 5 mice [MPC1 KO]; pooled data from 2 independent experiments).

(H) At 60 days post-infection, transferred cells were FACS-sorted from spleens and retransferred in new host, followed by *Listeria* infection. Expansion of the retransferred cells was measured in the blood at day 6 post-infection (n = 12–14 mice/group; pooled data from 2 independent experiments).

(legend continued on next page)

CD8+ T cells are of a different magnitude and rely on distinct metabolic pathways (Cui et al., 2015; O'Sullivan et al., 2014; Wang et al., 2011). Interestingly, metabolically demanding effector CD8+ T cells split glucose-derived pyruvate utilization both over lactate fermentation and mitochondrial oxidation (Ma et al., 2019). At the crossroads of those opposite metabolic fates, stands the mitochondrial pyruvate carrier (MPC), which is a heterodimer in the inner mitochondrial membrane consisting of an MPC1 and MPC2 protein and the sole entry point of pyruvate into the mitochondria (Bricker et al., 2012; Herzig et al., 2012). Although mitochondrial pyruvate import has been shown to be crucial during thymic development of T cell precursors (Ramstead et al., 2020), its role in mature T cell response and effector versus memory T cell differentiation remains unknown.

Here, we show that genetic deletion of MPC does not affect effector function but skews CD8+ T cell differentiation toward a memory phenotype. Metabolic flexibility induced by MPC inhibition allowed for a metabolic-epigenetic crosstalk and memory differentiation orchestrated by RUNX1. However, MPC deletion in a nutrient-deprived tumor microenvironment blunted CD8+ T cell effector function due to an inability of oxidizing lactate in the mitochondria. In several ACT immunotherapy models, this intra-tumoral defect could be circumvented by imprinting a memory phenotype with a small molecule MPC inhibitor (MPCi) during chimeric antigen receptor (CAR) T cell *in vitro* expansion to infuse wild-type (WT) T cells with superior and long-lasting antitumor activity.

RESULTS

Genetic or pharmacological interference of mitochondrial pyruvate import during CD8+ T cell activation favors memory differentiation

To investigate the role of mitochondrial pyruvate import in the CD8+ T cell response to acute infection, we crossed *Mpc1*$^{fl/fl}$ mice with mice expressing CRE recombinase under the control of the *Cd4* promoter on an OT1 background, allowing for antigen-specific response against the SIINFEKL peptide of ovalbumin. Naive CD45.2+ CD8+ T cells from OT1 *Mpc1*$^{fl/fl}$ (referred to as WT) and *Mpc1*$^{fl/fl}$ *Cd4-Cre* (referred to MPC1 KO) mice were transferred into CD45.1.2+ mice, followed by infection with *Listeria monocytogenes* overexpressing SIINFEKL (Figure 1A). The kinetics of the CD8+ T cell response was not altered upon MPC1 deletion (Figure 1B). However, at day 7 post-infection, MPC1 KO T cells formed fewer SLECs and more MPECs (Figures 1C and 1D) as well as more central memory T (T_{CM}) cells 4 weeks after infection (Figure 1E). Ten weeks post-infection, MPC1 KO T cells were more abundant in the spleen and formed more T_{CM} cells (Figures 1F and 1G). To evaluate their functional potential, WT or MPC1 KO T cells from those spleens were re-transferred into new hosts, followed by *Listeria*-SIINFEKL infection. MPC1 KO T cells showed increased expansion kinetics, confirming their recall memory function (Figure 1H).

Because MPC1 deletion favors a memory precursor differentiation early after antigen encounter, we wondered if short-term interference with mitochondrial pyruvate import is sufficient to skew CD8+ T cell differentiation toward memory. We, therefore, activated WT splenocytes *in vitro* in the presence of 20 μM UK5099, a well-known MPCi (Halestrap, 1975), or DMSO as control. We also activated MPC1 KO splenocytes in the presence of MPCi to exclude potential off-target effects (Figure 1I). Upon transfer in mice and *Listeria* infection, short-term pharmacological intervention did not affect the kinetics (Figure 1J) but significantly skewed the CD8+ T cell response away from SLEC toward MPEC differentiation with more CD8+ T cells expressing the transcription factor TCF1 at day 7 post-infection and with more T_{CM} differentiation at day 56 post-infection (Figures 1K–1N). Importantly, the MPCi-induced memory-skewing features were of similar quality as genetic MPC1 deletion, and activating MPC1 KO T cells in the presence of MPCi did not further increase the memory differentiation (Figures 1K–1N). Thus, transient pharmacological inhibition of the MPC, in recently activated T cells, phenocopied the effects of its genetic ablation in T cells.

Together, these results indicate that interfering with mitochondrial pyruvate import during the activation and expansion of CD8+ T cells favors memory differentiation.

MPC inhibition in activated CD8+ T cells induces alternative metabolic fluxes that result in an epigenetic crosstalk and memory imprinting

Inhibiting mitochondrial pyruvate metabolism might result in important changes in metabolite levels in activated CD8+ T cells. Untargeted metabolomics analysis surprisingly did not reveal major changes in metabolite composition upon *in vitro* MPC inhibition (Figures S1A and S1B). The energy status of MPCi-treated CD8+ T cells remained unaltered as compared with DMSO (Figure 2A), altogether suggesting compensation by other metabolic substrates. It has been shown in several cell lines in culture that the inhibition of mitochondrial pyruvate import increases glutamine and fatty acid oxidation (FAO) (Vacanti et al., 2014; Yang et al., 2014). Using uniformly ^{13}C-labeled glucose, glutamine, or palmitate (Figure 2B), we could confirm that MPC inhibition suppresses glucose incorporation while increasing glutamine and palmitate incorporation into citrate (Figures 2C and S1C–S1H). Citrate is formed by the condensation of oxaloacetate with acetyl-CoA, whereas citrate itself can also serve as a source of extra-mitochondrial acetyl-CoA. MPC inhibition also reduced the incorporation of glucose into acetyl-CoA and increased that of glutamine (Figure 2D). Of note, only fully labeled (m+6) citrate, but not m+5, was increased upon MPC inhibition, indicating that an oxidative metabolism of glutamine, but not reductive carboxylation, contributed to acetyl-CoA

(I) Experimental scheme (MPCi = 20 μM UK5099).

(J) Number of transferred cells in the blood.

(K–N) Percentage of SLECs (K), MPECs (L), and TCF1-positive cells (M) at 1 week and T_{CM} cells (N) at 8 weeks post-infection, out of transferred cells in the blood (n = 10 mice/group; pooled data from 2 different experiments).

Data are represented as mean ± SEM. Statistics are based on unpaired, two-tailed Student's t test (C–H) or two-way ANOVA (K–N), *p < 0.05, **p < 0.01, ***p < 0.001, ****p < 0.0001, and ns (p > 0.05).

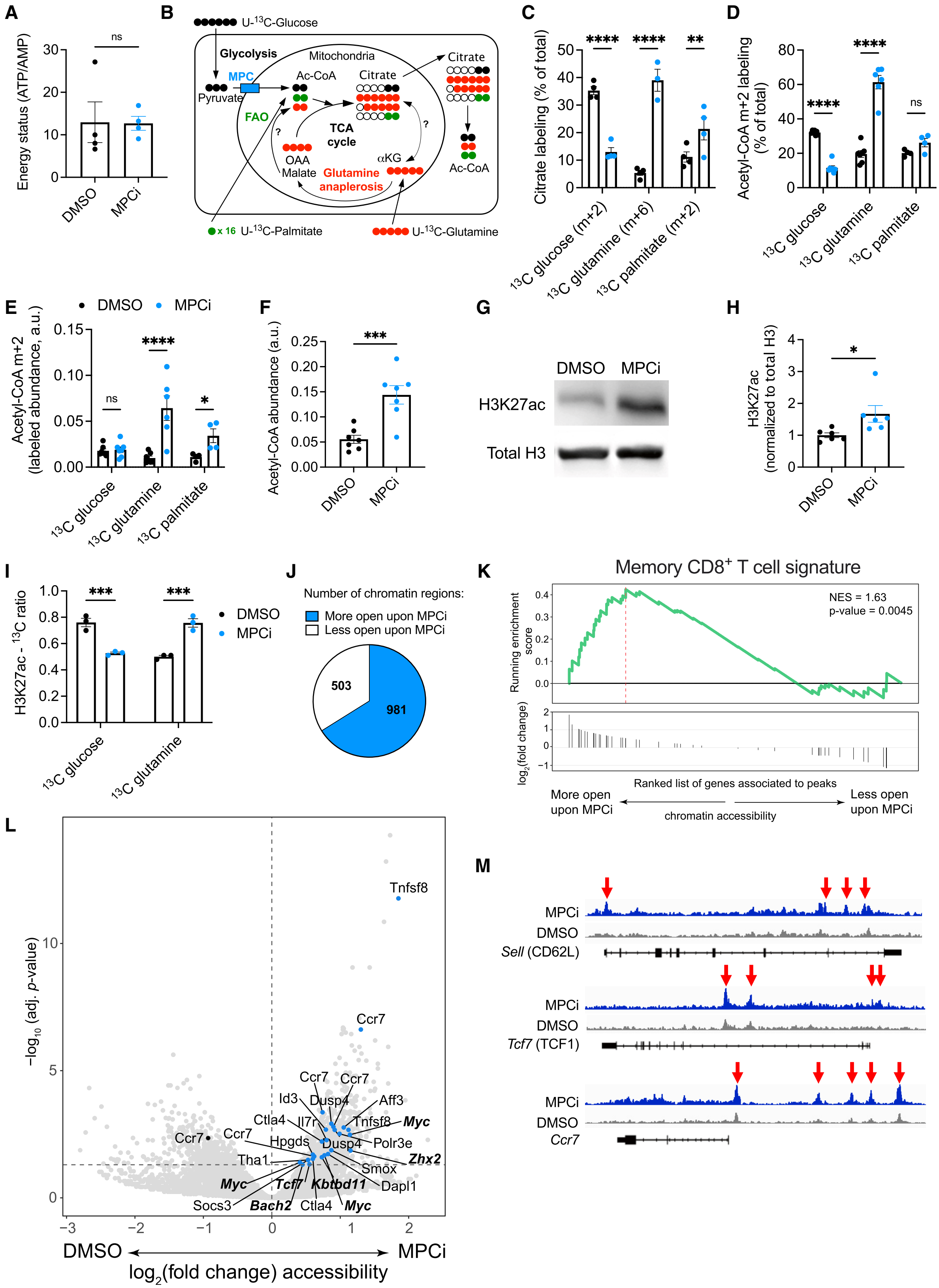

(legend on next page)

formation (Figures 2B and S1D). Acetyl-CoA derived from palmitate was also significantly increased, indicating increased FAO (Figure 2E). The increase in FAO and glutamine oxidation resulted in a significant increase in the overall levels of acetyl-CoA (Figure 2F). High cellular acetyl-CoA levels can facilitate the acetylation of proteins (Choudhary et al., 2014). We analyzed global protein acetylation and observed a slight increase upon MPCi treatment, which was more pronounced around the proteins that might correspond to histones (Figure S2A). Histone acetylation, and in particular the acetylation of lysine residue 27 on histone H3 (H3K27ac), marks active and poised chromatin regions and has been associated with memory $CD8^+$ T cell differentiation (Gray et al., 2017; He et al., 2016). H3K27ac was specifically and strongly increased upon MPC inhibition (Figures 2G, 2H, S2B, and S2C). Consistently, we found that glucose incorporation into the acetyl-group on H3K27 was decreased, whereas glutamine incorporation was increased (Figures 2I, S2D, and S2E). We then evaluated the effect of H3K27ac on chromatin accessibility by ATAC sequencing and identified much more accessible regions in the chromatin of $CD8^+$ T cells upon MPC inhibition (Figure 2J). After annotating accessible chromatin regions to neighboring genes, enrichment analysis showed that a memory $CD8^+$ T cell signature (Dominguez et al., 2015) was significantly enriched in the more accessible regions upon MPC inhibition. Furthermore, several of those pro-memory genes have been found in chromatin regions associated previously with H3K27ac in memory T cells, such as *Sell*, *Tcf7*, and *Ccr7* (Gray et al., 2017) (Figures 2K–2M). Interestingly, genes associated with glutamine metabolism, FAO, and oxidative phosphorylation were also specifically associated with more accessible chromatin regions in $CD8^+$ T cells that were activated with MPCi (Figure S2F).

Altogether, MPC inhibition in $CD8^+$ T cells increases acetyl-CoA formation from glutamine and FAO, which correlates with histone modifications favoring memory gene accessibility.

RUNX1 orchestrates $CD8^+$ T cell memory differentiation upon MPC inhibition

Permissive chromatin at both memory and effector genes is essential for the pluripotency and longevity of memory T cells (Gray et al., 2017). It facilitates the binding of diverse transcription factors, which themselves in turn can promote chromatin accessibility. By using the HOMER motif discovery algorithm, we identified the top 5 transcription factor motifs that were most enriched in the more accessible chromatin regions of $CD8^+$ T cells activated with MPCi. Although the role of CREB3L4 is unknown in immune cells, all 4 other transcription factors (families) have been implicated in effector versus memory $CD8^+$ T cell function (Figure 3A) (Chen et al., 2021b, 2021c; Knudson et al., 2017). In particular, RUNX1 is known to regulate gene transcription in coordination with several other transcription factors, such as NFκB, ETS family of transcription factors, and TCF1 (Chen et al., 2021c; Emmanuel et al., 2018; Luo et al., 2016). Ontology analysis of the genes associated with the more accessible chromatin regions upon MPCi treatment containing a RUNX1 motif revealed IL-2 and CD40 signaling among the most significant pathways (Figure 3B), both of which have been shown to be essential for memory T cell differentiation (Bourgeois et al., 2002). Interestingly, motifs for TCF1, ETS, and RELA were co-enriched at more accessible regions upon MPCi treatment that also contained a RUNX1 motif (Figure 3C). To evaluate whether RUNX1 plays an essential role in the memory $CD8^+$ T cell differentiation upon MPC inhibition, we used CRISPR-Cas9 technology followed by MPCi treatment (Figures 3D and S3A). As observed before, MPCi treatment did not affect the kinetics of the primary $CD8^+$ T cell response when transferred into naive hosts followed by *Listeria*-SIINFEKL infection and neither did RUNX1 deletion (Figure 3E). MPCi skewed $CD8^+$ T cell differentiation from a SLEC to an MPEC phenotype with increased TCF1 expression 14 days post-infection and increased T_{CM} differentiation 4 weeks post-infection. However, these effects were abrogated when RUNX1 was deleted (Figures 3F–3I). RUNX1 deletion per se did not affect the quality of the $CD8^+$ T cell response, as was suggested before (Wang et al., 2018), indicating a *de novo* role in promoting memory $CD8^+$ T cell differentiation following the metabolic rewiring induced by MPC inhibition.

MPC deletion in $CD8^+$ T cells blunts their antitumor potential

A memory T cell phenotype can improve ACT immunotherapy against cancer (Klebanoff et al., 2011). Transfer of MPC1 KO

Figure 2. MPC inhibition in $CD8^+$ T cells induces alternative metabolic fluxes that results in an epigenetic crosstalk and memory imprinting
(A) Energy status (ATP/AMP), 72 h post-activation, based on mass spectrometry data (n = 4 biological replicates).
(B) Schematic representation of metabolite labeling patterns (OAA, oxaloacetate; αKG, α-ketoglutarate).
(C and D) Percentage of the indicated citrate isotope out of total citrate (C) or of m+2 acetyl-CoA out of total acetyl-CoA (D) using different heavy labeled substrates.
(E) Abundance of m+2 acetyl-CoA, derived from different heavy labeled substrates.
(F) Abundance of total cellular acetyl-CoA.
n = 4 biological replicates in (C) and n = 7 biological replicates in (D)–(F); pooled from 2 independent experiments.
(G and H) Representative western blot (G) and quantification shown as fold change compared with DMSO (H) (n = 6 biological replicates; pooled from 4 independent experiments).
(I) Incorporation of carbons derived from glucose or glutamine into the acetyl group on H3K27. Shown here is the ratio of the intensity of the 3rd isotopic peak (containing $2^{*13}C$) over the monoisotopic peak (only ^{12}C) of acetylated peptide KSAPATGGVKKPHR (see also Figures S2H and S2I) (n = 3 biological replicates).
(J) Number of chromatin regions associated with more open or closed regions upon MPCi treatment (n = 3 [DMSO] or 2 [UK5099] biological replicates).
(K) Gene set enrichment analysis of an MPEC signature (Dominguez et al., 2015).
(L) Volcano plot showing the genes associated to more closed (left) or more open (right) chromatin regions upon MPCi treatment. Genes associated with the MPEC signature in (K) are highlighted in blue, with genes previously associated to H3K27ac in bold (Gray et al., 2017).
(M) Representative ATAC-seq traces in and around the gene loci of *Sell*, *Tcf7*, and *Ccr7*. Red arrows highlight increased chromatin accessibility.
Data are represented as mean ± SEM. Statistics are based on unpaired, two-tailed Student's t test (A, F, and H) or two-way ANOVA (C–E and I), $^*p < 0.05$, $^{**}p < 0.01$, $^{***}p < 0.001$, $^{****}p < 0.0001$, and ns ($p > 0.05$). See also Figures S1 and S2.

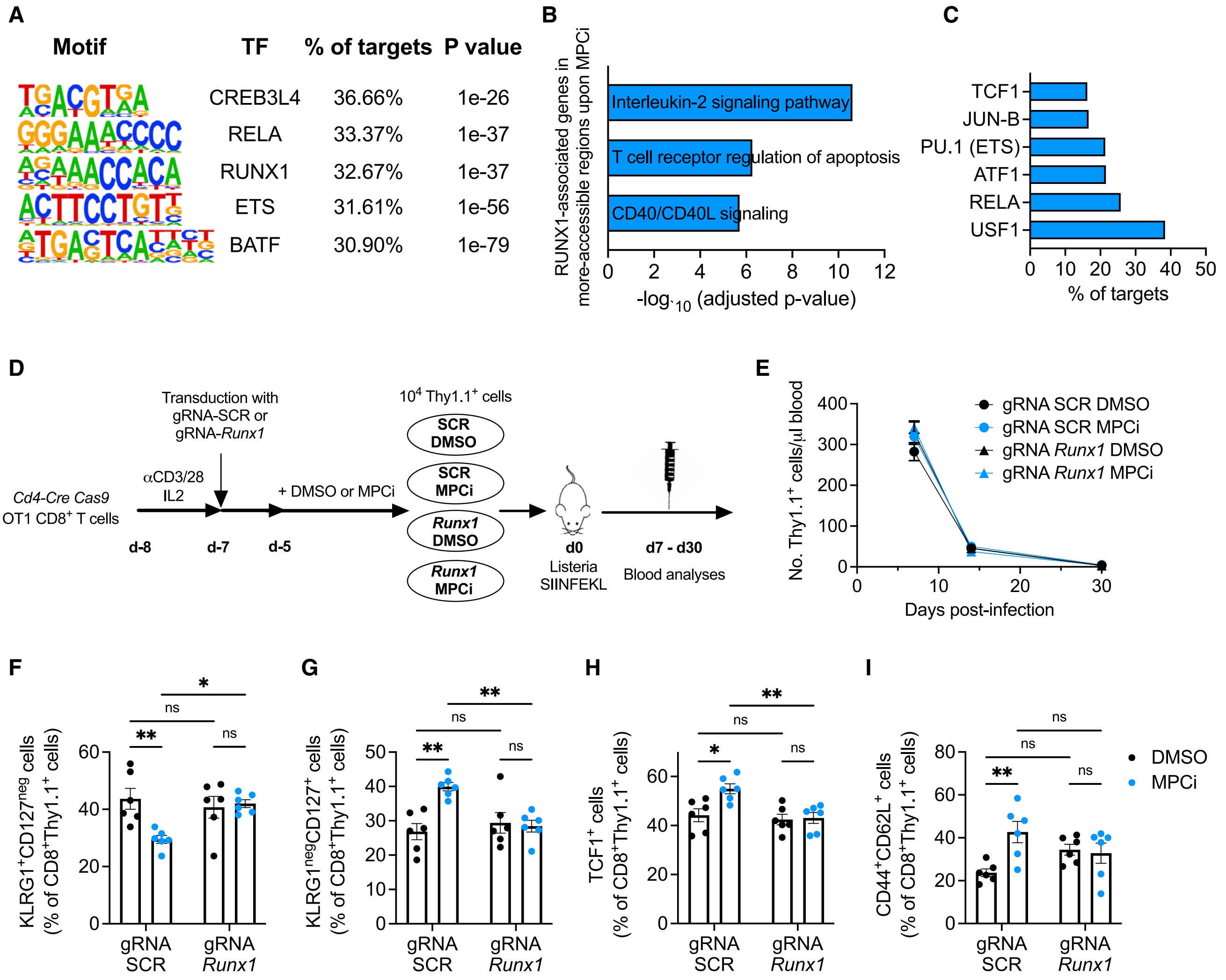

Figure 3. RUNX1 orchestrates CD8+ T cell memory differentiation upon MPC inhibition

(A) Transcription factor motifs in the more accessible regions of T cells treated with MPCi based on the ATAC sequencing data described in Figure 2.

(B) Top three significant Bioplanet 2019 pathways identified by Enrichr software on more accessible genes upon MPCi treatment that contain a RUNX1 transcription factor motif.

(C) Transcription factor motifs in the RUNX1-associated more accessible regions (targets) upon MPCi treatment.

(D) Experimental scheme.

(E) Number of transferred Thy1.1-positive CD8+ T cells in the blood.

(F–I) Percentage of SLECs (F), MPECs (G), and TCF1-positive cells (H) at 2 weeks and T_{CM} cells (I) at 4 weeks post-infection in the blood (n = 6–7 mice/group; pooled data from 2 different experiments).

Data are represented as mean ± SEM. Statistics are based on two-way ANOVA, *p < 0.05, **p < 0.01, and ns (p > 0.05). See also Figure S3.

OT-I CD8+ T cells in tumor-bearing mice (Figure 4A) did not affect T cell quantity but skewed T cell differentiation from an SLEC to MPEC phenotype in the circulating lymphocyte pool and increased T_{CM} differentiation in the spleen (Figures 4B–4E), which is similar to the *Listeria* infection model, despite different antigen levels, innate immune cells, and cytokines involved in both models. Surprisingly, however, MPC1 KO ACT was not able to control tumor growth as compared with untreated mice, whereas WT ACT did (Figures 4F and 4G). Transferred T cell infiltration and viability in the tumor were unaltered (Figures 4H and 4I). Chronic antigen stimulation caused by persistent tumor inflammation induces diverse states of functional exhaustion in CD8+ T cells (Blank et al., 2019). We wondered if the dependence on mitochondrial pyruvate import varies when a CD8+ T cell becomes gradually more terminally exhausted. Using a single-cell tumor-infiltrating lymphocyte (TIL) reference atlas (Andreatta et al., 2021), we observed that *Mpc1* was significantly more expressed in progenitor exhausted (Tpex) than in terminally exhausted (Tex) TILs, whereas *Mpc2* levels remained unaltered (Figure 4J). Accordingly, MPC1 KO CD8+ T cells in the tumor were phenotypically not more terminally exhausted (Figures 4K and 4L), but their progenitor exhausted population was significantly reduced (Figure 4M). As expected, WT tumor-infiltrating CD8+ T cells showed a

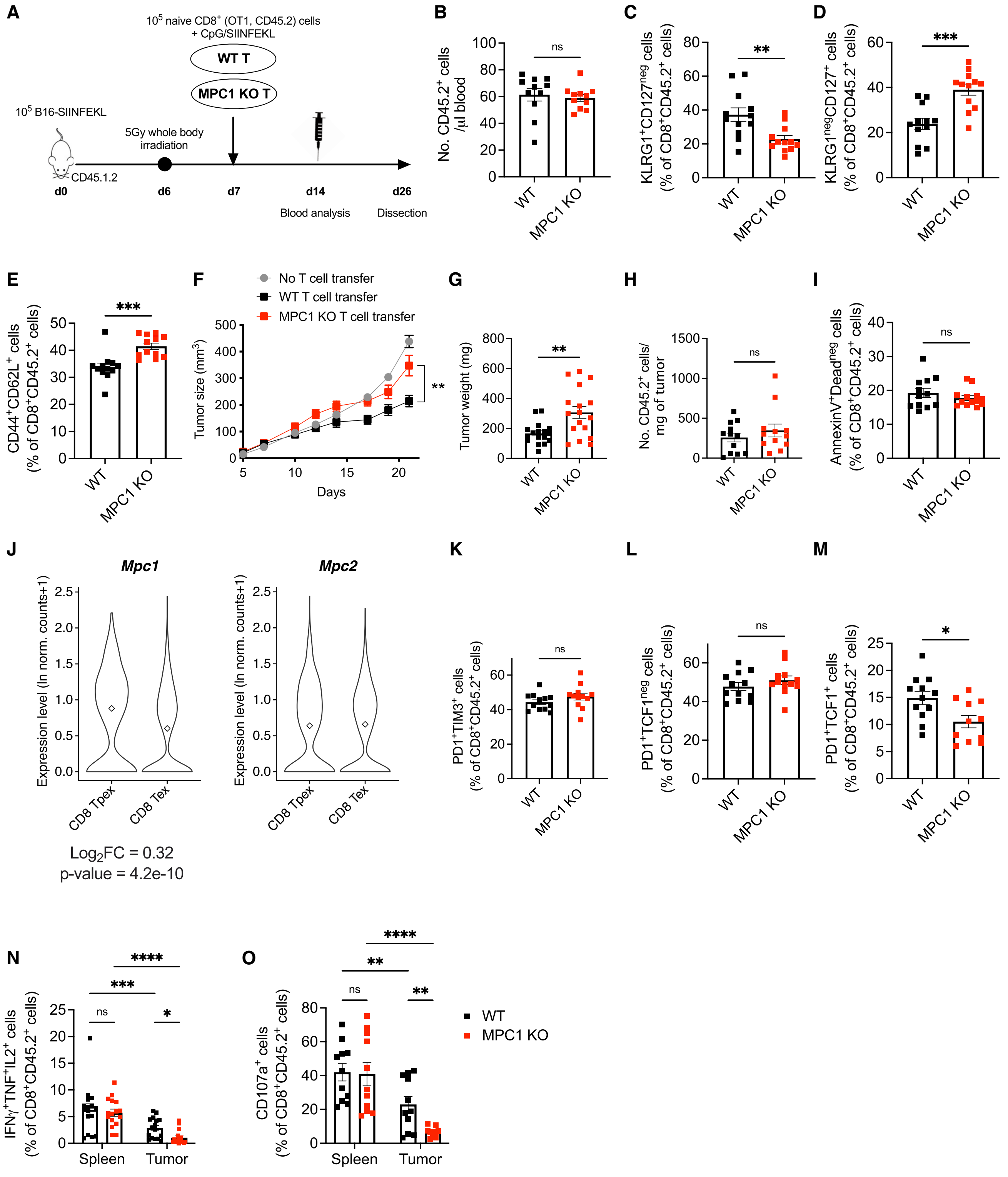

Figure 4. MPC deletion in $CD8^+$ T cells blunts their antitumor potential

(A) Experimental scheme.

(B–D) Number of transferred cells in the blood at 10 days post-transfer (B) and their percentage of SLECs (C) and MPECs (D) (n = 11–12 mice/group; pooled data from 2 independent experiments).

(E) Percentage of T_{CM} cells among transferred cells in the spleen (n = 12 mice/group; pooled data from 2 independent experiments).

(F and G) Tumor growth (F) and weight (G) (n = 13–17 mice/group; pooled data from 3 independent experiments).

(legend continued on next page)

significant suppression of cytokine production and cytotoxic degranulation as compared with those residing in the spleen, however, MPC1 KO T cells displayed a total collapse of their effector function when entering the tumor. Surprisingly, MPC1 deletion did not affect their effector function in the spleen (Figures 4N, 4O, and S4A–S4J).

Altogether, these data surprisingly identify an essential role for mitochondrial pyruvate import in sustaining an antitumor effector function of TILs.

Tumor-infiltrating CD8$^+$ T cells oxidize lactate in their mitochondria to maintain effector function

The severe impairment in effector function of MPC1 KO T cells specifically in the tumor, but not in the spleen, might be explained by the difference in metabolite abundance between those tissues. Low glucose and glutamine levels due to nutrient competition in the tumor microenvironment dampen antitumor T cell function (Edwards et al., 2021; Ho et al., 2015). However, abundant lactate in tumors can be metabolized by lactate dehydrogenase (LDH) to pyruvate and subsequently be oxidized in the mitochondria as shown in both cancer cells and regulatory T cells (Hui et al., 2017; Watson et al., 2021). We recreated this particular metabolic microenvironment by culturing activated WT or MPC1 KO T cells from OT-I mice in either nutrient-rich medium (11 mM glucose and 4 mM glutamine) or nutrient-deprived medium (0.5 mM glucose and 0.1 mM glutamine). To avoid the well-known suppressive effects of low pH, we included to the latter condition sodium L-lactate, whereas control conditions were supplemented with an equimolar amount of sodium chloride (Figure S5A). As in the *in vivo* tumor microenvironment, MPC1 KO CD8$^+$ T cells in nutrient-deprived conditions were as viable as WT T cells (Figure S5B). Upon reactivation, MPC1 KO T cells cultured in nutrient-rich conditions had a similar capacity as WT T cells to produce the cytokines IFNγ, TNF, and IL-2 and were equally pluripotent in producing those cytokines. A nutrient-deprived environment, however, drastically inhibited cytokine production in WT T cells, which was significantly more pronounced in MPC1 KO T cells (Figures 5A, 5B, and S5C–S5F). Interestingly, lactate was able to dose-dependently, but only partially, rescue cytokine expression in WT T cells. MPC1 KO T cells were much less able to rescue cytokine production (Figures 5A, 5B, and S5C–S5F), indicating an inability to metabolize lactate for maintaining effector function. An LDH inhibitor (GSK 2837808A, potently inhibiting both LDHA and LDHB) abrogated the increase in cytokine production in the presence of lactate (Figures 5C and S5G–S5J), indicating an active lactate-to-pyruvate enzymatic conversion activity in this tumor-like metabolic environment. Lactate-derived pyruvate was subsequently imported into the mitochondria through the MPC as more than 50% of each mitochondrial TCA cycle metabolite in WT CD8$^+$ T cells contained carbons derived from ^{13}C-lactate, whereas dramatically less lactate incorporated in MPC1 KO T cells (Figures 5D–5G). Moreover, mitochondrial metabolite abundance was drastically decreased upon MPC1 deletion, suggesting that the general energetic state of those cells was compromised. The major cellular sensor integrating metabolic stress with cellular function is the mammalian target of rapamycin (mTOR). Nutrient-deprived conditions suppressed mTOR activity equally in WT and MPC1 KO T cells. However, only WT cells were able to partially rescue mTOR activity when lactate was included in the culture (Figures 5H and S5K), reflecting the loss of mTOR activity specifically in tumor-infiltrating MPC1 KO CD8$^+$ T cells *in vivo* (Figure 5I). The induction of mTOR activity was essential for the lactate-fueled rescue of cytokine production because this was abrogated dose-dependently by the mTOR inhibitor Torin2 (Figures 5J and S5L–S5O). Finally, the metabolic status of a cell was also in crosstalk with the epigenome. Indeed, WT T cells showed a much stronger increase in H3K27ac than MPC1 KO T cells upon culture in nutrient-deprived conditions with lactate. Furthermore, MPC1 KO T cells had a pronounced increase in the chromatin-condensing trimethylation of H3K27 (Figure 5K). The resulting ratio of open/closed H3K27 mark was, therefore, only significantly rescued by the presence of lactate in WT T cells (Figure 5L).

Altogether, our *in vitro* data suggest that TILs can oxidize lactate in their mitochondria and that this prevents total functional and metabolic collapse in a nutrient-deprived microenvironment by avoiding loss of mTOR signaling and a hypercondensed chromatin conformation.

MPC inhibition results in superior CAR T cell antitumor activity upon ACT immunotherapy

Considering the detrimental effects of MPC1 deletion on T cell function in a tumor microenvironment, we wondered if we could harness the use of an MPCi for imprinting a memory fate in *in vitro,* expanding T cells for ACT immunotherapy without permanently abrogating MPC activity. We, thus, transferred OT-I T cells that were activated and treated *in vitro* with MPCi (“MPCi conditioning”) in B16-SIINFEKL tumor-bearing mice. MPCi conditioning significantly enhanced the antitumor function of OT-I T cells as compared with DMSO (Figures 6A and S6A). At dissection, MPCi-conditioned T cells were more abundant in the spleen and draining lymph node and formed more T_{CM} cells (Figures 6B, 6C, and S6B). In the tumor, MPCi-conditioned

(H) T cell infiltration in tumors (n = 12 mice/group; pooled data from 2 independent experiments).
(I) Apoptotic tumor-infiltrating T cells (n = 12–13 mice/group; pooled data from 2 independent experiments).
(J) Expression level of *Mpc1* and *Mpc2* in cell clusters identified as progenitor exhausted (Tpex) and terminally exhausted (Tex) CD8$^+$ T cells based on single-cell RNA-seq data (source: https://spica.unil.ch/), reported as normalized gene expression (ln norm. counts +1).
(K) Tumor-infiltrating T cells co-expressing PD1 and TIM3.
(L and M) Percentage of Tex (L) and Tpex (M) cells among the tumor-infiltrating transferred T cells (n = 11–12 mice/group; pooled data from 2 independent experiments).
(N and O) Percentage of splenic or tumor T cells expressing IFNγ, TNF, and IL-2 (N) or CD107a (O) (n = 16 mice/group; pooled data from 3 independent experiments, as shown in N, and n = 11 mice/group; pooled data from 2 independent experiments, as shown in O).
Data are represented as mean ± SEM. Statistics are based on unpaired, two-tailed Student’s t test (B–I and K–M), Wilcoxon test (J), or two-way ANOVA (N and O), *p < 0.05, **p < 0.01, ***p < 0.001, ****p < 0.0001, and ns (p > 0.05). See also Figure S4.

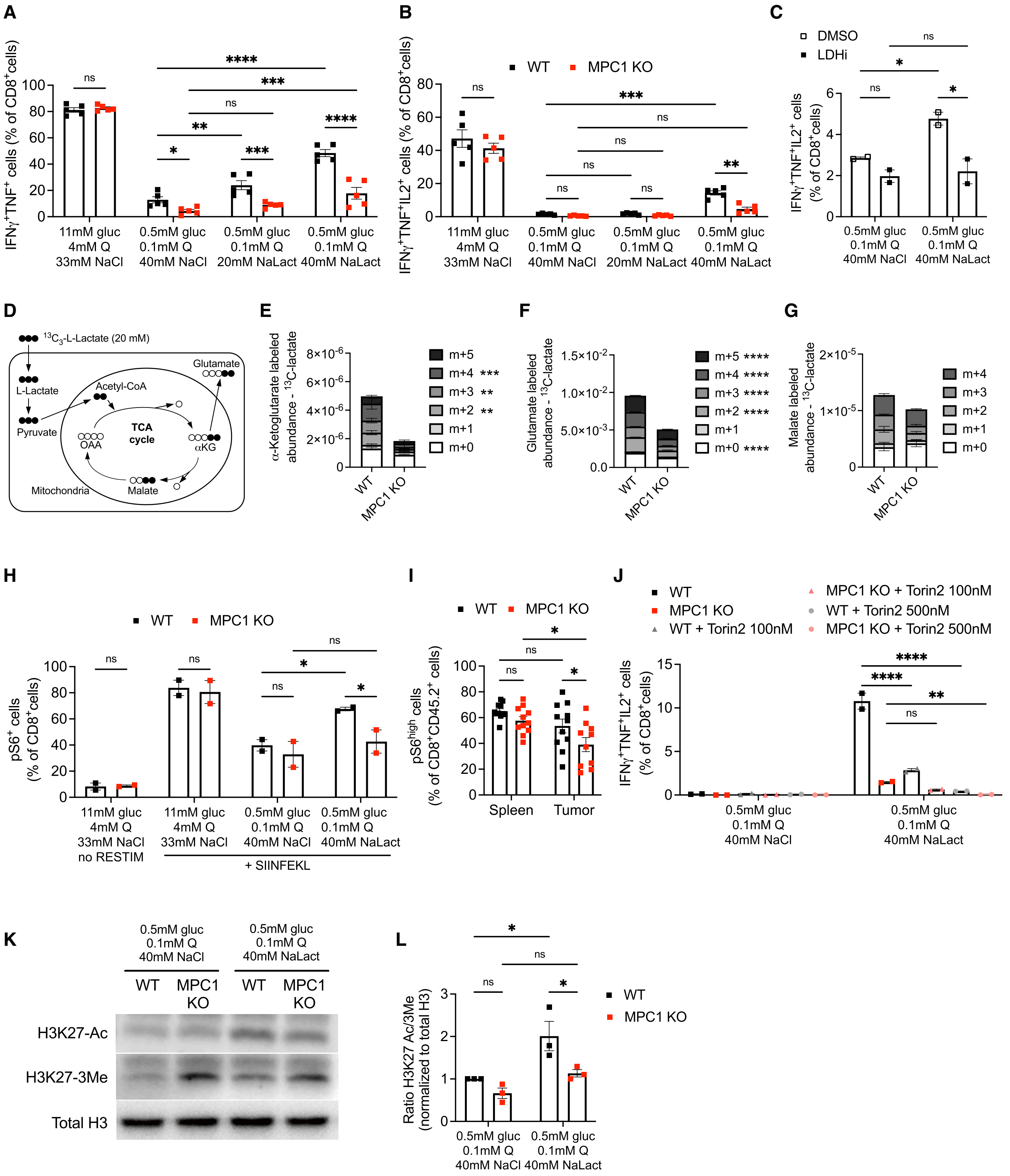

Figure 5. Tumor-infiltrating CD8+ T cells oxidize lactate in their mitochondria to maintain effector function

(A and B) Cytokine expression (n = 5 biological replicates/genotype; pooled data from 2 independent experiments).

(C) Cytokine expression in the presence of 25 μM LDHA/B inhibitor GSK 2837808A (LDHi) or DMSO control (n = 2 biological replicates/group; pooled data from 2 independent experiments).

(D) Schematic representation of the metabolic pathways allowing for the detection of carbon incorporation in TCA metabolites derived from uniformly labeled ^{13}C-L-lactate.

(legend continued on next page)

T cell number was increased (Figure 6D), and they expressed more TCF1; although there was no difference in number of progenitor exhausted T cells, terminally exhausted T cells were decreased (Figures 6E–6H and S6C–S6E). Splenic MPCi-conditioned T cells showed an increased pluripotency with more T cells being double and triple positive for IFNγ, TNF, and IL-2 (Figures S6F–S6I). Tumor-infiltrating MPCi-conditioned T cells showed an increase in cells double positive for the effector cytokines IFNγ and TNF (Figures 6I and S6L). Although the percentage of granzyme B-positive cells was not altered, the absolute numbers in both spleen and tumor were increased upon MPCi conditioning (Figures S6J, S6K, S6M, and S6N). Next, we transduced polyclonal $CD8^+$ T cells with a CAR construct targeting the human oncogene HER2 or with a blue fluorescent protein (BFP) as control and exposed the cells to DMSO or MPCi (Figure S6O). MPCi treatment did not affect transduction efficiency but strongly induced acetylation on histone H3K27 (Figures S6P and S6Q). Upon ACT in mice bearing B16 tumors overexpressing HER2, we observed that only the MPCi-conditioned HER2-CAR T cells were able to significantly control tumor growth (Figures 6J and 6K). Twelve days post-ACT, CAR T cell number was not altered, but MPCi-conditioned HER2-CAR T cells were skewed from an SLEC to MPEC phenotype and expressed more TCF1 (Figures 6L, 6M, S6R, and S6S). At dissection, we observed a higher abundance of MPCi-conditioned CAR T cells in the tumor-draining lymph node, whereas both DMSO- and MPCi-conditioned T cells showed high T_{CM} differentiation (Figures 6N and 6O). In the spleen, CAR T cell abundance was unaltered, but MPCi-conditioned CAR T cells expressed significantly more TCF1 (Figures 6P and 6Q). MPCi-conditioned CAR T cells infiltrated the tumor more and expressed more TCF1 (Figures 6R and 6S). The progenitor exhausted CAR T cell population was increased, whereas terminal differentiation was decreased upon MPCi conditioning (Figures 6T–6V).

MPC inhibition dramatically improves human CD19-CAR T cell therapy in a xenograft leukemia model

To translate our findings to human T cells, peripheral blood mononuclear cells from 7 healthy donors were activated in the presence of DMSO or MPCi (Figure 7A). MPCi treatment did not affect the final yield of $CD8^+$ T cells (Figure 7B) but significantly increased CD62L expression (Figures 7C–7E). MPC inhibition also induced the expression of stem cell-like phenotypic markers and increased H3K27 acetylation (Figures 7F and 7G). Interestingly, a similar phenotypic memory shift was observed in $CD4^+$ T cells (Figures S7A–S7C). We then tested MPCi conditioning in a more clinical-like manufacturing of an anti-CD19 CAR (Figure 7H). Similar to murine CAR T cells, MPCi did not affect transduction efficiency and enhanced the expression of CD62L and a stem cell-like memory phenotype (Figures 7I, 7J, and S7D). We then transferred a sub-therapeutic dose of CAR T cells into NOD *SCID*-γ mice that had advanced and widespread NALM6 acute lymphoblastic leukemia. Whereas DMSO-conditioned CD19-CAR T cells only slightly improved mouse survival as compared with untreated mice or mice treated with non-transduced T cells, MPCi-conditioned CD19-CAR T cell treatment resulted in the remarkable survival of all mice (Figures 7K and S7E). MPCi-conditioned CD19-CAR T cells strongly reduced NALM6 numbers in the blood by day 7 post-ACT, up to undetectable levels 12 days post-ACT (Figure 7L). $CD4^+$ and $CD8^+$ MPCi-conditioned CD19-CAR T cells contracted by 4 weeks post-ACT but were still abundantly detectable in the blood and phenotypically contributed to the effector, central, and even stem cell memory pool (Figures S7F–S7H).

In conclusion, the inhibition of the MPC allows a metabolic compensation in a nutrient-rich environment that induces a stable memory-like epigenetic conformation, which can be applied during T cell product manufacturing for ACT to enhance their antitumor potency and persistence upon adoptive transfer.

DISCUSSION

Here, we demonstrated that mitochondrial pyruvate import determines $CD8^+$ T cell fate and function depending on the microenvironmental nutrient availability. Stromal cells infiltrating a solid tumor undergo metabolic competition with rapidly proliferating cancer cells. Both nutrient deprivation (Bian et al., 2020; Edwards et al., 2021; Ho et al., 2015) and an acidic pH due to lactic acid production (Brand et al., 2016; Husain et al., 2013) dampen the antitumor effector response of $CD8^+$ T cells and NK cells and promote myeloid-derived suppressor cells. Lactate, however, has also been proposed as a carbon source, which is oxidized in the mitochondria, both in healthy tissues (Hui et al., 2017) and cancer cells (Faubert et al., 2017). Moreover, cytotoxic $CD8^+$ T cells can be fueled by exercise-induced circulating lactate (Rundqvist et al., 2020). We show here that tumor-infiltrating cytotoxic $CD8^+$ T cells, at least in our *in vitro* model, depend on lactate metabolism to sustain their antitumor function. Interestingly, *Mpc1* levels were elevated in Tpex, a TIL subset essential for maintaining antitumor function and for response to immune checkpoint blockade (Miller et al., 2019; Siddiqui et al., 2019), and MPC1 deletion significantly reduced their numbers in the tumor. The oxidative metabolism of this TIL subset might be related to its preferential localization near blood vessels and, thus, its experience of higher oxygen tensions (Siddiqui et al., 2019). A similar metabolic symbiosis has been described in tumors in which cancer cells located near blood vessels were oxidizing lactate produced by glycolytic

(E–G) Relative abundance of α-ketoglutarate, glutamate, and malate in T cells cultured for 18 h in nutrient-deprived medium containing 20 mM of uniformly labeled ^{13}C-L-lactate (“metabolite + n” equals the molecular mass plus the number of incorporated heavy carbons) (n = 3 biological replicates).
(H) Percentage of T cells positive for phosphorylated S6 (serine 235–236) protein (n = 2 biological replicates from 2 independent experiments).
(I) Percentage of phosphorylated S6-positive spleen or B16-SIINFEKL tumor-infiltrating T cells (n = 11 mice/group; pooled data from 2 independent experiments).
(J) Cytokine production in the presence of the mTOR inhibitor Torin2 (n = 2 biological replicates/genotype; pooled data from 2 independent experiments).
(K and L) Representative western blot (K) and quantification of the ratio of acetylation over methylation shown as fold change compared with WT T cells starved in 0.5 mM glucose, 0.1 mM glutamine, and 40 mM NaCl (L) (n = 3 biological replicates/genotype; pooled from 3 independent experiments).
Data are represented as mean ± SEM. Statistics are based on two-way ANOVA, *p < 0.05, **p < 0.01, ***p < 0.001, ****p < 0.0001, and ns (p > 0.05). See also Figure S5.

CellPress
OPEN ACCESS

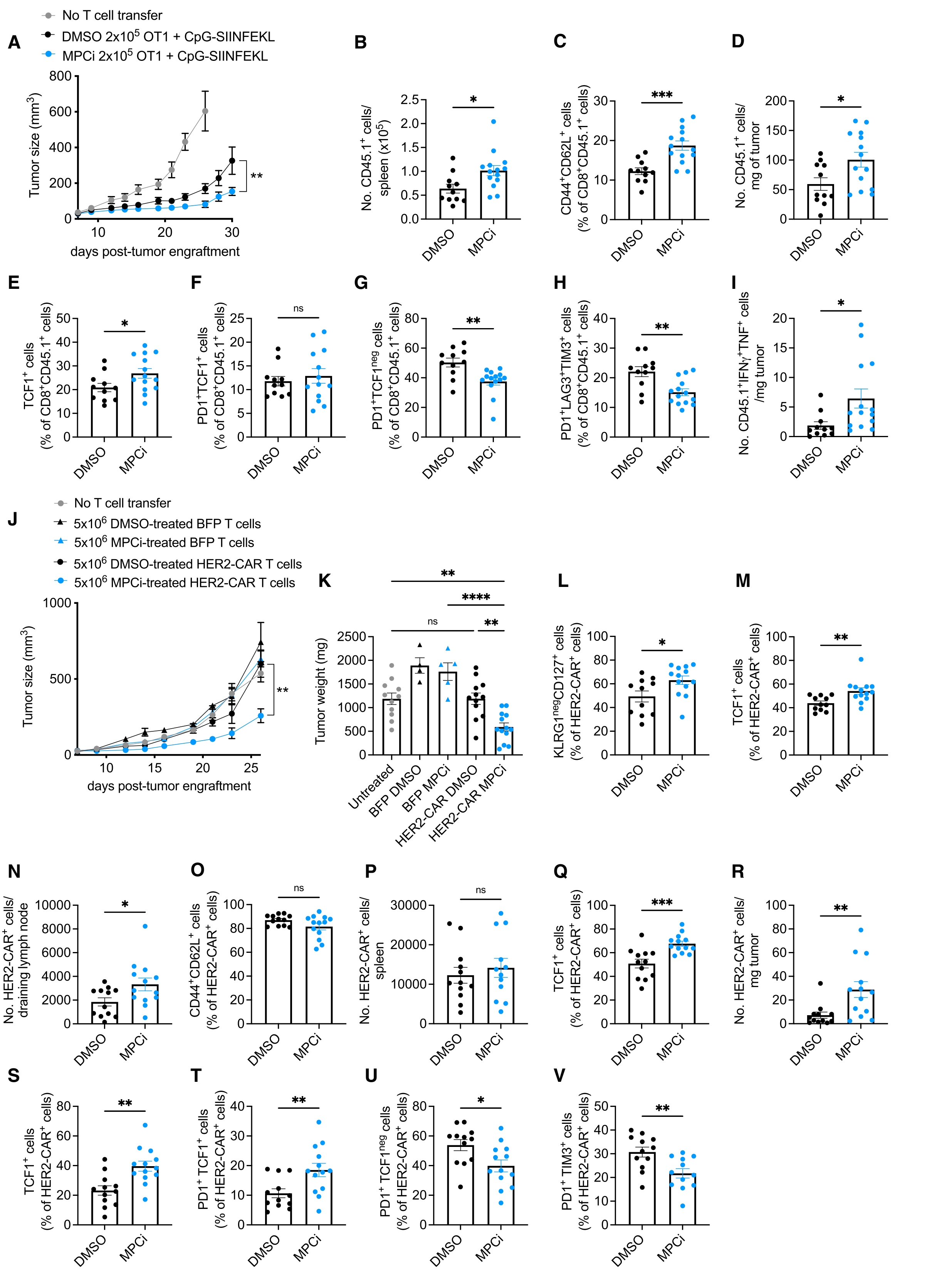

(legend on next page)

hypoxic cancer cells (Sonveaux et al., 2008). In contrast, terminally exhausted T cells are characterized by a hypoxic gene signature, which is associated with mitochondrial defects (Yu et al., 2020). Interestingly, the proliferation and antitumor function of this checkpoint blockade-insensitive TIL subset can be restored by IL-10 administration, which critically depends on MPC activity (Guo et al., 2021). In light of our data, IL-10 might actually favor lactate oxidation in terminally exhausted T cells, and, thereby, reinvigorate this TIL subset by replenishing energy stores and preventing epigenetic exhaustion.

Outside the tumor microenvironment, where nutrients are more abundant, CD8$^+$ T cells show a remarkable metabolic plasticity upon inhibition of mitochondrial pyruvate import, resulting in high acetyl-CoA levels that facilitate histone acetylation and chromatin accessibility. The RUNX family of transcription factors have been shown to control chromatin state through their interaction with the histone acetyltransferase p300 during hematopoiesis (Aikawa et al., 2006) or as a key initiating transcription factor driving chromatin accessibility in cytotoxic T cells early after activation (van der Veeken et al., 2019; Wang et al., 2018). Interestingly, RUNX1 overexpression favors the differentiation of a memory-like progenitor exhausted CD8$^+$ T cell population in chronic infection in mice (Chen et al., 2021c). In our study, however, RUNX1 deletion did not affect T cell differentiation upon DMSO-conditioning in acute infection. How RUNX1 acquires the apparent gain-of-function activity as pro-memory differentiation transcription factor upon MPC inhibition and how this can be related to increases in cellular acetyl-CoA levels will be a subject for future investigation.

Because the epigenetic imprinting of a memory phenotype could be achieved through small molecule inhibition of MPC *in vitro*, we saw an opportunity to harness the memory-inducing effect of MPC inhibition for CAR T cell manufacturing while avoiding T cell functional collapse in the nutrient-deprived tumor microenvironment upon systemic MPC inhibition. Indeed, epigenetic intervention with small molecules or metabolites during CAR T cell manufacturing is an emerging strategy to either prevent loss of CAR expression and exhaustion (Kong et al., 2021) or induce a memory phenotype (Foskolou et al., 2020). While it also efficiently induces memory differentiation, interfering with glycolysis to optimize ACT has proven to negatively affect CAR T cell yield (Klein Geltink et al., 2020; Sukumar et al., 2013). We propose to target the glycolytic pathway more downstream by inhibiting mitochondrial pyruvate entry, which did not affect T cell yield or activation, when culture conditions allowed metabolic compensation. Conditioning CAR T cells with an MPCi induced chromatin accessibility at pro-memory genes and dramatically improved their antitumor effects in both an immunocompetent mouse solid tumor model and a xenograft human leukemia model.

Limitations of study

Here, we inhibit the MPC with the small molecule UK5099, which was shown to inhibit pyruvate-dependent oxygen consumption decades before the identification of the MPC (Halestrap, 1975). Although we could confirm the specificity of memory CD8$^+$ T cell induction using a genetic model of MPC deletion, this molecule contains a reactive group that is potentially a covalent binder. Therefore, for successful clinical translation of our findings, there is an urgent need for the development of a next generation MPCi.

Finally, we have exclusively used an *in vitro* model to mimic the microenvironmental nutrient composition. It is technically challenging to demonstrate lactate oxidation *in vivo* in TILs, and without such evidence, it is possible that in an *in vivo* situation, CD8$^+$ T cells infiltrating the tumor are not dependent on lactate consumption for sustaining their cytotoxic effector functions.

STAR★METHODS

Detailed methods are provided in the online version of this paper and include the following:

- KEY RESOURCES TABLE
- RESOURCE AVAILABILITY
  - Lead contact
  - Materials availability
  - Data and code availability
- EXPERIMENTAL MODEL AND SUBJECT DETAILS
  - Mice
  - Primary cell culture
  - Cell lines
- METHOD DETAILS
  - Mouse T cell culture

Figure 6. MPC inhibition during CAR T cell *in vitro* activation and expansion induces superior antitumor activity upon ACT in a mouse melanoma model

(A) Tumor growth following ACT of DMSO or UK5099 (MPCi)-conditioned OT1 T cells.

(B and C) Number of transferred cells (B) and their percentage of T_{CM} (C) in the spleen.

(D–H) Number of tumor-infiltrating transferred cells per milligram of tumor (D) and their percentage of TCF1-positive cells (E), Tpex (F), Tex (G), and cells co-expressing PD1, LAG3, and TIM3 (H).

(I) Number of tumor-infiltrating transferred cells co-expressing IFNγ and TNF.

In (A)–(I), n = 11 mice (DMSO) and 14 mice (MPCi); pooled data from 2 independent experiments.

(J and K) Tumor growth (J) and weight (K) of B16-HER2 tumors following treatment with DMSO or MPCi-conditioned HER2-CAR or BFP control T cells.

(L and M) Percentage of T_{CM} cells (L) and TCF1-expressing cells (M) out of HER2-CAR-positive cells in the blood 12 days after ACT.

(N and O) Number of HER2-CAR-positive cells (N) and their percentage of T_{CM} (O) in the tumor-draining lymph node.

(P and Q) Number of HER2-CAR-positive cells (P) and their percentage of TCF1-positive cells (Q) in the spleen.

(R–V) Number of tumor-infiltrating HER2-CAR-positive cells per milligram of tumor (R) and their percentage of TCF1-positive cells (S), Tpex (T), Tex (U), and cells co-expressing PD1 and TIM3 (V).

In (J)–(V), n = 11 mice (untreated), 4–5 mice (DMSO- and MPCi-BFP-T ACT), 12–13 mice (DMSO- and MPCi-HER2-CAR T ACT). BFP data are derived from 1 experiment; untreated and HER2-CAR T cell transfer is pooled data from 2 independent experiments.

Data are represented as mean ± SEM. Statistics are based on unpaired, two-tailed Student's t test (A–I and L–V) or one-way ANOVA (J and K), *$p < 0.05$, **$p < 0.01$, ***$p < 0.001$, ****$p < 0.0001$, and ns ($p > 0.05$). See also Figure S6.

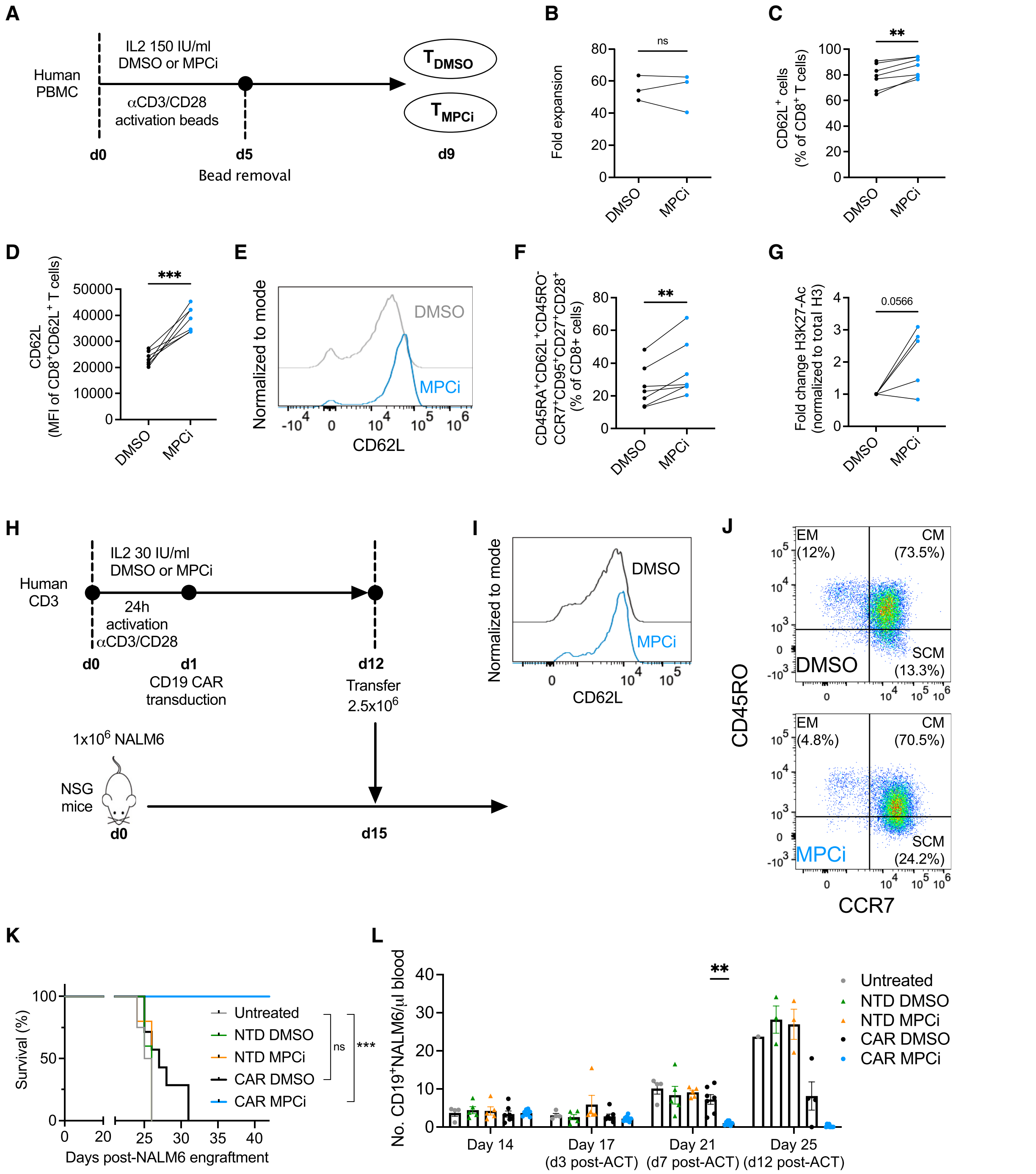

Figure 7. MPC inhibition dramatically improves human CD19-CAR T cell therapy in a xenograft leukemia model

(A) Experimental scheme.

(B) Fold expansion after 9 days of culture (n = 3 human donors/group).

(C–E) Percentage CD62L-positive $CD8^+$ T cells (C) and median fluorescent intensity (MFI) of CD62L in the CD62L-positive population (D), and representative histogram from one donor (E).

(F) Percentage of stem cell-like memory $CD8^+$ T cells, measured by flow cytometry.

In (C)–(F), n = 7 human donors/group; pooled data from 2 independent experiments.

(G) Western blot quantification of H3K27 acetylation shown as fold change compared with DMSO (n = 5 human donors/group; pooled data from 2 independent experiments).

(H) Experimental scheme.

(I and J) Histogram of CD62L expression (I) and FACS plot showing CD45-RO and CCR7 expression (J) in $CD8^+$ T cells from a representative donor, 7 days after transduction (EM, effector memory; CM, central memory; SCM, stem cell-like memory).

(legend continued on next page)

- ○ Preparation of mouse CAR T cells
- ○ Human T cell culture
- ○ Preparation of human CAR T cells
- ○ *Listeria* model of infection
- ○ Tumor models
- ○ ^{13}C-labeling and LC-MS/MS metabolomics
- ○ Histone 13C acetylation
- ○ ATAC-seq analysis
- ○ RUNX1 deletion by CRISPR-Cas9
- ○ Flow cytometry
- ○ Western blot
- ● QUANTIFICATION AND STATISTICAL ANALYSIS

SUPPLEMENTAL INFORMATION

Supplemental information can be found online at https://doi.org/10.1016/j.cmet.2022.03.013.

ACKNOWLEDGMENTS

We thank Jarred Rutter and Eric Taylor for their permission to use the *Mpc*$^{fl/fl}$ mice. We thank Jean-Claude Martinou for scientific discussion. ATAC sequencing was performed at the Gene Expression Core Facility at the École Polytechnique Fédéral de Lausanne, Switzerland. Metabolomics studies were performed at the MSU Mass Spectrometry and Metabolomics Core, USA. M.W., P.-C.H., and P.R. were supported in part by a grant from Roche. P.R. is supported in part by SNSF grant no. 310030_182735 and Oncosuisse KFS-4404-02-2018. P.-C.H. is funded in part by the European Research Council starting grant (802773-MitoGuide), SNSF project grants (31003A_182470), the Cancer Research Institute (Lloyd J. Old STAR award), Ludwig Cancer Research, and Elixiron Immunotherapeutics. S.T.T. and S.Y.L. are supported by a METAvivor Early Career Investigator grant to S.Y.L. N.M.-P. was funded by the European Molecular Biology Organization (EMBO) and the European Federation of Immunological Societies (EFIS). D.M. receives support from the ISREC Foundation, Association Frédéric Fellay, Ernst and Lucie Schmidheiny Foundation, Fondation Dr Henri Dubois-Ferrière Dinu Lipatti (DFDL), Ligue Genevoise contre le cancer (LGC_2016), Personalized Health and Related Technologies (PHRT) ETH fund (2021_355), Swiss Bridge Foundation (N-ME11711), and Swiss Innovation Agency (Innosuisse, 47046.1 IP-LS).

AUTHOR CONTRIBUTIONS

M.W. conceived, designed, and performed most experiments, and wrote the manuscript; A.J. discussed data and performed experiments; T.W. performed bioinformatics analyses; N.M.-P. and F.M. performed experiments on human T cells; and S.T.T. and S.Y.L. helped design and perform metabolomics studies. A.L. and D.M. produced the anti-CD19 human CAR T cells and designed the *in vivo* experiment. F.R. performed *in vivo* experiments, F.F. cloned gRNA retroviral plasmids, and P.W. measured ^{13}C tracing in histones. B.T. and A.D. designed the HER2 CAR T cell model. C.Y.M. and N.D. provided technical assistance. L.Z. provided scientific input; P.-C.H. provided reagents and scientific input; and P.R. gave scientific input, co-wrote the manuscript, and provided overall direction.

DECLARATION OF INTERESTS

M.W. and P.R. are inventors on a patent application related to these findings and have research collaborations with MPC Therapeutics, with P.R. being a member of the advisory board. D.M. is an inventor on patents related to CAR T cell therapy, filed by the University of Pennsylvania and the University of Geneva, and is a consultant for Limula Therapeutics and MPC Therapeutics. P.-C.H. is scientific advisory for Elixiron Immunotherapeutics, Acepodia, and Novartis. P.-C.H. also receives research support from Elixiron Immunotherapeutics. S.Y.L. is a consultant to Senda Biosciences.

Received: September 28, 2021
Revised: January 25, 2022
Accepted: March 30, 2022
Published: April 21, 2022

REFERENCES

Aikawa, Y., Nguyen, L.A., Isono, K., Takakura, N., Tagata, Y., Schmitz, M.L., Koseki, H., and Kitabayashi, I. (2006). Roles of HIPK1 and HIPK2 in AML1- and p300-dependent transcription, hematopoiesis and blood vessel formation. EMBO J. *25*, 3955–3965.

Andreatta, M., Corria-Osorio, J., Müller, S., Cubas, R., Coukos, G., and Carmona, S.J. (2021). Interpretation of T cell states from single-cell transcriptomics data using reference atlases. Nat. Commun. *12*, 2965.

Bian, Y., Li, W., Kremer, D.M., Sajjakulnukit, P., Li, S., Crespo, J., Nwosu, Z.C., Zhang, L., Czerwonka, A., Pawłowska, A., et al. (2020). Cancer SLC43A2 alters T cell methionine metabolism and histone methylation. Nature *585*, 277–282.

Blank, C.U., Haining, W.N., Held, W., Hogan, P.G., Kallies, A., Lugli, E., Lynn, R.C., Philip, M., Rao, A., Restifo, N.P., et al. (2019). Defining 'T cell exhaustion'. Nat. Rev. Immunol. *19*, 665–674.

Bourgeois, C., Rocha, B., and Tanchot, C. (2002). A role for CD40 expression on CD8+ T cells in the generation of CD8+ T cell memory. Science *297*, 2060–2063.

Brand, A., Singer, K., Koehl, G.E., Kolitzus, M., Schoenhammer, G., Thiel, A., Matos, C., Bruss, C., Klobuch, S., Peter, K., et al. (2016). LDHA-associated lactic acid production blunts tumor immunosurveillance by T and NK cells. Cell Metab. *24*, 657–671.

Bricker, D.K., Taylor, E.B., Schell, J.C., Orsak, T., Boutron, A., Chen, Y.C., Cox, J.E., Cardon, C.M., Van Vranken, J.G., Dephoure, N., et al. (2012). A mitochondrial pyruvate carrier required for pyruvate uptake in yeast, Drosophila, and humans. Science *337*, 96–100.

Buenrostro, J.D., Giresi, P.G., Zaba, L.C., Chang, H.Y., and Greenleaf, W.J. (2013). Transposition of native chromatin for fast and sensitive epigenomic profiling of open chromatin, DNA-binding proteins and nucleosome position. Nat. Methods *10*, 1213–1218.

Buenrostro, J.D., Wu, B., Chang, H.Y., and Greenleaf, W.J. (2015). ATAC-seq: A method for assaying chromatin accessibility genome-wide. Curr. Protoc. Mol. Biol. *109*, 21.29.1–21.29.9.

Chen, G.M., Chen, C., Das, R.K., Gao, P., Chen, C.H., Bandyopadhyay, S., Ding, Y.Y., Uzun, Y., Yu, W., Zhu, Q., et al. (2021a). Integrative bulk and single-cell profiling of premanufacture T-cell populations reveals factors mediating long-term persistence of CAR T-cell therapy. Cancer Discov. *11*, 2186–2199.

Chen, Y., Zander, R.A., Wu, X., Schauder, D.M., Kasmani, M.Y., Shen, J., Zheng, S., Burns, R., Taparowsky, E.J., and Cui, W. (2021b). BATF regulates progenitor to cytolytic effector CD8(+) T cell transition during chronic viral infection. Nat. Immunol. *22*, 996–1007.

Chen, Z., Arai, E., Khan, O., Zhang, Z., Ngiow, S.F., He, Y., Huang, H., Manne, S., Cao, Z., Baxter, A.E., et al. (2021c). In vivo CD8(+) T cell CRISPR screening reveals control by Fli1 in infection and cancer. Cell *184*, 1262–1280.e22.

Choudhary, C., Weinert, B.T., Nishida, Y., Verdin, E., and Mann, M. (2014). The growing landscape of lysine acetylation links metabolism and cell signalling. Nat. Rev. Mol. Cell Biol. *15*, 536–550.

(K) Overall survival.
(L) Number of NALM6 cells in the blood (n = 4 mice for untreated, n = 5 mice for NTD DMSO and NTD MPCi, n = 7 mice for CAR DMSO, and n = 8 mice for CAR MPCi; pooled data from 2 independent experiments).
Data are represented as mean ± SEM. Statistics are based on paired, two-tailed Student's t test (B–D and F–G), log rank test (K), or two-way ANOVA using Fisher's LSD test (L), *p < 0.05, **p < 0.01, ***p < 0.001, or as indicated. See also Figure S7.

CellPress
OPEN ACCESS

Coren, L.V., Jain, S., Trivett, M.T., Ohlen, C., and Ott, D.E. (2015). Production of retroviral constructs for effective transfer and expression of T-cell receptor genes using Golden Gate cloning. BioTechniques *58*, 135–139.

Cui, G., Staron, M.M., Gray, S.M., Ho, P.C., Amezquita, R.A., Wu, J., and Kaech, S.M. (2015). IL-7-induced glycerol transport and TAG synthesis promotes memory CD8+ T cell longevity. Cell *161*, 750–761.

de Hoon, M.J., Imoto, S., Nolan, J., and Miyano, S. (2004). Open source clustering software. Bioinformatics *20*, 1453–1454.

Doench, J.G., Fusi, N., Sullender, M., Hegde, M., Vaimberg, E.W., Donovan, K.F., Smith, I., Tothova, Z., Wilen, C., Orchard, R., et al. (2016). Optimized sgRNA design to maximize activity and minimize off-target effects of CRISPR-Cas9. Nat. Biotechnol. *34*, 184–191.

Dominguez, C.X., Amezquita, R.A., Guan, T., Marshall, H.D., Joshi, N.S., Kleinstein, S.H., and Kaech, S.M. (2015). The transcription factors ZEB2 and T-bet cooperate to program cytotoxic T cell terminal differentiation in response to LCMV viral infection. J. Exp. Med. *212*, 2041–2056.

Edwards, D.N., Ngwa, V.M., Raybuck, A.L., Wang, S., Hwang, Y., Kim, L.C., Cho, S.H., Paik, Y., Wang, Q., Zhang, S., et al. (2021). Selective glutamine metabolism inhibition in tumor cells improves antitumor T lymphocyte activity in triple-negative breast cancer. J. Clin. Invest. *131*, e140100.

Emmanuel, A.O., Arnovitz, S., Haghi, L., Mathur, P.S., Mondal, S., Quandt, J., Okoreeh, M.K., Maienschein-Cline, M., Khazaie, K., Dose, M., and Gounari, F. (2018). TCF-1 and HEB cooperate to establish the epigenetic and transcription profiles of $CD4^+$ $CD8^+$ thymocytes. Nat. Immunol. *19*, 1366–1378.

Faubert, B., Li, K.Y., Cai, L., Hensley, C.T., Kim, J., Zacharias, L.G., Yang, C., Do, Q.N., Doucette, S., Burguete, D., et al. (2017). Lactate metabolism in human lung tumors. Cell *171*, 358–371.e9.

Foskolou, I.P., Barbieri, L., Vernet, A., Bargiela, D., Cunha, P.P., Velica, P., Suh, E., Pietsch, S., Matuleviciute, R., Rundqvist, H., et al. (2020). The S enantiomer of 2-hydroxyglutarate increases central memory CD8 populations and improves CAR-T therapy outcome. Blood Adv. *4*, 4483–4493.

Fraietta, J.A., Nobles, C.L., Sammons, M.A., Lundh, S., Carty, S.A., Reich, T.J., Cogdill, A.P., Morrissette, J.J.D., DeNizio, J.E., Reddy, S., et al. (2018). Disruption of TET2 promotes the therapeutic efficacy of CD19-targeted T cells. Nature *558*, 307–312.

Gattinoni, L., Klebanoff, C.A., and Restifo, N.P. (2012). Paths to stemness: building the ultimate antitumour T cell. Nat. Rev. Cancer *12*, 671–684.

Grant, C.E., Bailey, T.L., and Noble, W.S. (2011). FIMO: scanning for occurrences of a given motif. Bioinformatics *27*, 1017–1018.

Gray, S.M., Amezquita, R.A., Guan, T., Kleinstein, S.H., and Kaech, S.M. (2017). Polycomb repressive complex 2-mediated chromatin repression guides effector $CD8^+$ T cell terminal differentiation and loss of multipotency. Immunity *46*, 596–608.

Gray, L.R., Sultana, M.R., Rauckhorst, A.J., Oonthonpan, L., Tompkins, S.C., Sharma, A., Fu, X., Miao, R., Pewa, A.D., Brown, K.S., et al. (2015). Hepatic mitochondrial pyruvate carrier 1 is required for efficient regulation of gluconeogenesis and whole-body glucose homeostasis. Cell Metab. *22*, 669–681.

Guo, Y., Xie, Y.Q., Gao, M., Zhao, Y., Franco, F., Wenes, M., Siddiqui, I., Bevilacqua, A., Wang, H., Yang, H., et al. (2021). Metabolic reprogramming of terminally exhausted CD8(+) T cells by IL-10 enhances anti-tumor immunity. Nat. Immunol. *22*, 746–756.

Halestrap, A.P. (1975). The mitochondrial pyruvate carrier. Kinetics and specificity for substrates and inhibitors. Biochem. J. *148*, 85–96.

He, B., Xing, S., Chen, C., Gao, P., Teng, L., Shan, Q., Gullicksrud, J.A., Martin, M.D., Yu, S., Harty, J.T., et al. (2016). $CD8^+$ T cells utilize highly dynamic enhancer repertoires and regulatory circuitry in response to infections. Immunity *45*, 1341–1354.

Heinz, S., Benner, C., Spann, N., Bertolino, E., Lin, Y.C., Laslo, P., Cheng, J.X., Murre, C., Singh, H., and Glass, C.K. (2010). Simple combinations of lineage-determining transcription factors prime cis-regulatory elements required for macrophage and B cell identities. Mol. Cell *38*, 576–589.

Herzig, S., Raemy, E., Montessuit, S., Veuthey, J.L., Zamboni, N., Westermann, B., Kunji, E.R., and Martinou, J.C. (2012). Identification and functional expression of the mitochondrial pyruvate carrier. Science *337*, 93–96.

Ho, P.C., Bihuniak, J.D., Macintyre, A.N., Staron, M., Liu, X., Amezquita, R., Tsui, Y.C., Cui, G., Micevic, G., Perales, J.C., et al. (2015). Phosphoenolpyruvate is a metabolic checkpoint of anti-tumor T cell responses. Cell *162*, 1217–1228.

Hui, S., Ghergurovich, J.M., Morscher, R.J., Jang, C., Teng, X., Lu, W., Esparza, L.A., Reya, T., Le Zhan, Z., Yanxiang Guo, J., et al. (2017). Glucose feeds the TCA cycle via circulating lactate. Nature *551*, 115–118.

Husain, Z., Huang, Y., Seth, P., and Sukhatme, V.P. (2013). Tumor-derived lactate modifies antitumor immune response: effect on myeloid-derived suppressor cells and NK cells. J. Immunol. *191*, 1486–1495.

Joshi, N.S., Cui, W., Chandele, A., Lee, H.K., Urso, D.R., Hagman, J., Gapin, L., and Kaech, S.M. (2007). Inflammation directs memory precursor and short-lived effector CD8(+) T cell fates via the graded expression of T-bet transcription factor. Immunity *27*, 281–295.

Kessner, D., Chambers, M., Burke, R., Agus, D., and Mallick, P. (2008). ProteoWizard: open source software for rapid proteomics tools development. Bioinformatics *24*, 2534–2536.

Klebanoff, C.A., Gattinoni, L., Palmer, D.C., Muranski, P., Ji, Y., Hinrichs, C.S., Borman, Z.A., Kerkar, S.P., Scott, C.D., Finkelstein, S.E., et al. (2011). Determinants of successful CD8+ T-cell adoptive immunotherapy for large established tumors in mice. Clin. Cancer Res. *17*, 5343–5352.

Klein Geltink, R.I., Edwards-Hicks, J., Apostolova, P., O'Sullivan, D., Sanin, D.E., Patterson, A.E., Puleston, D.J., Ligthart, N.A.M., Buescher, J.M., Grzes, K.M., et al. (2020). Metabolic conditioning of $CD8^+$ effector T cells for adoptive cell therapy. Nat. Metab. *2*, 703–716.

Knudson, K.M., Pritzl, C.J., Saxena, V., Altman, A., Daniels, M.A., and Teixeiro, E. (2017). NFkappaB-Pim-1-Eomesodermin axis is critical for maintaining CD8 T-cell memory quality. Proc. Natl. Acad. Sci. U. S. A. *114*, E1659–E1667.

Kong, W., Dimitri, A., Wang, W., Jung, I.Y., Ott, C.J., Fasolino, M., Wang, Y., Kulikovskaya, I., Gupta, M., Yoder, T., et al. (2021). BET bromodomain protein inhibition reverses chimeric antigen receptor extinction and reinvigorates exhausted T cells in chronic lymphocytic leukemia. J. Clin. Invest. *131*.

Langmead, B., and Salzberg, S.L. (2012). Fast gapped-read alignment with Bowtie 2. Nat. Methods *9*, 357–359.

Li, H., Handsaker, B., Wysoker, A., Fennell, T., Ruan, J., Homer, N., Marth, G., Abecasis, G., and Durbin, R.; 1000 Genome Project Data Processing Subgroup (2009). The Sequence Alignment/Map format and SAMtools. Bioinformatics *25*, 2078–2079.

Lund, P.J., Kori, Y., Zhao, X., Sidoli, S., Yuan, Z.F., and Garcia, B.A. (2019). Isotopic labeling and quantitative proteomics of acetylation on histones and beyond. Methods Mol. Biol. *1977*, 43–70.

Lunt, S.Y., Muralidhar, V., Hosios, A.M., Israelsen, W.J., Gui, D.Y., Newhouse, L., Ogrodzinski, M., Hecht, V., Xu, K., Acevedo, P.N., et al. (2015). Pyruvate kinase isoform expression alters nucleotide synthesis to impact cell proliferation. Mol. Cell *57*, 95–107.

Luo, M.C., Zhou, S.Y., Feng, D.Y., Xiao, J., Li, W.Y., Xu, C.D., Wang, H.Y., and Zhou, T. (2016). Runt-related transcription factor 1 (RUNX1) binds to p50 in macrophages and enhances TLR4-triggered inflammation and septic shock. J. Biol. Chem. *291*, 22011–22020.

Ma, E.H., Verway, M.J., Johnson, R.M., Roy, D.G., Steadman, M., Hayes, S., Williams, K.S., Sheldon, R.D., Samborska, B., Kosinski, P.A., et al. (2019). Metabolic profiling using stable isotope tracing reveals distinct patterns of glucose utilization by physiologically activated $CD8^+$ T cells. Immunity *51*, 856–870.e5.

Melamud, E., Vastag, L., and Rabinowitz, J.D. (2010). Metabolomic analysis and visualization engine for LC-MS data. Anal. Chem. *82*, 9818–9826.

Millard, P., Letisse, F., Sokol, S., and Portais, J.C. (2012). IsoCor: correcting MS data in isotope labeling experiments. Bioinformatics *28*, 1294–1296.

Miller, B.C., Sen, D.R., Al Abosy, R., Bi, K., Virkud, Y.V., LaFleur, M.W., Yates, K.B., Lako, A., Felt, K., Naik, G.S., et al. (2019). Subsets of exhausted $CD8^+$ T cells differentially mediate tumor control and respond to checkpoint blockade. Nat. Immunol. *20*, 326–336.

Milone, M.C., Fish, J.D., Carpenito, C., Carroll, R.G., Binder, G.K., Teachey, D., Samanta, M., Lakhal, M., Gloss, B., Danet-Desnoyers, G., et al. (2009).

Chimeric receptors containing CD137 signal transduction domains mediate enhanced survival of T cells and increased antileukemic efficacy in vivo. Mol. Ther. *17*, 1453–1464.

O'Sullivan, D., van der Windt, G.J., Huang, S.C., Curtis, J.D., Chang, C.H., Buck, M.D., Qiu, J., Smith, A.M., Lam, W.Y., DiPlato, L.M., et al. (2014). Memory $CD8^+$ T cells use cell-intrinsic lipolysis to support the metabolic programming necessary for development. Immunity *41*, 75–88.

Pais Ferreira, D., Silva, J.G., Wyss, T., Fuertes Marraco, S.A., Scarpellino, L., Charmoy, M., Maas, R., Siddiqui, I., Tang, L., Joyce, J.A., et al. (2020). Central memory $CD8^+$ T cells derive from stem-like $Tcf7^{hi}$ effector cells in the absence of cytotoxic differentiation. Immunity *53*, 985–1000.e11.

Quinlan, A.R., and Hall, I.M. (2010). BEDTools: a flexible suite of utilities for comparing genomic features. Bioinformatics *26*, 841–842.

Ramstead, A.G., Wallace, J.A., Lee, S.H., Bauer, K.M., Tang, W.W., Ekiz, H.A., Lane, T.E., Cluntun, A.A., Bettini, M.L., Round, J.L., et al. (2020). Mitochondrial pyruvate Carrier 1 promotes peripheral T cell homeostasis through metabolic regulation of thymic development. Cell Rep. *30*, 2889–2899.e6.

Ross-Innes, C.S., Stark, R., Teschendorff, A.E., Holmes, K.A., Ali, H.R., Dunning, M.J., Brown, G.D., Gojis, O., Ellis, I.O., Green, A.R., et al. (2012). Differential oestrogen receptor binding is associated with clinical outcome in breast cancer. Nature *481*, 389–393.

Rundqvist, H., Veliça, P., Barbieri, L., Gameiro, P.A., Bargiela, D., Gojkovic, M., Mijwel, S., Reitzner, S.M., Wulliman, D., Ahlstedt, E., et al. (2020). Cytotoxic T-cells mediate exercise-induced reductions in tumor growth. eLife *9*, e59996.

Saldanha, A.J. (2004). Java TreeView–extensible visualization of microarray data. Bioinformatics *20*, 3246–3248.

Schubert, M., Lindgreen, S., and Orlando, L. (2016). AdapterRemoval v2: rapid adapter trimming, identification, and read merging. BMC Res. Notes *9*, 88.

Siddiqui, I., Schaeuble, K., Chennupati, V., Fuertes Marraco, S.A., Calderon-Copete, S., Pais Ferreira, D., Carmona, S.J., Scarpellino, L., Gfeller, D., Pradervand, S., et al. (2019). Intratumoral $Tcf1^+PD\text{-}1^+CD8^+$ T cells with stem-like properties promote tumor control in response to vaccination and checkpoint blockade immunotherapy. Immunity *50*, 195–211.e110.

Sonveaux, P., Végran, F., Schroeder, T., Wergin, M.C., Verrax, J., Rabbani, Z.N., De Saedeleer, C.J., Kennedy, K.M., Diepart, C., Jordan, B.F., et al. (2008). Targeting lactate-fueled respiration selectively kills hypoxic tumor cells in mice. J. Clin. Invest. *118*, 3930–3942.

Sturm, M., Bertsch, A., Gröpl, C., Hildebrandt, A., Hussong, R., Lange, E., Pfeifer, N., Schulz-Trieglaff, O., Zerck, A., Reinert, K., and Kohlbacher, O. (2008). OpenMS - an open-source software framework for mass spectrometry. BMC Bioinformatics *9*, 163.

Sukumar, M., Liu, J., Ji, Y., Subramanian, M., Crompton, J.G., Yu, Z., Roychoudhuri, R., Palmer, D.C., Muranski, P., Karoly, E.D., et al. (2013). Inhibiting glycolytic metabolism enhances CD8+ T cell memory and antitumor function. J. Clin. Invest. *123*, 4479–4488.

Teoh, S.T., Ogrodzinski, M.P., Ross, C., Hunter, K.W., and Lunt, S.Y. (2018). Sialic acid metabolism: a key player in breast cancer metastasis revealed by metabolomics. Front. Oncol. *8*, 174.

Tschumi, B.O., Dumauthioz, N., Marti, B., Zhang, L., Lanitis, E., Irving, M., Schneider, P., Mach, J.P., Coukos, G., Romero, P., and Donda, A. (2018). CART cells are prone to Fas- and DR5-mediated cell death. J. Immunother. Cancer *6*, 71.

Vacanti, N.M., Divakaruni, A.S., Green, C.R., Parker, S.J., Henry, R.R., Ciaraldi, T.P., Murphy, A.N., and Metallo, C.M. (2014). Regulation of substrate utilization by the mitochondrial pyruvate carrier. Mol. Cell *56*, 425–435.

van der Veeken, J., Zhong, Y., Sharma, R., Mazutis, L., Dao, P., Pe'er, D., Leslie, C.S., and Rudensky, A.Y. (2019). Natural genetic variation reveals key features of epigenetic and transcriptional memory in virus-specific CD8 T cells. Immunity *50*, 1202–1217.e7.

Wang, R., Dillon, C.P., Shi, L.Z., Milasta, S., Carter, R., Finkelstein, D., McCormick, L.L., Fitzgerald, P., Chi, H., Munger, J., and Green, D.R. (2011). The transcription factor Myc controls metabolic reprogramming upon T lymphocyte activation. Immunity *35*, 871–882.

Wang, D., Diao, H., Getzler, A.J., Rogal, W., Frederick, M.A., Milner, J., Yu, B., Crotty, S., Goldrath, A.W., and Pipkin, M.E. (2018). The transcription factor Runx3 establishes chromatin accessibility of cis-regulatory landscapes that drive memory cytotoxic T lymphocyte formation. Immunity *48*, 659–674.e6.

Watson, M.J., Vignali, P.D.A., Mullett, S.J., Overacre-Delgoffe, A.E., Peralta, R.M., Grebinoski, S., Menk, A.V., Rittenhouse, N.L., DePeaux, K., Whetstone, R.D., et al. (2021). Metabolic support of tumour-infiltrating regulatory T cells by lactic acid. Nature *591*, 645–651.

Wörn, A., and Plückthun, A. (1998). An intrinsically stable antibody scFv fragment can tolerate the loss of both disulfide bonds and fold correctly. FEBS Lett. *427*, 357–361.

Wu, T., Hu, E., Xu, S., Chen, M., Guo, P., Dai, Z., Feng, T., Zhou, L., Tang, W., Zhan, L., et al. (2021). clusterProfiler 4.0: a universal enrichment tool for interpreting omics data. Innovation (N Y) *2*, 100141.

Yang, C., Ko, B., Hensley, C.T., Jiang, L., Wasti, A.T., Kim, J., Sudderth, J., Calvaruso, M.A., Lumata, L., Mitsche, M., et al. (2014). Glutamine oxidation maintains the TCA cycle and cell survival during impaired mitochondrial pyruvate transport. Mol. Cell *56*, 414–424.

Yu, B., Zhang, K., Milner, J.J., Toma, C., Chen, R., Scott-Browne, J.P., Pereira, R.M., Crotty, S., Chang, J.T., Pipkin, M.E., et al. (2017). Epigenetic landscapes reveal transcription factors that regulate $CD8^+$ T cell differentiation. Nat. Immunol. *18*, 573–582.

Yu, Y.R., Imrichova, H., Wang, H., Chao, T., Xiao, Z., Gao, M., Rincon-Restrepo, M., Franco, F., Genolet, R., Cheng, W.C., et al. (2020). Disturbed mitochondrial dynamics in $CD8^+$ TILs reinforce T cell exhaustion. Nat. Immunol. *21*, 1540–1551.

Zhang, Y., Liu, T., Meyer, C.A., Eeckhoute, J., Johnson, D.S., Bernstein, B.E., Nusbaum, C., Myers, R.M., Brown, M., Li, W., and Liu, X.S. (2008). Model-based analysis of ChIP-seq (MACS). Genome Biol. *9*, R137.

Zhu, L.J., Gazin, C., Lawson, N.D., Pagès, H., Lin, S.M., Lapointe, D.S., and Green, M.R. (2010). ChIPpeakAnno: a Bioconductor package to annotate ChIP-seq and ChIP-chip data. BMC Bioinformatics *11*, 237.

STAR★METHODS

KEY RESOURCES TABLE

REAGENT or RESOURCE	SOURCE	IDENTIFIER
Antibodies		
Live/dead fixable Aqua Dead cell stain kit	Thermo Fisher Scientific	Lifetechnologies
anti-mouse CD8a - PE-TexasRed	eBioscience / BioLegend	RRID: AB_10374589
anti-mouse CD62L - FITC, PerCP-Cy5.5, BV711	Thermo Fisher Scientific	RRID: AB_465109
anti-mouse CD44 - APC-Cy7	in house	N/A
anti-mouse CD127 - APC, PE	eBioscience /in house	Clone A7R34; RRID: AB_469435; RRID: AB_465845
anti-mouse KLRG1 - PE-Cy7	Biolegend	RRID: AB_2561736
anti-mouse TCF1/ TCF7 (C63D9) Rabbit mAb	Cell Signaling	RRID: AB_2199302
anti-mouse CD45.1 - PerCP-Cy5.5, PE	eBioscience	RRID: AB_2534309
anti-mouse CD45.2 - APC-Cy7, Pacific Blue	in house	N/A
anti-mouse Thy1.1 (CD90.1) - PE, APC	Thermo Fisher Scientific	RRID: AB_2534975; RRID: AB_2535002
anti-mouse IL2 - PE-Cy7	Biolegend	RRID: AB_2561749
anti-mouse IFNg - PerCP-Cy5.5	Thermo Fisher Scientific	RRID: AB_906239
anti-mouse TNFa - Pacific Blue	Biolegend	RRID: AB_893639
anti-mouse CD107a (LAMP-1) - PE	BD	RRID: AB_1645247
anti-human granzyme B - PE-TexasRed	Invitrogen	RRID: AB_2536540
anti-mouse PD1 (CD279) - APC, PerCP-Cy5.5	Biolegend	RRID: AB_10550092; RRID: AB_10613470
anti-human TIM3 (CD366) - Pacific Blue	Biolegend	RRID: AB_2632853
anti-mouse LAG3 (CD223) - PE	Biolegend	RRID: AB_2133343
anti-human CD3 - BV711	Biolegend	RRID: AB_11219592
anti-human CD8 - BV421	Biolegend	RRID: AB_2629583
anti-human CD4 - AF532	Thermo Fisher Scientific	RRID: AB_11218891
anti-human CD45RA - BUV395	BD	HI100
anti-human CD45RO - BUV805	BD	RRID: AB_2872786
anti-human CD62L - PerCP-Cy5.5	Biolegend	RRID: AB_893396
anti-human CCR7 (CD197) - Pacific Blue	Biolegend	RRID: AB_10915695
anti-human CD95 - PE	Thermo Fisher Scientific	RRID: AB_465788
anti-human CD27 - BV605	Biolegend	RRID: AB_2561450
anti-human CD28 - PE-Cy7	Biolegend	RRID: AB_10644005
recombinant human Fc-tagged Her2/ErbB2 protein	Sino Biological	Cat# 10004-H02H
anti-human-Fc Alexa Fluor 488 conjugate	Thermo Fisher Scientific	RRID: AB_2534050
anti-human CD19	Biolegend	RRID: AB_314238
HRP-labeled secondary anti-rabbit	Santa Cruz Biotechnology	RRID: AB_1500696
HRP-labeled secondary anti-mouse	Santa Cruz Biotechnology	RRID: AB_2536527
Acetyl-Histone H3 (Lys27) Rabbit mAb	Cell Signaling	RRID: AB_10949503
Tri-Methyl-Histone H3 (Lys27) (C36B11) Rabbit mAb	Cell Signaling	RRID: AB_2616029
Histone H3 Antibody	Cell Signaling	RRID: AB_331563
anti-RUNX1 antibody	GeneTex	RRID: AB_2885895
Phospho-S6 Ribosomal Protein (Ser235/236) (D57.2.2E) XP Rabbit mAb	Bioconcept	RRID: AB_916156
4E-BP1 (phospho Thr37/Thr46) antibody 4E-BP1 (phospho Thr37/Thr46) antibody	GeneTex	RRID: AB_2886856

(*Continued on next page*)

Continued

REAGENT or RESOURCE	SOURCE	IDENTIFIER
Monoclonal Anti-b-Actin antibody	Sigma-Aldrich	RRID: AB_476697
a-Tubulin (DM1A) Mouse	Cell Signaling	RRID: AB_1904178
Acetylated lysine antibody	Cell Signaling	RRID: AB_331805
Annexin V PE	Biolegend	Cat# 640907
**Bacterial and virus strains**		
Recombinant bacteria Listeria monocytogenes deficient for actA and expressing the ovalbumin (Ova) peptide SIINFEKL	In house	N/A
**Chemicals, peptides, and recombinant proteins**		
UK-5099	Sigma-Aldrich	Cat# PZ0160
DMSO	Sigma-Aldrich	Cat# 34869
LDHA inhibitor GSK 2837808A	ThermoFisher Scientific	Cat# 51-891-0
Torin2	Sigma-Aldrich	Cat# SML1224
RBC Lysis solution	Qiagen	Cat# 158904
Golgi Plug	BD	Cat# 51-2301KZ
Golgi Stop	BD	Cat# 554724
Trypan Blue Stain 0.4 %	Invitrogen	Cat# 15250061
Fibronectin	Takara Clontech	Cat# T100A
Bovine Serum Albumin	Sigma-Aldrich	Cat# A2153
Cyclophosphamide	Sigma-Aldrich	Cat# C7397
Propidium iodide	Biolegend	Cat# 421301
Peptide Ovalbumin amino acids 257-264 (OVA) (SIINFEKL)	In house	N/A
Percoll	GE Heathcare	Cat# 17-0891-01
Recombinant human IL-2	Peprotech	Cat# 200-02
Recombinant human IL-7	Peprotech	Cat# 200-07
Recombinant human IL-15	Peprotech	Cat# 210-15
Lymphoprep	Axonlab	Cat# 1114545
CpG-ODN 1826 oligonucleotide	Microsynth	Cat# 45355
U-^{13}C-L-Lactate	Sigma-Aldrich	Cat# 485926
U-^{13}C-glucose	Cambridge Isotope Laboratory	Cat# CLM-1396-1
U-^{13}C-glutamine	Cambridge Isotope Laboratory	Cat# CLM-1822-H-0.1
U-^{13}C-palmitate	Sigma-Aldrich	Cat# 605751-SPEC
4-(2-Aminoethyl)benzenesulfonyl fluoride hydrochloride	Sigma-Aldrich	Cat# A8456
Phosstop	Sigma-Aldrich	Cat# 4906845001
Nitrocellulose membranes 0.2 μm	Biorad	Cat# 1620150
Recombinant Protein L, biotinylated	Pierce	Cat# 29997
**Critical commercial assays**		
Tumor Dissociation Kit	Miltenyi Biotec	Cat# 130-096-730
Mouse CD8+ T cell enrichment kit	StemCell Technologies	Cat# 19853
Mouse naive CD8+ T cell enrichment kit	StemCell Technologies	Cat# 19858
Mouse CD90.1 Positive Selection Kit	StemCell Technologies	Cat# 18958
Intracellular Fix & Perm Buffer set	eBiosciences	Cat# 88-8824
FoxP3/Transcription factor staining	eBiosciences	Cat# 00-5523
Annexin V - APC Apoptosis Detection Kit	eBioscience	RRID: AB_2575165
SuperScript III First-Strand Synthesis System	ThermoFisher Scientific	Cat# 18080051
KAPA SYBR FAST qPCR Kit Master Mix	Kapabiosystems	Cat# KR0389
Dynabeads Mouse T-Activator CD3/CD28	ThermoFisher Scientific	Cat# 11452D

(*Continued on next page*)

Continued

REAGENT or RESOURCE	SOURCE	IDENTIFIER
Dynabeads Human T-Activator CD3/CD28	ThermoFisher Scientific	Cat# 11161D
MinElute PCR Purification kit	QIAGEN	Cat# 28004
CountBright Plus Absolute Counting Beads	ThermoFisher Scientific	Cat# C36995
PureLink HiPure Plasmid Filter Midiprep Kit	ThermoFisher Scientific	Cat# K210014
StraightFrom BuffyCoat REALease CD3 microbeads	Miltenyi	Cat# 130-127-142
BCA Protein Assay Kit	ThermoFisher Scientific	Cat# 10678484
Transposase reaction mix	Illumina	Cat# 20034197
Agencourt AMPure XP magnetic beads	Beckman	Cat# A63880
ECL reagents	Super Signal West, Thermo Scientific	Cat# RPN2235
Femto reagents	Super Signal West, Thermo Scientific	Cat# 34096
Deposited data		
ATAC-seq data	This paper	GEO: GSE184718
Raw data and uncropped scans of Western blots	This paper	Data S1
Experimental models: Cell lines		
B16-F10 melanoma	American Type Culture Collection	N/A
Phoenix-Eco	American Type Culture Collection	N/A
the human B cell leukemia NALM6	American Type Culture Collection	N/A
Experimental models: Organisms/strains		
Mouse: C57BL/6 (B6) (CD45.2)	Charles River	N/A
Mouse: C57BL/6 (B6) (CD45.1)	Janvier	N/A
Mouse: Cd4-Cre (B6.Cg-Tg(Cd4-cre) 1Cwi/BfluJ)	Jackson Laboratory	Jax: 22071
Mouse: whole body Cas9 (B6J.129(Cg)-Gt(ROSA)26Sortm1.1(CAG-cas9*,-EGFP) Fezh/J)	Jackson Laboratory	Jax: 026179
Mouse: B6;129-Gt(ROSA)26Sortm1 (CAG-cas9*,-EGFP)Fezh/J)	Jackson Laboratory	Jax: 024857
Mouse: OT1 C57BL/6-Tg(TcraTcrb)1100Mjb/J	Jackson Laboratory	Jax: 003831
Mouse: NOD.Cg-Prkdcscid Il2rgtm1Wjl/SzJ (NOD scid g, NSG)	Jackson Laboratory	Jax: 005557
Mouse: Mpc1tm1a(EUCOMM)Wtsi	Gray et al. (2015)	N/A
Oligonucleotides		
Runx1 sequence 1	Doench et al. (2016)	CGGTCCCTACACTAGGACAT
Runx1 sequence 2	Doench et al. (2016)	TGCGCACTAGCTCGCCAGGG
Runx1 sequence 3	Doench et al. (2016)	CCAGCGACACCCATTTCACC
Software and algorithms		
GraphPad Prism v9	http://graphpad-prism.software.informer.com/5.0/	N/A
FlowJo v10	Tree Star	N/A
ProteoWizard	Kessner et al. (2008)	N/A
OpenMS	Sturm et al. (2008)	N/A
MAVEN	Melamud et al. (2010)	N/A
IsoCor	Millard et al. (2012)	N/A
Cluster 3.0	de Hoon et al. (2004)	N/A
Java Treeview	Saldanha (2004)	N/A
Mascot 2.7	Matrix Science, London, UK	N/A
AdapterRemoval v. 2.1.7	Schubert et al. (2016)	N/A
Bowtie 2 v. 2.3.4.1	Langmead and Salzberg (2012)	N/A
samtools v. 1.8	Li et al. (2009)	N/A
MACS2 v. 2.1.1.20160309	Zhang et al. (2008)	N/A
R v. 3.5.1	https://www.R-project.org/	N/A

(*Continued on next page*)

Continued

REAGENT or RESOURCE	SOURCE	IDENTIFIER
DiffBind package v. 2.10.0	Ross-Innes et al. (2012); http://bioconductor.org/packages/release/bioc/vignettes/DiffBind/inst/doc/DiffBind.pdf	N/A
ChIPpeakAnno package v. 3.16.1	Zhu et al. (2010)	N/A
Homer software v. 4.11	Heinz et al. (2010)	N/A
BEDtools suite	Quinlan and Hall (2010)	N/A
Jaspar database	http://jaspar.genereg.net/	N/A
FIMO program	Grant et al. (2011)	N/A
MEME suite	https://meme-suite.org/meme/doc/fimo.html?man_type=web	N/A
clusterProfiler package v. 4.0.4	Wu et al. (2021)	N/A

RESOURCE AVAILABILITY

Lead contact

Further information and requests for resources and reagents should be directed to and will be fulfilled by the lead contact, Mathias Wenes (mathias.wenes@unige.ch).

Materials availability

This study did not generate new unique reagents.

Data and code availability

- The raw ATAC sequencing data reported in this paper has been deposited in NCBI's Gene Expression Omnibus and are accessible through GEO: GSE184718 (https://www.ncbi.nlm.nih.gov/geo/query/acc.cgi?acc=GSE184718).
- The R script used for differential accessibility analysis is available at https://github.com/taniawyss/ATACseq_mouse_T_cells_DMSO_vs_UK5099_MW.
- Uncropped scans of all Western blots and all raw data used to create all graphs can be found in Data S1.
- Any additional information required to reanalyse the data reported in this paper is available from the lead contact upon request.

EXPERIMENTAL MODEL AND SUBJECT DETAILS

Mice

C57BL/6 (B6) (CD45.2) mice were obtained from Charles River (France) and intercrossed with B6 (CD45.1) mice, obtained from Janvier, to generate CD45.1.2 mice. *Cd4-Cre* (B6.Cg-Tg(Cd4-cre)1Cwi/BfluJ), whole body Cas9 (B6J.129(Cg)- *Gt(ROSA)26Sor*$^{tm1.1(CAG\text{-}cas9*,\text{-}EGFP)Fezh}$/J) and conditional Cas9 or Rosa26-LSL-Cas9 knock-in (B6;129-*Gt(ROSA)26Sor*$^{tm1(CAG\text{-}cas9*,\text{-}EGFP)Fezh}$/J) mice were purchased from the Jackson Laboratory and bred in-house. OT1 T cell receptor (TCR) transgenic mice, expressing a TCR specific for the chicken egg ovalbumin SIINFEKL epitope in the context of $H2K^b$, and NOD.Cg-*Prkdc*^{scid} *Il2rg*^{tm1Wjl}/*SzJ* (NOD *SCID*-γ, NSG) mice were bred in-house. CRISPR–Cas9 knock-in OT1 TCR-transgenic mice were obtained by crossing of Rosa26-LSL-Cas9 knock-in mice with *Cd4-Cre* mice on an OT1 background. *Mpc1*$^{fl/fl}$ mice (Mpc1$^{tm1a(EUCOMM)Wtsi}$) were obtained from Jean-Claude Martinou (University of Geneva) with permission from Jared Rutter (University of Utah School of Medicine). *Mpc1*$^{fl/fl}$ mice were crossed to *Cd4-Cre* mice on an OT-I background. Mouse strains were maintained in the SPF animal facility of the University of Lausanne. Experiments used both male and female mice between 6 and 10 weeks of age whereby donors and recipients of adoptive T cell transfers were sex matched. Mice were housed at 22°C with 55% relative humidity on a 12h/12h light/dark cycle and were fed Safe-150 chow (Safe, cat#: U8404G10R). Health status was checked every 3 months following FELASA guidelines. Animal experiments were conducted in accordance with protocols approved by the veterinary authorities of the Canton de Vaud (VD2688.2).

Primary cell culture

Mouse T cells were freshly isolated from spleens of both male and female mice between 6 and 10 weeks of age.

For human studies, T cells were isolated from heparinized blood from healthy male and female volunteers aged between 25 and 65 years, or for CD19-CAR manufacture, buffy coats that were provided by Transfusion Interrégional CRS (Bern, Switzerland) (anonymized). Donors provided written consent.

Cell lines

B16-F10 melanoma cells, Phoenix-Eco cells and the human B cell leukemia cell line NALM6 cells were originally acquired from the American Type Culture Collection. B16 cells were cultured in DMEM, supplemented with 10% fetal bovine serum

(FBS), 1% Penicillin/Streptomycin (P/S, Gibco 15070-063), and 1% L-glutamine (Q, Gibco 25030-081). Phoenix-Eco cells were cultured in IMDM, supplemented with 10% FBS and 1% P/S. Nalm6 cells were cultured in RPMI-1640 media supplemented with 10% FBS, 1% P/S and 10mM HEPES. All cells were cultured in a humidified incubator at 37°C with 5% CO_2.

Cell lines were systematically tested and proven mycoplasma-free, by polymerase chain reaction (PCR) testing.

METHOD DETAILS

Mouse T cell culture

Spleens were collected under sterile conditions. The spleen was smashed on a 70 μm cell strainer and single cells were collected by centrifugation. Red blood cells were lysed by Red Blood Cell Lysis Buffer (Qiagen). Splenocytes were counted and seeded at a concentration of 10^6 cells per mL in RPMI medium (Gibco 61870-01) supplemented with 10% FBS, (Gibco 10270-106), 1% Penicillin/Streptomycin (Gibco 15070-063), 50μM ß-mercaptoethanol, 10mM HEPES (Gibco 15630-080), 1x Non-essential amino acids (Gibco 11140-035), 1% L-glutamine (Gibco 25030-081) and 1mM Sodium Pyruvate (Gibco 11360-039). Cells were additionally supplemented with recombinant human (rh) IL-2 100IU/ml (in house), ovalbumin peptide (SIINFEKL) 1μg/ml and either with 20 μM UK5099 (Sigma Aldrich) or with their solvent DMSO as a control. For adoptive cell transfer, splenocytes were collected at day 3, washed and split, and cells were cultured for 4 additional days with 100IU/ml rhIL-2 and hIL-7 (Peprotech 200-07) supplemented either with 20 μM UK5099 (Sigma Aldrich) or DMSO. For ATAC-seq analysis (see below), splenocytes were collected at day 3, washed and split, and cells were cultured for 4 additional days with 100U/ml rhIL-2 and 10ng/ml rhIL-7 (Peprotech 200-07) without small molecule inhibitor.

For *in vitro* tumor nutrient modelling experiments, WT or MPC1 KO OT1 splenocytes were activated with SIINFEKL and 100 IU/ml rhIL2. Cells were washed and split after 3 days, and then cultured for another 3 days with 100U/ml rhIL-2 and 10ng/ml rhIL-7. The T cells were then collected, counted and seeded at 10^6 cells/ml in glucose- and glutamine-free RPMI (biological industries) supplemented with 10% dialyzed FBS (Thermo Fisher Scientific) and either 11mM or 0.5mM D-glucose (Sigma), 4mM or 0.1mM L-glutamine (Gibco 25030-081), 1% Penicillin/Streptomycin (Gibco 15070-063), 50μM ß-mercaptoethanol, 1% HEPES (Gibco 15630-080), and the indicated concentrations of sodium L-lactate, or sodium chloride (Sigma) as osmotic control for 18 hours at 37°C and then restimulated with 1μg/ml SIINFEKL for 4 hours by adding concentrated peptide at <10% of the original culture medium.

Preparation of mouse CAR T cells

BFP fluorescent protein and HER2 CAR were cloned in the MSGV retroviral transfer vector (Coren et al., 2015) under the control of the 5′ LTR promoter as described previously (Tschumi et al., 2018). Briefly, for the HER2-CAR, the plasmid pIG6-4D5, containing the scFv fragment derived from the human-specific anti-HER2 murine antibody 4D5, was used as template (Wörn and Plückthun, 1998) (kind gift from A. Pluckthun, University of Zurich, Switzerland). The single chain antibody fragment was fused to the CD8α hinge and transmembrane domains followed by mouse intracellular 4-1BB and CD3ζ signaling endodomains. Phoenix-Eco cells were transfected with HER2 CAR or BFP plasmid and pCL-Eco-packaging plasmid using the calcium phosphate method. Virus-containing supernatant was collected by ultracentrifugation after 48h and 72h. Spleens from wild type CD45.1.2 mice were smashed on a 70μm cell strainer. $CD8^+$ T cells were purified using the EasySepTM Mouse $CD8^+$ T Cell Isolation Kit (StemCell) according to the manufacturer protocol. 0.5x10^6 $CD8^+$ T cells were plated in 48-well plates in 0.5 ml of complete RPMI 1640 medium supplemented with 10% FCS, antibiotics and 50 IU/ml of recombinant human IL-2, and exposed to either DMSO or 20μM UK5099. Mouse T-cells were activated with Activator CD3/CD28 Dynabeads (Gibco) at a ratio of 2 beads per cell. Retroviral infection was conducted at 37°C for 24h. Untreated 48-well plates were coated for 24h with 20 μg/ml of recombinant human fibronectin (Takara Clontech) at 4°C, followed by PBS 2% BSA for 30 min at RT and finally washed with PBS. Concentrated retroviruses were plated in each fibronectin-coated 48-well plate and centrifuged for 90 min at 2000rcf and 32°C. Then, 0.5x10^6 of 24h-activated $CD8^+$ T cells were added on top of the viruses and spun for 10 min at 400rcf and 32°C. On day 3, the medium was replaced with 10 IU/ml recombinant human IL-2, 10 ng/ml recombinant human IL-7 and 10 ng/ml recombinant human IL-15, containing either DMSO or 20μM UK5099. Cells were then split every second day.

Human T cell culture

Bulk PBMCs (peripheral blood mononuclear cells) were obtained by density centrifugation from fresh peripheral blood from healthy donors. PBMCs (10^6 cells/ml) were seeded in 96-well plates in RPMI 1640 (Gibco, 61870-010) supplemented with 8% AB serum, kanamycin 1x (Gibco, 11360-039), 1% L-Glutamine (Gibco, 25030-024), 1% Non-essential amino acids (Gibco, 11140-035), 1% Na-Pyruvate (Gibco, 11360-039), 0,1% 2-mercaptoethanol (5x10^{-2} stock, Sigma, M-7522) and 150IU/ml rhIL-2 (Peprotech, 200-02) and activated with Dynabeads Human T-Activator CD3/CD28 (ThermoFisher, 11161D) at a 1:1 cell:bead ratio in the presence of 25μM UK5099. As negative control, the PBMCs were cultured in the solvent, Dimethyl Sulfoxide (DMSO, Sigma-Aldrich, 20-139). Cells were expanded at 10^6 cells/ml adding supplemented media. After 5 days of activation the beads were removed and cells were maintained in the media described in presence of UK5099 or DMSO. Cells were characterized by flow cytometry and expansion was determined after 9 days of activation.

Preparation of human CAR T cells

The hCD19-28z CAR was constructed by ligating the hCD19 scFv (FMC63) into the CAR backbone sequences of a third generation viral vector pTRPE-28z. Human CD3-selected T cells were purified from a donor Buffy Coat using StraightFrom BuffyCoat REALease

CD3 microbeads (Miltenyi) and activated with Dynabeads Human T-Activator CD3/CD28 (ThermoFisher, 11161D) at a 1:1 cell:bead ratio in the presence of 30IU/ml recombinant human IL2 (Peprotech, 200-02). 24 hours later, T cells were transduced with the lentiviral vector encoding anti-human CD19scFv fused to the CAR backbone containing human CD28 and CD3zeta (CD247) signaling domains, and were expanded *ex vivo* for 12 days. Transduced T cells were maintained at a concentration of 0.75 x 10^6 cells/ml throughout the culture period by cell enumeration every 2-3 days (Milone et al., 2009). The transduction efficiencies were near 80% (Figure S7D). After 12 days, cells were frozen in aliquots and stored in liquid nitrogen. T cells were exposed to 25μM UK5099 or DMSO over the entire culture period.

Listeria model of infection

Naive $CD8^+$ T cells were isolated from spleens (Stem Cell Technologies, catalogue number 19858) or splenocytes were cultured *in vitro* for 7 days as described above, collected and purified on a Ficoll gradient, allowing to separate dead and live splenocytes. For RUNX1 experiments, transduced T cells were isolated based on their Thy1.1 expression (Stem Cell Technologies). Live T cells were counted with Trypan blue stain 0.4%. 10^4 (RUNX1) or 10^5 (naive $CD8^+$ or cultured $CD8^+$ T cells) live T cells were transferred into host mice (B6 for naive $CD8^+$ or cultured $CD8^+$ T cells, whole body Cas9 for RUNX1) by tail vein injection.

Recombinant bacteria *Listeria monocytogenes* deficient for actA and expressing the ovalbumin (Ova) peptide SIINFEKL were expanded and tittered. Optical density measured with a spectrophotometer was used to determine bacterial concentration and 2000 colony-forming units were administered in each mouse by tail vein injection, 4 hours after adoptive cell transfer. Blood samples were collected at indicated times, by tail vein blood sampling.

Tumor models

C57BL/6 mice were engrafted subcutaneously with either 10^5 SIINFEKL-expressing or 4x10^5 HER2-expressing B16-F10 tumor cells. Six days later, mice were lymphodepleted by whole body irradiation with 5 Gray (RS2000, Rad Source, for B16-SIINFEKL), or with 100 mg/kg cyclophosphamide injected intraperitoneally, (Sigma Aldrich, C7397, for B16-HER2). Homogeneous groups were constituted with regard to tumor volume before intravenous adoptive cell transfer of 10^5 naive WT or MPC1 KO $CD8^+$ T cells, 2x10^5 DMSO- or UK5099-conditioned OT1 cells, or 5x10^6 BFP or HER2-CAR T cells. Tumor volumes were measured three times a week with a caliper and calculated using the formula: $V = \pi \times [d2 \times D] / 6$, where d is the minor tumor axis and D is the major tumor axis. At dissection, tumors were collected and separated from skin. Single cell suspensions were obtained with the Mouse Tumor Dissociation Kit (Miltenyi, 130–096-730) according to the manufacturer protocol. Spleen and draining lymph node were smashed on a 70 μm cell strainer. Single cell suspensions were stained with antibodies before flow cytometry analysis.

For the human leukemia mode, NSG mice were inoculated with 10^6 Nalm6 cells in the tail vein. 15 days after NALM6 infusion, human CAR T cells were thawed, washed and 2.5 x 10^6 live cells were adoptively transferred in the tail vein. The body weight was frequently measured and health of mice was monitored. When the physical and behaviour health of mice declined below the levels established by the Swiss cantonal authorities or body weight dropped >15%, mice were sacrificed. NALM6 cell numbers in the blood were measured by anti-human CD19 flow cytometry analysis.

^{13}C-labeling and LC-MS/MS metabolomics

Polyclonal naive $CD8^+$ T cells were activated on plates coated with 1μg/ml anti-CD3ε (Biolegend) in RPMI completed as described above, with 0.5 μg/ml soluble anti-CD28 (Biolegend) and DMSO or 75μM UK5099. For stable isotope labelling, media was switched after 66 hours to in glucose- and glutamine-free RPMI (biological industries) supplemented with 10% dialyzed FBS (Thermo Fisher Scientific) 1% Penicillin/Streptomycin, 50μM ß-mercaptoethanol, 1% HEPES, 1x Non-essential amino acids, containing 11mM U-^{13}C-glucose-, 4mM U-^{13}C-glutamine or 200μM ^{13}C-palmitate (Sigma) with and DMSO or 75μM UK5099, for 6 hours. For lactate incorporation, WT or MPC1 KO T cells were activated and placed in nutrient-deprived medium as described above containing 20mM U-^{13}C-L-Lactate (Sigma). Cells were pelleted by centrifugation (500 ×g, 4°C, 5 min), washed once with saline, and immediately flash-frozen in liquid nitrogen and stored at -80°C. To extract metabolites, cold 500 μL HPLC-grade methanol (-20°C) was added to each sample, vortexed briefly, followed by addition of 200 μL HPLC-grade water, then 500 μL HPLC-grade chloroform (final methanol:water:chloroform ratio 5:2:5). The mixture was vortexed at 4°C for 10 min, then centrifuged (16000 ×g, 4°C, 10 min) to achieve phase separation. The aqueous upper phase containing polar metabolites was separated and dried under a stream of nitrogen gas. The dried metabolite samples were stored at -80°C. The protein layer was dried in a fume hood then dissolved in 0.2 mM KOH overnight, then quantified using Pierce BCA Protein Assay Kit (Thermo Fisher).

For LC-MS/MS analysis, dried metabolite extracts were reconstituted in HPLC-grade water containing 1 μM piperazine-N,N′-bis(2-ethanesulfonic acid) (PIPES) as internal standard, at volumes corresponding to BCA protein quantification values. 20μL of reconstituted sample was added to 80μL methanol and derivatized with 10uL triethylamine and 2μL benzylchloroformate for amino acid analysis. Samples with and without derivatization were transferred to HPLC vials for analysis. LC-MS/MS analysis was performed with ion-pairing reverse phase chromatography using an Ascentis Express column (C18, 5 cm x 2.1 mm, 2.7 μm, Sigma) and a Waters Xevo TQ-S triple quadrupole mass spectrometer. The LC solvents were 10 mM tributylamine and 15 mM acetic acid in 97:3 water:methanol (Solvent A), and methanol (Solvent B). LC and MS parameters were as previously reported (Lunt et al., 2015; Teoh et al., 2018). Briefly, Elution from the column was performed over 12 min with the following gradient: t = 0, 0% solvent B, flow rate 0.4 ml/min; t = 1, 0% solvent B, flow rate 0.4 ml/min; t = 2, 20% solvent B, flow rate 0.3 ml/min; t = 3, 20% solvent B, flow rate 0.25 ml/min; t = 5, 55% solvent B, flow rate 0.15 ml/min; t = 8, 95% solvent B, flow rate 0.15 ml/min; t = 9.5, 95% solvent

B, flow rate 0.15 ml/min; t = 10, 0% solvent B, flow rate 0.4 ml/min; t = 12, 0% solvent B, flow rate 0.4 ml/min. Mass spectra were acquired using negative-mode electrospray ionization operating in multiple reaction monitoring (MRM) mode. The capillary voltage was 3,000 V, and cone voltage was 50 V. Nitrogen was used as cone gas and desolvation gas, with flow rates of 150 and 600 l/h, respectively. The source temperature was 150°C, and desolvation temperature was 500°C. Argon was used as collision gas at a manifold pressure of 4.3 × 10−3 mbar. Precursor and product ion m/z, collision energies and source cone potentials were optimized for each transition using Waters QuanOptimize software. The MRM transitions are as listed in Table S1. RAW data folders were converted to mzXML using ProteoWizard (Kessner et al., 2008) and OpenMS (Sturm et al., 2008). Peak quantification was performed in MAVEN (Melamud et al., 2010). and data for each sample was normalized to PIPES peak intensity. For isotopic ratios, natural isotope abundance correction was performed using IsoCor (Millard et al., 2012). Heatmaps were generated using Cluster 3.0 (de Hoon et al., 2004) and exported using Java Treeview (Saldanha, 2004).

Histone 13C acetylation

Sample preparation

OT1 splenocytes were activated with DMSO or UK5099 as described above. After 48 hours, the medium was replaced with glucose- and glutamine-free RPMI (biological industries) supplemented with 10% dialyzed FBS (Thermo Fisher Scientific) 1% Penicillin/Streptomycin, 50μM ß-mercaptoethanol, 1% HEPES, 1x Non-essential amino acids, containing 11mM U-^{13}C-glucose or 4mM U-^{13}C-glutamine, with and DMSO or UK5099, for 24 hours before cell collection. Histone isolation, derivatization and digestion were adapted from Lund et al. (2019). Briefly, nuclei were isolated and histones were acid extracted with H_2SO_4, and then precipitated with trichloroacetic acid. Samples were redissolved in 75μl Hepes 200mM, pH 8.5 buffer, and aliquots run on a SDS-PAGE 13% gel with quantitation standards. Based on gel results, concentration of samples was adjusted for the derivatization reaction. About 10 μg of sample in 20 μl Hepes buffer were incubated for 20 min at 37°C with 10 μl 25% propionic anhydride in 2-propanol. After the first 5 min of reaction, 10 μl of ammonium bicarbonate 1.0 M and 5 μl of propionic anhydride solution were added. After propionylation, samples were snap-frozen with liquid nitrogen and dried, before being resuspended in 80 μl ammonium bicarbonate 100 mM and digested with 1 μg of trypsin during 2 h at 37°C. Digestion reaction was stopped with 10 μl of 1% trifluoroacetic acid (TFA) and samples diluted with 80 μl of 2% acetonitrile in 0.05 % TFA. The pH was adjusted to 2.5-3.0 with 5 μl 10% TFA before mass spectrometry analyses.

Mass spectrometry analyses

Tryptic peptide mixtures were injected on a Dionex RSLC 3000 nanoHPLC system (Dionex, Sunnyvale, CA, USA) interfaced via a nanospray Flex source to a high resolution QExactive Plus mass spectrometer (Thermo Fisher, Bremen, Germany). Peptides were loaded onto a trapping microcolumn Acclaim PepMap100 C18 (20 mm x 100 μm ID, 5 μm, Dionex) before separation on a C18 custom packed column (75 μm ID × 50 cm, 1.8 μm particles, Reprosil Pur, Dr. Maisch), using a gradient from 4 to 76 % acetonitrile in 0.1 % formic acid for peptide separation (total time: 65min). Full MS survey scans and SIM scans between *m/z* 525.0-540.0 were performed at 70,000 resolution. In data-dependent acquisition controlled by Xcalibur software (Thermo Fisher), the 10 most intense multiply charged precursor ions detected in the full MS survey scan were selected for higher energy collision-induced dissociation (HCD, normalized collision energy NCE=27 %) and analysis in the orbitrap at 17'500 resolution. The window for precursor isolation was of 1.5 m/z units around the precursor and selected fragments were excluded for 60s from further analysis.

Data analysis

MS data were analyzed using Mascot 2.7 (Matrix Science, London, UK) set up to search the Swiss-Prot (https://www.uniprot.org/) database restricted to *Mus musculus* taxonomy (UniProt, November 2019 version: 17'034 sequences). Trypsin (cleavage at K,R) was used as the enzyme definition, allowing 4 missed cleavages. Mascot was searched with a parent ion tolerance of 10 ppm and a fragment ion mass tolerance of 0.02 Da. Carbamidomethylation of cysteine was specified in Mascot as a fixed modification. Acetylation and propionylation of lysine, N-terminal propionylation and methionine oxidation were specified as variable modifications. Signals corresponding to *m/z* of histone (H3.1) modified peptides (acetylation and propionylation), in particular the intensity of specific isotopic peaks corresponding to 13-C incorporation, were extracted with Xcalibur software and compared between samples.

ATAC-seq analysis

Murine T cells were activated as described above. ATAC-seq was performed as described in (Buenrostro et al., 2015). Briefly, 5*10^4 T cells were washed with cold 1xPBS and resuspended in 50μl of ice-cold lysis buffer (10mM Tris-Cl (pH 7.4), 10mM NaCl, 3mM $MgCl_2$ and 0.1% (v/v) of NP-40. Cells were centrifuged immediately and the resulting pellet (nuclei) was resuspended in 50μl of transposase reaction mix (25μl 2xTDbuffer, 2.5μl Tn5 transposase (Illumina) and 22.5μl of nuclease-free water), followed by incubation at 37°C for 30min. Tagmented DNA was cleaned using QIAGEN MinElute PCR Purification kit as described in the kit's protocol. Library preparation was performed using the custom Nextera PCR primers (FW: AATGATACGGCGACCACCGAGATCTACACTCGTCGGCAGCGTCAGATGTG, RV1:CAAGCAGAAGACGGCATACGAGATTCGCCTTAGTCTCGTGGGCTCGGAGATGT, RV2:CAAGCAGAAGACGGCATACGAGATCTAGTACGGTCTCGTGGGCTCGGAGATGT, RV3:CAAGCAGAAGACGGCATACGAGATTTCTGCCTGTCTCGTGGGCTCGGAGATGT, RV4:CAAGCAGAAGACGGCATACGAGATGCTCAGGAGTCTCGTGGGCTCGGAGATGT, RV5:CAAGCAGAAGACGGCATACGAGATAGGAGTCCGTCTCGTGGGCTCGGAGATGT) (Buenrostro et al., 2013) and NEBNext High-Fidelity 2X PCR Master Mix(M0541), using the following program: 5 min 72°C, 30 s 98°C; 10 cycles: 10 s 98°C, 30 s 63°C, 1 min 72°C. The libraries were then cleaned using Agencourt AMPure XP magnetic beads (A63880, Beckman). Finally, libraries were quantified using Fragment Analyzer and sequenced on an Illumina HiSeq 4000 device, with paired end 75 nucleotides at the Gene Expression Core Facility at the Ecole Polytechnique Fédéral Lausanne, Switzerland. Low quality reads were filtered and trimmed using AdapterRemoval (v. 2.1.7) (Schubert

et al., 2016). Reads were aligned to the *Mus musculus* genome (v. GRCm38, release 94) using Bowtie 2 (v. 2.3.4.1) (Langmead and Salzberg, 2012) with default parameters. Next, we used samtools to convert and sort sam files to sorted bam files, as well as to mark duplicates using the markdup function of samtools (v. 1.8) (Li et al., 2009). Finally, we retained reads aligning to the primary assembly only, removing the reads aligned to the mitochondrial chromosome or alternate locus groups. The callpeak function of MACS2 (v. 2.1.1.20160309) (Zhang et al., 2008) was used for peak calling, combining the sorted and filtered bam files of all replicates per condition, and using the –broad option. These computations were performed at the Vital-IT Center for High Performance Computing (https://www.vital-it.ch/) of the Swiss Institute of Bioinformatics.

Differential chromatin accessibility analysis between MPCi-treated and DMSO-treated samples was performed using R (v. 3.5.1) (https://www.r-project.org/). Number of reads overlapping each broad peak called by MACS2 were counted using the dba.count function of the DiffBind package (v. 2.10.0) (Ross-Innes et al., 2012) (http://bioconductor.org/packages/release/bioc/vignettes/DiffBind/inst/doc/DiffBind.pdf), with parameters minOverlap = 2 and score=DBA_SCORE_TMM_READS_FULL. The edgeR method implemented in the dba.analyze and dba.report functions of DiffBind was used to statistically compare the accessibility of genomic regions, considering the regions with an FDR<0.05 as statistically significant. Using the rGREAT package (v 1.14.0) (Gu, 2018). rGREAT: Client for GREAT Analysis. https://github.com/jokergoo/rGREAT, http://great.stanford.edu/public/html/), all chromatin regions were annotated to neighboring genes with the submitGreatJob function and options species="mm10" and version="3.0". In the genomic regions more open in MPCi-treated samples, transcription factor binding motif enrichment was determined using the findMotifsGenome.pl perl script of the Homer software (v. 4.11) (Heinz et al., 2010). Significant motifs were ranked based on the percentage of more-accessible regions (targets) in which they were present. To determine the location of the RUNX1 binding motif in more open regions in MPCi-treated samples, we first extracted the sequences of these regions as fasta format using the getfasta function of the BEDtools suite (Quinlan and Hall, 2010). We scanned these sequences for the RUNX1 motif (MA0002.2) downloaded from the Jaspar database (http://jaspar.genereg.net/) with the online FIMO program (Grant et al., 2011) of the MEME suite (https://meme-suite.org/meme/doc/fimo.html?man_type=web). Finally, using the ChIPpeakAnno package (v. 3.16.1) (Zhu et al., 2010), genomic locations of the RUNX1 motif were annotated to neighboring genes with the annotatePeakInBatch function with default parameters and the mouse TSS annotation data (TSS.mouse.GRCm38), and gene symbols were obtained with the addGeneIDs function.

A signature of memory precursor cells was compiled from Dominguez et al, and used for gene set enrichment analysis. All genomic regions included in the peak set annotated to genes were sorted according to decreasing fold change. When more than 1 peak was assigned to a same gene, only the peak with highest absolute fold change value was retained. Sorted fold change values were provided to the GSEA function of the clusterProfiler package (v. 4.0.4) (Wu et al., 2021) in R (v. 4.1.0), using options eps = 1e-50, and seed=T (seed=1234).

RUNX1 deletion by CRISPR-Cas9

3 small guide RNA (gRNA) for murine *Runx1* (sequence 1: CGGTCCCTACACTAGGACAT, sequence 2: TGCGCACTAGCTCGCCAGGG, and sequence 3: CCAGCGACACCCATTTCACC) were designed using the publicly available gRNA design tool which improves on-target activity and reduces off-target activity (Doench et al., 2016). The gRNA's were then cloned into a SIN-inactivated version of the MSCV retroviral vector, driving gRNA expression by the pU6 promoter. Retroviral particles were generated by co-infecting Phoenix-Eco cells with the gRNA retroviral plasmids and pCL-ECO. Viral particles were concentrated by ultracentrifugaton and the 3 different gRNA viruses were pooled, aliquoted and stored at -80°C.

$CD8^+$ T cells were isolated from spleens of conditional Cas9.*Cd4-Cre* OT1 mice and activated with Activator CD3/CD28 Dynabeads (Gibco) at a 1-cell:2-beads ratio in completed RPMI containing 50 IU/ml rhIL2. 24 hours later, T cells were transduced with either concentrated retrovirus expressing a scrambled small guiding RNA (gRNA SCR) or the pool of 3 different gRNA targeting *Runx1*. 48 hours after transduction, T cells were further expanded in either DMSO or 20μM UK5099 (MPCi), always in completed RPMI containing 50 IU/ml rhIL2. 5 days later, activation beads were magnetically removed and transduced cells were purified based on their Thy1.1 expression (Stem Cell Technologies).

Flow cytometry

All fluorochrome-conjugated antibodies were from Biolegend of Thermo Fisher Scientific; non-conjugated antibodies were from Cell Signaling. Flow cytometry staining was performed in PBS containing 2% FBS + 2mM EDTA, on ice. Dead cells were excluded with the Life/Dead Fixable Blue Cell Stain Kit (Thermo Fisher Scientific). Intracellular staining was performed using the Foxp3 staining kit (Thermo Fischer Scientific) according to the manufacturer's instructions. Samples were acquired on LSR II or Fortessa flow cytometers with FACSDiva Software.

HER2 CAR T cells were identified by staining surface HER2-CAR using a recombinant human Fc-tagged Her2/ErbB2 Protein (Sino Biological) followed by an anti-human-Fc Alexa Fluor 488 conjugate (Thermo Fisher Scientific).

Western blot

Cells were lysed in RIPA lysis buffer (50 mM TrisHCl pH8, 150 mM NaCl, 1% Triton X 100, 0.5% Sodium deoxycholate, 0.1% SDS), freshly supplemented with 500 μM protease inhibitor (4-(2-Aminoethyl)benzenesulfonyl fluoride hydrochloride, Sigma) and 1x phosphatase inhibitor (Phosstop, Sigma). Histones were isolated as described above and in Lund et al. (2019). Proteins were quantified by BCA protein assay kit (Thermo Scientific) and equal amounts of proteins were denatured with at 95°C for 5 minutes. Proteins were separated on 7.5% or 12.5% polyacrylamide gradient gels and transferred onto nitrocellulose membranes 0.2 μm

CellPress
OPEN ACCESS

(Biorad). Non-specific binding sites were blocked in milk 5% or 5% bovine serum albumin (Sigma) and membranes were incubated with primary antibodies (Cell Signaling and GeneTex). Membranes were then incubated with HRP-labeled secondary anti-rabbit (1:1000) and anti-mouse (1:10’000) antibodies (Santa Cruz Biotechnology), and blots were visualized by chemiluminescence with ECL and Femto reagents (Super Signal West, Thermo Scientific).

QUANTIFICATION AND STATISTICAL ANALYSIS

Flow cytometry data was analysed using FlowJo v10 on appropriate gated cells after removal of doublets and dead cells. Western blots were quantified with ImageJ software. Statistical Analysis was performed using Prism v9 software (GraphPad). Results are represented as mean ± standard error of mean (SEM). Statistical details are provided in figure legends, as well as sample size and number of independent repeats. Briefly, comparisons for two groups were calculated using unpaired two-tailed Student’s t tests. Comparisons of more than two groups were calculated using one-way ANOVA with Tukey multiple comparison correction. Comparisons of grouped data were calculated using two-way ANOVA with the original false discovery rate method of Benjamini and Hochberg. Sample size was estimated based on previous experience. Mice were excluded from analyses in the rare event of graft rejection. Samples were excluded from flow cytometric analyses when less than 20 events were recorded in the cell population of interest, not allowing for precise phenotypic evaluation, except when performing absolute quantification of that cell population of interest.

Cell Metabolism, Volume *34*

Supplemental Information

The mitochondrial pyruvate carrier regulates memory T cell differentiation and antitumor function

Mathias Wenes, Alison Jaccard, Tania Wyss, Noelia Maldonado-Pérez, Shao Thing Teoh, Anouk Lepez, Fabrice Renaud, Fabien Franco, Patrice Waridel, Céline Yacoub Maroun, Benjamin Tschumi, Nina Dumauthioz, Lianjun Zhang, Alena Donda, Francisco Martín, Denis Migliorini, Sophia Y. Lunt, Ping-Chih Ho, and Pedro Romero

Supplemental Information

Supplemental figure legends

Figure S1. MPC inhibition in CD8$^+$ T cells induces alternative metabolic fluxes that results in an epigenetic crosstalk and memory imprinting

(A-B) Schematic presentation of the culture conditions for metabolomics analysis (A). Naïve CD8$^+$ T cells were activated with anti-CD3/CD28 antibodies in the presence of DMSO or UK5099 (MPCi). After 72 hours, the cells were harvested for untargeted metabolomics analysis by mass spectrometry. All detected metabolites are shown and scaled to the average of the DMSO samples (B). "(NG)" before a metabolite means, no confident reliable detection was possible ("Not Good"). (n = 4 biological replicates)

(C-H) Fresh naïve CD8$^+$ T cells were activated with anti-CD3/CD28 antibodies in the presence of DMSO or MPCi. After 66 hours, T cells were washed and incubated with medium containing either 11mM U-^{13}C-glucose, 4mM U-^{13}C-glutamine, or 120 μM U-^{13}C-Palmitate. 6 hours later, ^{13}C incorporation was measured by mass spectrometry. Shown here are the isotopic ratios (C-E) and labelled abundances (G-H) of citrate. ('m+n' equals the molecular mass plus the number of incorporated heavy carbons). (n = 4 biological replicates/group)

Data is represented as mean ± standard error of mean. Statistics are based on two-way ANOVA using the original false discovery rate method of Benjamini and Hochberg, with *p < 0.05, **p < 0.01, ***p < 0.001, ****p < 0.0001 and ns (p > 0.05).

Figure S2. MPC inhibition in CD8$^+$ T cells induces alternative metabolic fluxes that modulate chromatin epigenetic modifications and memory imprinting

(A) OT1 splenocytes were activated with SIINFEKL and IL2 in the presence of DMSO or UK5099 (MPCi). After 72h, protein was collected for western blot analysis of acetylated lysine residues on whole cell lysate, and beta-actin as loading control. Shown here is the same blot with either short or long exposure time allowing for a proper discrimination of all bands. The estimated histone region is indicated (containing histones H2A/B, H3 and H4).

(B-C) Representative western blot (B) and quantification (C) of modified lysine residues on histone 3, normalized for total histone 3 (H3) protein and shown as fold change compared to DMSO. (n = 2 biological replicates, pooled data from 2 independent experiments)

(D-E) Isotopic distribution of acetylated peptide KSAPATGGVKKPHR (containing H3K27) at *m/z* 529.975 with ^{12}C (D) or ^{13}C (E) glucose as substrate. Non-acetylated lysines were propionylated. Monoisotopic peak (containing only ^{12}C) and 3rd isotopic peak (2*^{13}C) were used for measurement of ^{13}C incorporation into the peptide due to the acetylation. The increase of other isotopic peaks at higher m/z indicates that ^{13}C incorporation can occur not only because of the acetylated group, but also in the peptide backbone, although at a lower level.

(F) Volcano plot from ATAC sequencing data showing the genes associated to more closed (left) or more open (right) chromatin regions upon MPCi treatment. Genes appearing in the KEGG pathways fatty acid oxidation (FAO), glutamine oxidation (GO), pentose phosphate pathway (PPP), glycolysis and oxidative phosphorylation (oxphos) are highlighted in color.

Data is represented as mean ± standard error of mean. Statistics are based on two-way ANOVA using the original false discovery rate method of Benjamini and Hochberg, with ns ($p > 0.05$).

Figure S3. Runx1 orchestrates CD8$^+$ T cell memory differentiation upon mitochondrial pyruvate carrier inhibition

(A) CAS9-expressing CD8$^+$ OT1 T cells were activated and transduced with either scrambled small guiding RNA (gRNA SCR) constructs or a pool of 3 different gRNA's targeting *Runx1*. 48 hours after transduction, T cells were further expanded in either DMSO or 20μM UK5099 (MPCi). 7 days post-transduction, western blot was performed on the whole cell lysate. Total histone H3 was used as loading control. (representative western blot of 2 independent experiments with 1 biological replicate each)

Figure S4. Mitochondrial pyruvate carrier deletion in CD8$^+$ T cells blunts their anti-tumor potential

(A-D) Tumor and spleen single cell suspensions were restimulated *ex vivo* with SIINFEKL for 4 hours. Transferred WT or MPC1 KO CD8$^+$ T cells were assessed for expression of cytokines or CD107a by flow cytometry. Data is expressed as percentage out of transferred cells (A-D) or absolute number of cells per mg of tumor (E-J) (n = 16 mice/group, pooled data from 3 independent experiments (A-I) and n = 10-12 mice/group, pooled data from 2 independent experiments (J))

Data is represented as mean ± standard error of mean. Statistics are based on two-way ANOVA using the original false discovery rate method of Benjamini and Hochberg (A-D), or on unpaired, two-tailed Students t-test (E-J) with *p < 0.05, **p < 0.01, ***p < 0.001, ****p < 0.0001 and ns (p > 0.05).

Figure S5. Tumor-infiltrating CD8$^+$ T cells oxidize lactate in their mitochondria to maintain effector function

(A) Schematic presentation of the *in vitro* experimental modelling of a tumor nutrient-deprived microenvironment. WT or MPC1 KO OT1 splenocytes were activated with SIINFEKL and IL2. Cells were washed and split after 3 days, and then cultured for another 3 days. The T cells were then collected, counted and placed in nutrient-rich or nutrient-deprived medium with varying concentrations of sodium lactate, or sodium chloride as osmotic control for 18 hours and then restimulated with SIINFEKL for 4 hours.

(B) Viability of the 18h starved WT and MPC1 KO CD8$^+$ T cells was analyzed by flow cytometry before and 4 hours after restimulation with SIINFEKL. Shown here is the percentage of live cells, identified as AnnexinV-negative and negative for the dead cell staining dye. (n = 4 biological replicates/genotype, pooled data from 2 independent experiments)

(C-E) Resting antigen-experienced WT or MPC1 KO OT1 CD8$^+$ T cells were placed in nutrient-rich or nutrient-deprived medium with varying concentrations of lactate for 18 hours and then restimulated with SIINFEKL for 4 hours. T cells expressing IFNγ (C), TNF (D) or IL2 (E) were analyzed by flow cytometry. (n = 5 biological replicates/genotype, pooled data from 2 independent experiments)

(F) Naïve CD8$^+$ T cells were isolated from spleens of WT or MPC1 KO OT1 mice and were activated with anti-CD3/CD28-coated beads for 3 days and then rested for an additional 3 days. The T cells were then placed in nutrient-rich or nutrient-deprived medium with varying concentrations of lactate for 18 hours and then restimulated with SIINFEKL for 4 hours. T cells expressing IFNγ, TNF and IL2 were analyzed by flow

cytometry. (n = 2 biological replicates/genotype, pooled data from 2 independent experiments)

(G-J) Resting antigen-experienced WT CD8$^+$ T cells were placed in nutrient-deprived medium with or without lactate for 18 hours in the presence of 25μM LDHA/B inhibitor, GSK 2837808A (LDHi), or DMSO control and then restimulated with SIINFEKL for 4 hours. T cells expressing IFNγ (G), TNF (H), IL2 (I) or co-express both IFNγ and TNF (J) were analyzed by flow cytometry. (n = 2 biological replicates/group, pooled data from 2 independent experiments)

(K) Resting antigen-experienced WT or MPC1 KO CD8$^+$ T cells were placed in nutrient-rich or nutrient-deprived medium with or without lactate and varying concentrations of the mTOR inhibitor Torin2 for 18 hours and then restimulated with SIINFEKL for 4 hours. Western blot analysis shows mTOR pathway activity. Different phosphorylation status of 4E-BP1 results a separation in 3 obvious bands with the α-form being unphosphorylated and the γ-form most phosphorylated. (Representative blot from 3 independent experiments with 1 biological replicate/experiment)

(L-O) Resting antigen-experienced WT or MPC1 KO CD8$^+$ T cells were placed in nutrient-rich or nutrient-deprived medium with or without lactate and varying concentrations of the mTOR inhibitor Torin2 for 18 hours and then restimulated with SIINFEKL for 4 hours. T cells expressing IFNγ (L), TNF (M), IL2 (N) or co-express both IFNγ and TNF (O) were analyzed by flow cytometry. (n = 2 biological replicates/genotype, pooled data from 2 independent experiments)

Data is represented as mean ± standard error of mean. Statistics are based on two-way ANOVA using the original false discovery rate method of Benjamini and Hochberg, with *p < 0.05, **p < 0.01, ***p < 0.001, ****p < 0.0001 and ns (p > 0.05). For clarity, only relevant statistical comparisons are shown on graphs.

Figure S6. Mitochondrial pyruvate carrier inhibition during CAR T cell *in vitro* activation and expansion induces superior anti-tumor activity upon adoptive cell transfer in a mouse melanoma model

(A) CD45.1$^+$ OT1 CD8$^+$ T cells were activated and expanded for 7 days with DMSO or 20μM UK5099 (MPCi) (called here "conditioning", in preparation for ACT), after which the small molecule was washed out and the cells were then transferred into B16-SIINFEKL tumor-bearing CD45.2$^+$ mice. At the point of adoptive cell transfer, mice received a subcutaneous administration of a vaccine consisting out of SIINFEKL and synthetic oligodeoxynucleotides containing CpG motifs. Shown here is the tumor weight measured upon dissection at day 26.

(B) Total number of transferred OT1 CD8$^+$ T cells in the tumor-draining lymph node, measured by flow cytometry.

(C-E) Percentage of tumor infiltrating transferred OT1 CD8$^+$ T cells expressing PD1 (C), LAG3 (D) or TIM3 (E), measured by flow cytometry.

(F-K) Spleen single cell suspensions were restimulated with SIINFEKL *in vitro* for 4 hours in the presence of Golgi inhibitors. The percentage of transferred cells expressing cytokines and granzyme B (F, H, J) or their absolute numbers (G, I, K) were measured by flow cytometry.

(L-N) Tumor single cell suspensions were restimulated with SIINFEKL *in vitro* for 4 hours in the presence of Golgi inhibitors. The percentage of transferred cells expressing cytokines and granzyme B (L, M) or their absolute numbers (N) were measured by flow cytometry. ((A-N) n = 11 mice (DMSO) and 14 mice (MPCi), pooled data from 2 independent experiments.)

(O) Schematic representation of the experiment. Polyclonal CD8$^+$ T cells were activated in the presence of DMSO or 20μM UK5099 (MPCi) and transduced with either a control blue fluorescent protein-expressing vector (BFP) or an anti-HER2 CAR. The T cells were expanded for 7 days in the presence of DMSO or MPCi and then transferred into mice bearing HER2-expressing B16 tumors.

(P) Transduction efficiency measured by flow cytometry of polyclonal CD8$^+$ T cells with an anti-HER2 CAR in the presence of DMSO or MPCi.

(Q) At the time of adoptive cell transfer, histones from DMSO- or MPCi-conditioned HER2 CAR T cells were analyzed by Western blot.

(R-S) Number of DMSO- or MPCi-treated HER2-CAR-positive cells (R) and their percentage of short-lived effector cells (S) in the blood 12 days after adoptive cell transfer, measured by flow cytometry. (n = 12-13 mice (DMSO- and MPCi-treated HER2-CAR T cell transfer), pooled data from 2 independent experiments.)

Data is represented as mean ± standard error of mean. Statistics are based on unpaired, two-tailed Students t-test, with *p < 0.05, **p < 0.01, and ns (p > 0.05).

Figure S7. Mitochondrial pyruvate carrier inhibition induces memory differentiation in human T cells and dramatically improves CD19-CAR T cell therapy against acute lymphoblastic leukemia

(A-C) Human peripheral blood mononuclear cells (PBMC) were activated with anti-CD3/CD28 beads for 5 days and then cultured another 4 days before phenotypic analyses. The cells were exposed to DMSO or UK5099 (MPCi) during the entire time of culture. Percentage CD62L-positive CD4$^+$ T cells (A), mean fluorescent intensity of CD62L in the CD62L-positive population (B), the percentage of stem cell-like memory

$CD4^+$ T cells (C) was measured by flow cytometry. (n = 7 human donors/group, pooled data from 2 independent experiments)

(D) Transduction efficiency of human T cells activated and cultured for 7 days in the presence of DMSO or MPCi, assessed by Protein-L measurement by flow cytometry.

(E) The body weight of NOD scid γ (NSG) mice bearing advanced systemic NALM6 leukemia xenografts left either untreated, or treated with non-transduced (NTD) or anti-CD19 CAR (CAR) T cells conditioned with DMSO or UK5099 (MPCi). (n = 4 mice for untreated, n=5 mice for NTD DMSO and NTD MPCi, n = 7 mice for CAR DMSO and n = 8 mice for CAR MPCi, pooled data from 2 independent experiments)

(F-H) Number of $CD4^+$ T cells (F) or $CD8^+$ T cells (G) per μl of blood in NSG mice treated with MPCi-conditioned anti-CD19 CAR T cells. Flow cytometry analysis was performed on blood samples collected at the indicated number of days following adoptive cell transfer (ACT). Shown in (H) is a representative dot blot graph showing CD45RO and CD62L expression in $CD8^+$ T cells 27 days post-ACT, allowing the detection of effector memory (EM), central memory (CM) and stem cell-like memory (SCM) T cells. (n = 8 mice, pooled data from 2 independent experiments)

Data is represented as mean $\pm$ standard error of mean. Statistics are based on paired, two-tailed Students t-test (A-C), with *$p < 0.05$, **$p < 0.01$, ***$p < 0.001$.

Figure S1, related to Figure 2

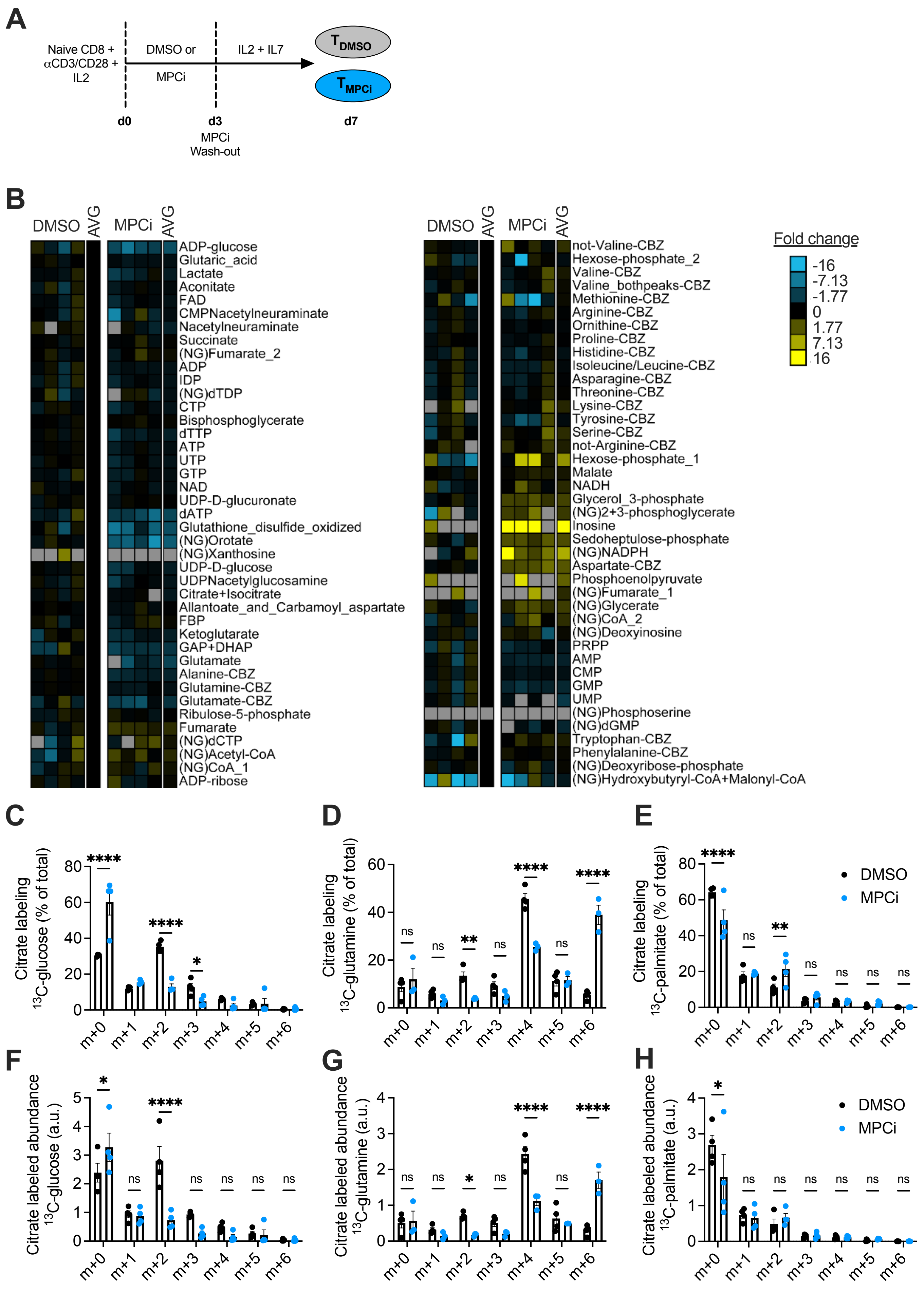

Figure S2, related to Figure 2

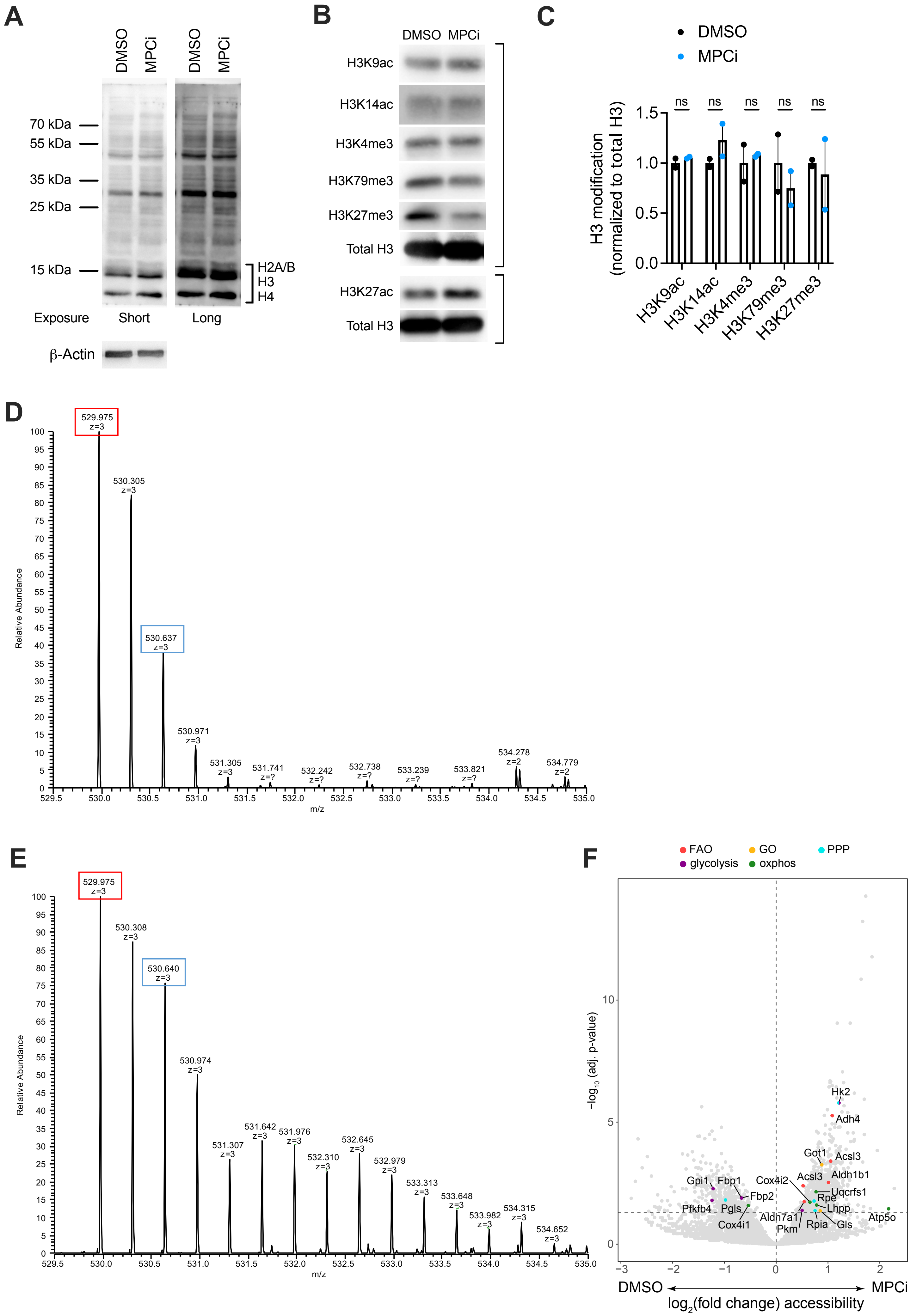

Figure S3, related to Figure 3

A

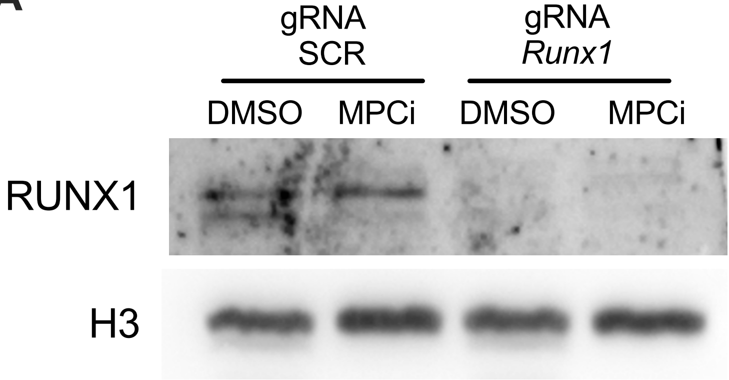

Figure S4, related to Figure 4

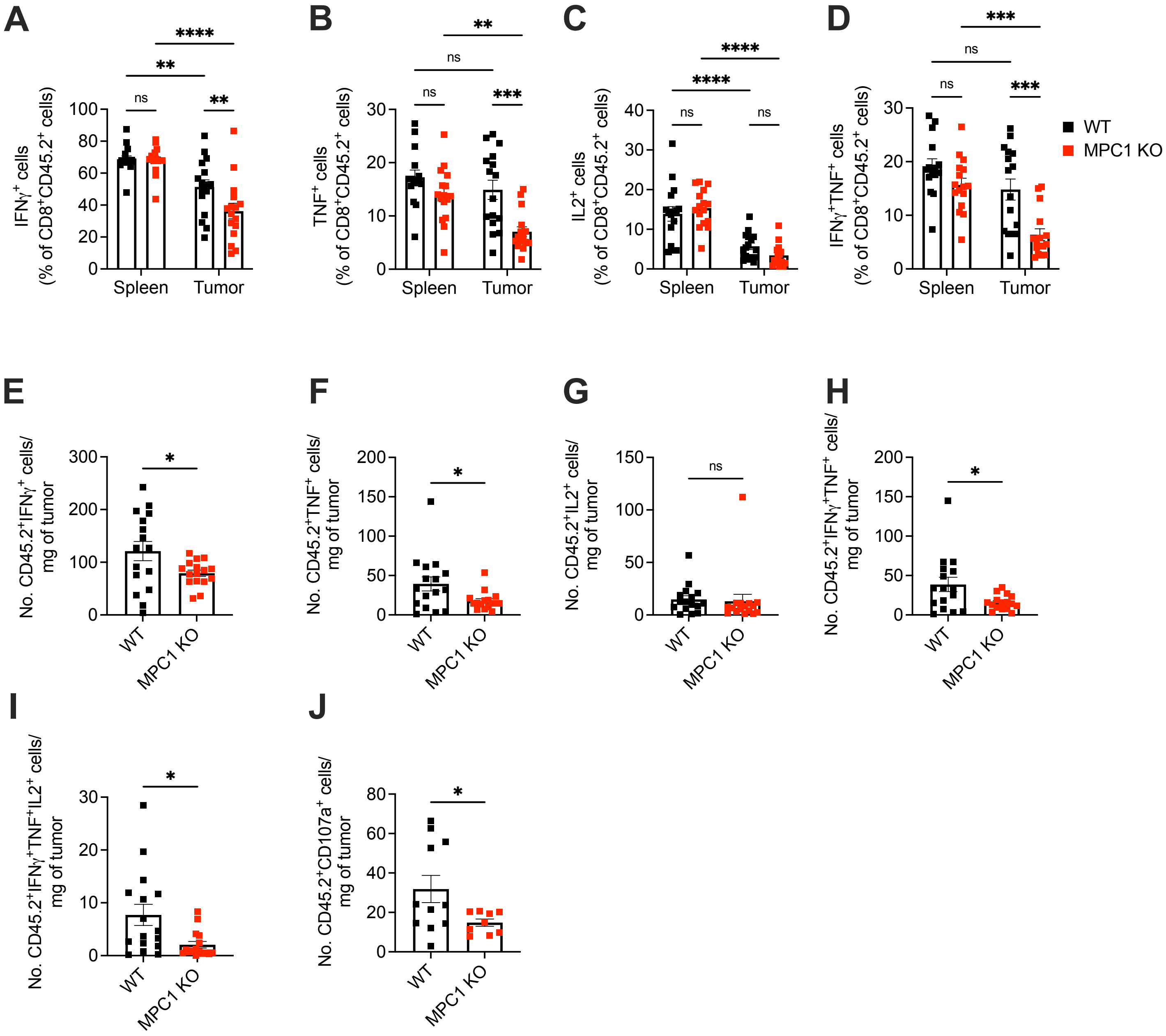

Figure S5, related to Figure 5

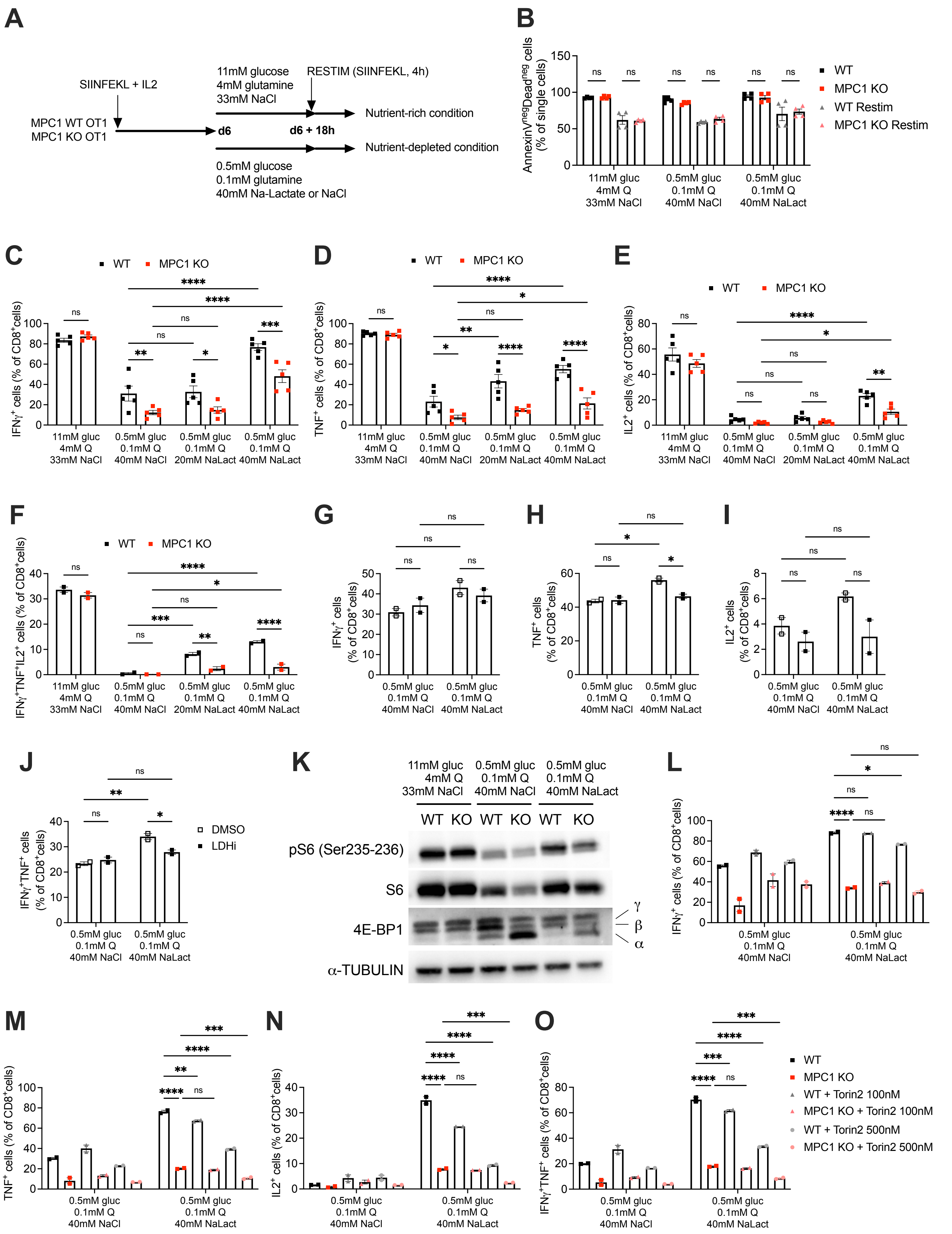

Figure S6, related to Figure 6

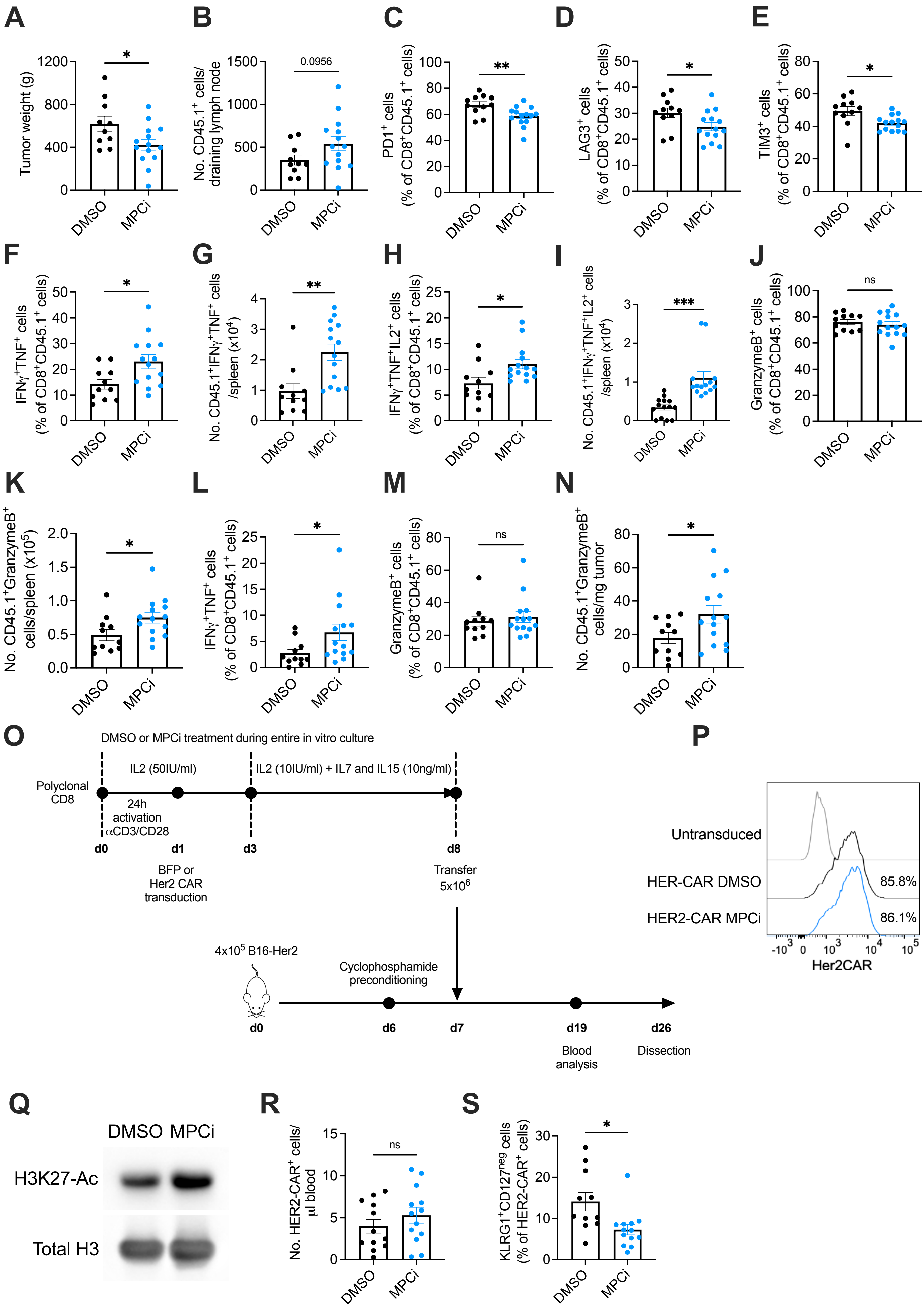

Figure S7, related to Figure 7

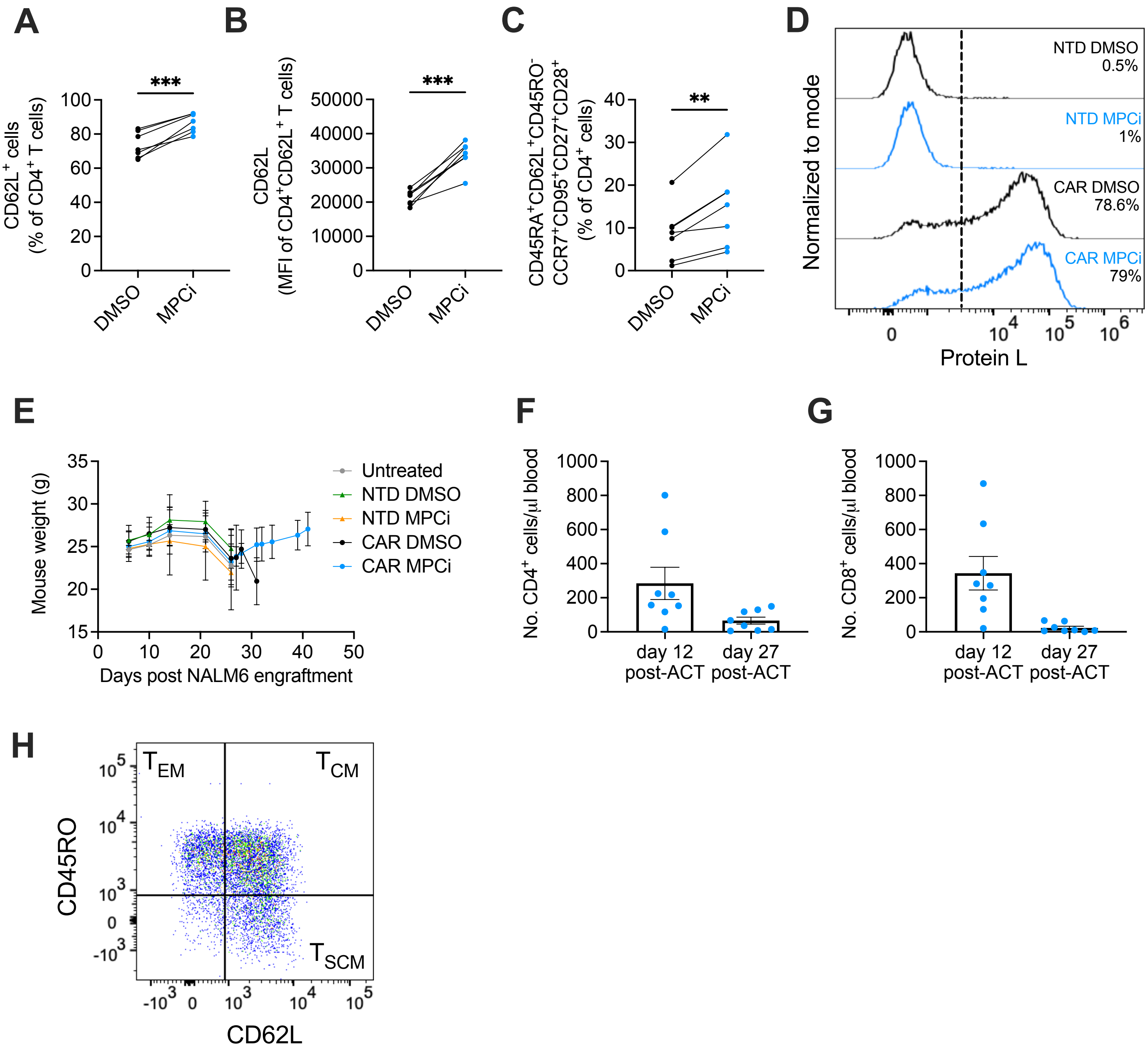

Supplement: Document S2. Article plus supplemental information [file mmc4.pdf]
